# Augmented prediction of multi-species protein–RNA interactions using evolutionary conservation of RNA-binding proteins

**DOI:** 10.1038/s41467-026-72351-6

**Published:** 2026-04-27

**Authors:** Jiale He, Tong Zhou, Lu-Feng Hu, Yuhua Jiao, Junhao Wang, Shengwen Yan, Siyao Jia, Qiuzhen Chen, Wentao Zhu, Jilin Zhang, Mutian Jia, Yuanning Li, Xianwei Wang, Yangming Wang, Yucheng T. Yang, Lei Sun

**Affiliations:** 1 Shandong Provincial Key Laboratory of Development and Regeneration, School of Life Sciences, Shandong University, Qingdao, China; 2 State Key Laboratory of Gene Function and Modulation Research, Institute of Molecular Medicine, College of Future Technology, Peking University, Beijing, China; 3 Beijing Advanced Center of RNA Biology (BEACON), Peking University, Beijing, China; 4 Interdisciplinary Center, Shandong University, Qingdao, China; 5 Department of Biomedical Sciences, College of Biomedicine, City University of Hong Kong, Kowloon Tong, Hong Kong SAR China; 6 Department of lmmunology, School of Basic Medical Science, Cheeloo College of Medicine, Shandong University, Jinan, Shandong China; 7 Institute of Marine Science and Technology, Shandong University, Qingdao, China; 8 State Key Laboratory of Microbial Technology, Shandong University, Qingdao, China; 9 College of Biomedical Engineering, Fudan University, Shanghai, China; 10 MOE Key Laboratory of Computational Neuroscience and Brain-Inspired Intelligence, and MOE Frontiers Center for Brain Science, Fudan University, Shanghai, China

**Keywords:** Computational biology and bioinformatics, Molecular biology, Biological techniques

## Abstract

RNA-binding proteins (RBPs) play critical roles in the regulation of gene expression. Recent studies have begun to detail the RNA recognition mechanisms of diverse RBPs. However, given the array of RBPs studied so far, it is implausible to experimentally profile RBP-binding peaks for hundreds of RBPs in multiple non-model organisms. Here, we introduce MuSIC (**Mu**lti-**S**pecies RBP–RNA **I**nteractions using **C**onservation), a deep learning-based framework for predicting cross-species RBP–RNA interactions by leveraging label smoothing and evolutionary conservation of RBPs across 11 phylogenetically diverse species ranging from human to yeast. MuSIC outperforms state-of-the-art computational methods, and achieves highly accurate prediction of RBP-binding peaks across species. The prediction confidence is higher in the metazoan species, partially reflecting differences in RBP conservation patterns. Finally, the effects of homologous genetic variants on RBP binding can be computationally quantified across species, followed by experimental validations. The target transcripts with disrupted binding events are enriched in the ubiquitination-associated pathways. To summarize, MuSIC provides a useful computational framework for predicting RBP–RNA interactions cross-species and quantifying the effects of genetic variants on RBP binding, offering insights into the RBP-mediated regulatory mechanisms implicated in human diseases.

## Abstract (web summary)

RNA-binding proteins (RBPs) regulate gene expression, but predicting RBP–RNA interactions across species is challenging. Here, the authors introduce MuSIC, a deep learning framework leveraging label smoothing and evolutionary conservation across 11 species to predict cross-species RBP–RNA interactions.

## Introduction

RNA-binding proteins (RBPs) play an important role that interact with intracellular RNAs by recognizing specific RNA motifs, and perform important biological functions including RNA transcription, splicing, transport, translation, and stability^1–3^. Disruptions in RBP–RNA interactions have been implicated in the pathogenesis of various diseases, particularly cancer and neurodegenerative diseases^4,5^. Genetic variants, including single nucleotide polymorphisms (SNPs) and single nucleotide variants (SNVs), can alter the RBP-binding affinity, thereby disrupting RNA recognition and contributing to the development of various diseases^6–8^. For example, the genetic variants in the iron-responsive element of *FTL* can impair the binding of IRP1 to *FTL* mRNA, leading to hyperferritinaemia-cataract syndrome^9^. Similarly, the genetic variants in *TCF3* can hinder the binding of hnRNPH1 to exon 18b, thus disrupting alternative splicing of *TCF3* and promoting Burkitt lymphoma development^10^. These cases highlight the critical roles of RBP–RNA interactions in human disease pathogenesis.

Model organisms, such as mouse and zebrafish, are widely used in revealing the pathogenic mechanisms of complex human diseases^11,12^, where normal RBP–RNA interactions could be potentially disrupted by genetic variants^13,14^. Various high-throughput experimental methods have been applied to identify RBP-binding peaks both in vitro^15–17^ and in vivo^18–21^. For example, PAR-CLIP technology has been applied to the mouse for exploring the regulatory roles of IGF2BP3 and LIN28B in hematopoietic reprogramming^22^. However, it is still unrealistic to generate the binding profiles of hundreds of RBPs in human and animal models using in vivo high-throughput experimental methods.

With the recent advancements in AI technologies, computational methods leveraging large-scale RBP–RNA binding data can effectively learn RBP binding characteristics^19,23,24^. Several computational methods using machine learning^25,26^ and deep learning^27–32^ technologies have been developed to predict RBP-binding peaks and model their RNA sequence preferences. However, the predictions by these traditional computational methods are generally restricted in the specific species, where the CLIP-seq datasets are generated. Given the fact that only a minority of the RBPs that have been studied by CLIP-seq technologies are from non-human organisms, 65 RBPs identified in yeast, 45 in mouse, and only 5–6 in species such as fly, worm, and *A. thaliana*^33^, it is vital to develop the computational methods to predict RBP–RNA interactions in the cross-species tasks^34,35^.

The style transfer methods have been successfully applied in the field of computer vision, where similarity-guided label smoothing strategy could improve the cross-style performance^36–38^. In our study, the analogies of the similarity features in label smoothing can be the sequence and structural conservation of RBPs. RBPs have been shown to be relatively highly conserved across species, particularly for the core RNA-binding domains (RBDs)^39–41^. Consistently, the RBP-binding motifs from the RBPs containing highly conserved RBDs also show notable degree of conservation^42–44^. These observations inspire us to apply the label smoothing strategy to the prediction of cross-species RBP–RNA interactions.

Recent advances in foundation models trained on large-scale unlabeled sequences enable the learning of transferable representations, which have substantially improved performance in RNA structure inference and protein–RNA interaction modeling^45–48^. Notably, several recent methods such as PaRPI and HDRNet demonstrate that the dynamic embeddings derived from pre-trained language models can effectively enrich sequence representations and therefore improve RBP binding predictions for diverse RBPs from different cell lines and species^31,32^. Inspired by these advances in computational modeling, we construct a computational framework to encode RNA and RBP sequences by incorporating existing RNA and protein foundation models, which could have the capability to model dynamic RBP–RNA interactions across different species.

In this study, we develop MuSIC (**Mu**lti-**S**pecies RBP–RNA **I**nteractions using **C**onservation), a deep learning framework for cross-species RBP–RNA interaction prediction by leveraging the pre-trained foundation models and the multi-level conservation patterns of RBPs. We show that MuSIC can accurately predict RBP-binding peaks across species and outperforms existing state-of-the-art computational methods in both closely and distantly related species. We then apply MuSIC to systematically profile the RBP-binding peaks and motifs for 184 RBPs across 11 species. Finally, we use MuSIC to quantify the effects of SNVs on RBP binding, providing insights into the RBP-mediated regulatory mechanisms associated with human diseases.

## Results

### Evolutionarily conserved RBPs exhibit similar RNA-binding preference

**Fig. 1:**
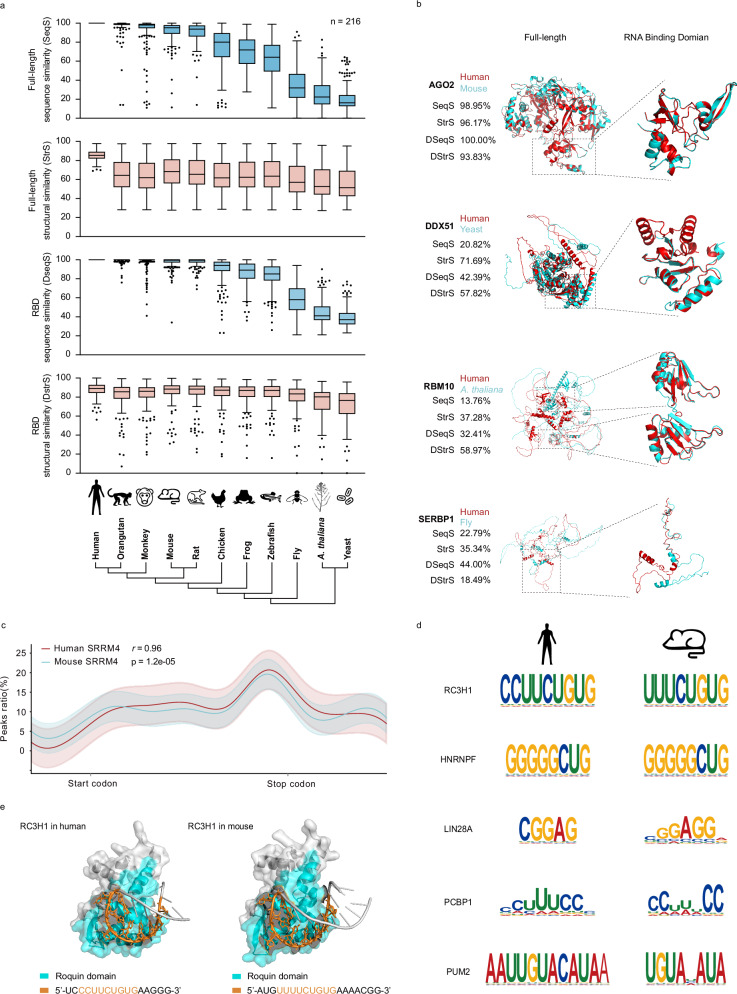
Evolutionary conservation of RBPs and their target RNAs. **a** Box plots showing the sequence (blue) and structural (red) similarity of RBPs across 11 species (*n* = 216 RBPs; center line, median; box limits, 25th and 75th percentiles; whiskers, 1.5 × interquartile range; points beyond the whiskers, outliers), in terms of full-length sequence (SeqS), full-length structure (StrS), RBD sequence (DseqS) and RBD structure (DstrS). The phylogenetic tree of the 11 species is reconstructed based on their 18S rRNA sequences. **b** Examples showing structural alignment of homologous RBPs, including AGO2 (the highly conserved), DDX51 (the considerable conserved), RBM10 (the intermediately conserved), and SERBP1 (the weakly conserved). The RBP structures from human (red) are compared to their homologs from non-human species (blue). **c** SRRM4 binding distribution along meta-transcript from human and mouse. Data are presented as mean values +/- SD (Spearman’s rank correlation test). **d** Binding motif alignment of homologous RBPs from human and mouse. **e** Three-dimensional structure of RC3H1 from human (PDB ID 4QIL, left) and mouse (PDB ID 4QI2, right), showing the residues of bound RNA (orange), the residues of RBD (blue), and the other residues of RNA and RBP (grey). Source data are provided as a Source Data file.

Previous studies have revealed that RBDs, the core functional elements in RBPs, exhibit a significantly slower rate of sequence evolution compared to genomic sequence context surrounding these RBDs, indicating their critical functions in regulating RNA recognition^39^. We collected protein sequences and predicted structures of 216 RBPs from 11 species ranging from human to yeast, including human, orangutan, monkey, mouse, rat, chicken, frog, zebrafish, fly, *A. thaliana* and yeast, to systematically assess the conservation patterns of RBPs and their core RBDs (see “Methods” for details). Briefly, the conservation patterns of RBPs and their core RBDs was evaluated based on sequence and structure similarity, respectively (Fig. 1a, Supplementary Fig. 1 and Supplementary Data 1). As expected, both sequence and structural similarities between human and non-human species declined with the increasing evolutionary distance (Fig. 1a). In particular, the protein sequence exhibited higher conservation for the core RBDs compared to the full-length RBPs; however, the structural similarity for both core RBDs and full-length RBPs remained relatively high across the 11 species ranging from human to yeast (Fig. 1a and Supplementary Fig. 1), indicating the fundamental importance of local structures of core RBDs for RNA recognition.

We then sought to partition the 216 RBPs into different groups based on their conservation patterns across the 11 species (Supplementary Data 2) and showed cases in each group (Fig. 1b): (i) highly conserved (1169, accounting for 49.0%), showing conservation in both sequence and structure in closely related species such as human and mouse, exemplified with the case of AGO2 protein; (ii) considerable conserved (769, accounting for 32.2%), showing conservation in full-length and RBD structure, such as DDX51; (iii) intermediately conserved (426, accounting for 17.8%), showing conservation in only RBD structure, such as RBM10; and (iv) weakly conserved (23, accounting for 1.0%), such as SERBP1. We found that almost all the RBPs ( > 99% of the total) exhibit structural conservation in their RBDs, consistent with previous studies showing that RBDs of the same RBP are highly structurally conserved across species^49,50^.

Previous studies have shown that RBPs containing structurally conserved RBDs tend to recognize and bind their RNA target with similar sequence patterns^42,50,51^. We first compared the distribution of RBP-binding peaks from the homologous RBPs along mRNAs in human and mouse, exemplified with the case of SRRM4 and LIN28A (Fig. 1c and Supplementary Fig. 2a). As expected, we observed a high correlation of the RBP-binding peak distributions between human and mouse (SRRM4 *r* = 0.96, LIN28A *r* = 0.91, Spearman’s rank correlation test). Notably, SRRM4 showed stronger preference of binding on the stop codon and a depletion around the start codon (Fig. 1c), suggesting the critical roles of SRRM4 in regulating alternative splicing of RNA within these regions^52^. Next, we explored the motif-level conservation of RBPs using the ATtRACT database^53^, which contains experimentally validated binding motifs in multiple species. Comparative analysis of RBP-binding motifs between human and mouse revealed a high degree of conservation (e.g., UUCUGUG motif for RC3H1, GGGGGCUG motif for HNRNPF, GGAG motif for LIN28A) (Fig. 1d). Moreover, both the structures derived from X-ray diffraction (Fig. 1e) and AlphaFold3^54^ prediction (Supplementary Fig. 2b–e) suggest that the homologous RBPs from different species exhibit similar three-dimensional interaction patterns between the interface residues in RBPs and their recognized target binding motifs (PDB ID 4QIL in human and 4QI2 in mouse) (Fig. 1e). Taken together, these results suggest that RBPs show high sequence and structural conservation across species, and maintain high conservation in their RNA-binding patterns, including motifs, binding regions within transcripts, and key interacting residues.

Inspired by the aforementioned observations and previous studies^30,34,44^, we aim to develop a computational model to systematically predict the RBP–RNA interactions in the target species (e.g., non-human species) by integrating the RBP-binding peaks from the source species (e.g., human) and the RBP similarity patterns between species. We leveraged the large-scale RBP-binding peaks from different species (human, mouse, zebrafish, and fly) to construct the computational model. We initially found 23 homologous RBPs between human and mouse, one homologous RBP between human and zebrafish from POSTAR3^33^, and three homologous RBPs between human and fly with available datasets from the modENCODE project^55^ (Supplementary Fig. 2f, g). In total, 44 datasets from 17 RBPs were curated for the training and validation of the cross-species model (see “Methods” for details; Supplementary Data 3).

### Overview of MuSIC algorithm

**Fig. 2:**
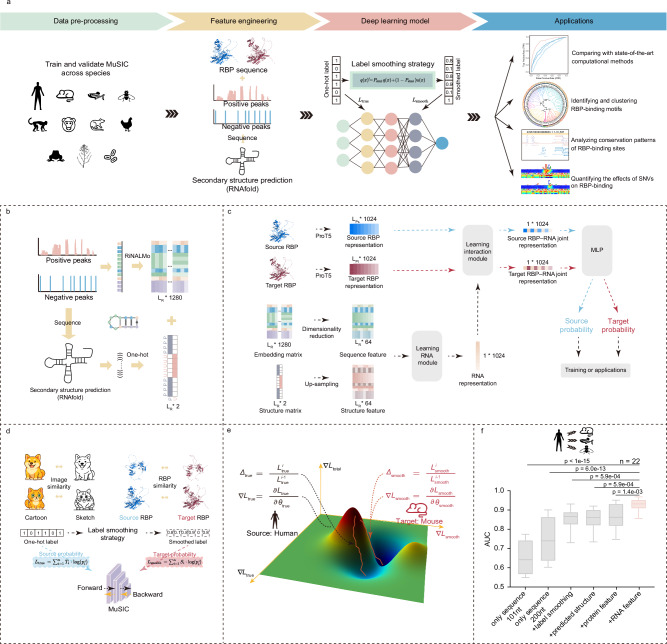
The workflow of MuSIC. **a** Schematic showing the workflow of MuSIC, including data pre-processing, feature engineering, model training, and downstream applications. **b** Flowchart showing data pre-processing and feature encoding. Top: positive (red) and negative (blue) peaks are encoded into embedding matrices using RiNALMo. Bottom: computationally predicted RNA structures are encoded as one-hot matrices. **c** Overview of the deep learning model architecture. RBP sequences are encoded into protein representations and RNA embedding and structural features are combined to generate RNA representations. RNA and RBP representations are then integrated to predict binding probabilities for the source and target species. **d** Diagram showing label smoothing strategy. Top left: using the image similarities between Cartoon and Sketch in computer vision for label smoothing strategy. Top right: using the RBP similarities between human and mouse for label smoothing. Bottom: transforming the one-hot labels into smoothed labels based on RBP conservation and computing cross-entropy loss for both one-hot labels and smoothed labels. **e** Diagram showing two components of the total loss ${L}_{{\mbox{total}}}$, ${L}_{{{{\rm{true}}}}}$ (human in black) and ${L}_{{{{\rm{smooth}}}}}$ (mouse in red). **f** Box plot showing the prediction performance for different types of input features, including sequence-only features (101nt or 200nt), 200nt sequence features with label smoothing, further incorporating predicted RNA structure features, protein features for 22 cross-species datasets (*n* = 22 RBP datasets; center line, median; box limits, 25th and 75th percentiles; whiskers, 1.5 × interquartile range; one-way repeated-measures ANOVA with Holm–Sidak multiple-comparisons test). Source data are provided as a Source Data file.

We developed MuSIC, a deep learning framework that could integrate RBP-binding peaks and cross-species conservation information of RBPs to accurately model and predict RBP–RNA interactions in diverse non-human species. Briefly, the MuSIC framework consists of four components (Fig. 2a): (i) pre-processing input data: collecting peaks of RBPs from multiple species, and constructing training and validation sets; (ii) feature engineering: incorporating RBP sequences from source and target species, nucleotide sequences and RNA secondary structures to learn RBP-binding preferences and enhance the prediction ability of the model (Fig. 2b); (iii) predicting RBP–RNA interactions**:** dynamic optimization on the prediction results using label smoothing regularization and gradient weight adaptation through convolutional and transformer-based neural network (Fig. 2c–e and Supplementary Fig. 3a, b); and (iv) analyzing RBP–RNA interactions: cross-species motif identification and other downstream applications.

We realized that the cross-species prediction tasks often have great challenges in generalization, e.g., the ability to accurately predict RBP–RNA interactions in mouse based on the same distribution as the training data in human^34,35^. There are several disadvantages in the existing deep learning-based methods, such as overfitting caused by the limitations of one-hot label loss functions, and heightened sensitivity to label noise and boundary ambiguities inherent in one-hot supervision^37^. To this end, we leveraged the label smoothing strategy that has been widely used in the field of computer vision to facilitate the cross-style classification^36^. In MuSIC, the conservation of RBPs across species can be used to enhance the model generalization and mitigate overfitting in a similar way. Briefly, MuSIC employs a approach in applying the label smoothing strategy, in which the one-hot labels were converted into soft labels based on the pre-defined RBP conservation scores^38^. MuSIC model combines the learning RNA module and learning interaction module to capture and enhance RBP–RNA interaction features learning. The learned RBP–RNA representations were then integrated using the multilayer perceptron (MLP) classifier to generate the prediction outputs (Fig. 2b, c and Supplementary Fig. 3a, b; see “Methods” for details). In addition, the cross-entropy loss was computed for both label types, with the final loss obtained by weighting and summing the two components (Fig. 2d). Finally, we used gradient weight adaptation algorithm to dynamically adjust the weight of each loss component during the model training, in which overfitting could be minimized by dropout and early stopping (Fig. 2e; see “Methods” for details).

Existing state-of-the-art computational methods typically utilize input sequence lengths of 101nt^28,30–32^ or 400nt^26,56^. We found that increasing the input sequence length to 200nt could significantly improve the Area Under the Curve (AUC) score, reaching 0.75 in cross-species prediction compared to sequences of lengths 101nt, 300nt, 400nt, 500nt, 1000nt, and 2000nt (Fig. 2f, Supplementary Fig. 3c and Supplementary Data 3). We also performed the same analysis in the within-species setting, and found that MuSIC could achieve the best prediction performance under the input length of 101nt, while 200nt showing a comparable prediction performance (Supplementary Fig. 3d). Given the primary objective of addressing the cross-species generalization issue, we selected 200nt as the default input sequence length in MuSIC. The inclusion of label smoothing, and gradient adaptation could further improve the AUC score from 0.75 to 0.84 (Fig. 2f). Notably, incorporating predicted RNA structures could further enhance the AUC score to 0.85 in cross-species prediction (Fig. 2f). We confirmed that using predicted RNA structures could have better prediction performance than using the randomized RNA structures (Supplementary Fig. 3e). Furthermore, we assessed the prediction performance using experimentally measured in vivo RNA structures instead of the predicted RNA structures (Supplementary Fig. 3e). We used the predicted RNA structures in MuSIC because of the limited performance gains by using the in vivo RNA structures, as well as very few in vivo RNA structure datasets are available in other species.

The protein and RNA foundation models trained on large-scale unlabeled datasets provide potential binding features for RBP and RNA^45–48^. We incorporated protein foundation model (ProtT5^48^) together with RNA foundation model (RiNALMo^46^) to extract high-dimensional representations for both RBPs and RNAs. Incorporating the ProtT5-derived RBP embeddings could improve the average AUC from 0.85 to 0.86. Furthermore, incorporating RiNALMo-derived RNA embeddings could lead to an additional improvement of average AUC from 0.86 to 0.91 (Fig. 2f; see “Methods' for details). Taken together, these results reveal that extending the sequence length, applying label smoothing and incorporating foundation models could significantly improve the performance of cross-species generalization.

We then characterized the contribution of each component of the MuSIC framework to the performance of feature learning using t-SNE and the Fréchet Inception Distance (FID), which measures the similarity of embeddings, with a higher value indicating better discriminability. The positive and negative samples of input RNA sequences cannot be clearly separated in the original feature space. After adding the SE and 1D residual block of the learning RNA module processing, the t-SNE clustering performance on the positive and negative samples could be significantly improved, thus leading to the apparent discriminative power in the RBP–RNA representation block of the learning interaction module (Supplementary Fig. 3f–h).

The classification performance of MuSIC’s frameworks was benchmarked against diverse transfer learning frameworks, foundation models, machine learning and deep learning classifiers using the identical input features. We found that MuSIC consistently outperformed all the benchmarks, confirming the robustness and effectiveness of MuSIC in modeling RBP binding across species (Supplementary Fig. 3i–l).

In addition, we computed the GC-content distributions for both positive and negative sequences across multiple cross-species datasets. The positive and negative samples showed comparable GC-content distribution, indicating that the GC-content cannot be the key confounding factor in either model training or evaluation (Supplementary Fig. 3m, n). Because cross-species prediction does not correspond to a traditional cross-validation setting, we further assessed the effect of different training–validation split ratios. As expected, MuSIC exhibited highly consistent performance across a wide range of split ratios (average AUC: 4:1 = 0.884, 2:1 = 0.885, 1:1 = 0.886, 1:2 = 0.885, 1:4 = 0.882), demonstrating that the cross-species prediction performance will not be influenced by the subsampling procedure (Supplementary Fig. 3o; see “Methods” for details).

### MuSIC outperforms existing computational methods in predicting cross-species RBP–RNA interactions

We evaluated the prediction performance of MuSIC using 274 datasets from 186 RBPs, benchmarked under both within-species (230 datasets from 186 RBPs) and cross-species (44 datasets from 17 RBPs) scenarios (Supplementary Data 4). All the datasets were processed using a uniform preprocessing pipeline to ensure fairness in the performance evaluation (see “Methods” for details). Briefly, we performed a systematic comparison of prediction accuracy with five existing state-of-the-art computational methods, including PaRPI^32^, HDRNet^31^, PrismNet^30^, DeepBind^28^, and GraphProt^26^. As expected, MuSIC showed better prediction accuracy than other methods for the within-species prediction (average AUC: MuSIC = 0.95, PaRPI = 0.91, HDRNet = 0.88, PrismNet = 0.86, DeepBind = 0.67, GraphProt = 0.72) (Supplementary Fig. 4a).

**Fig. 3:**
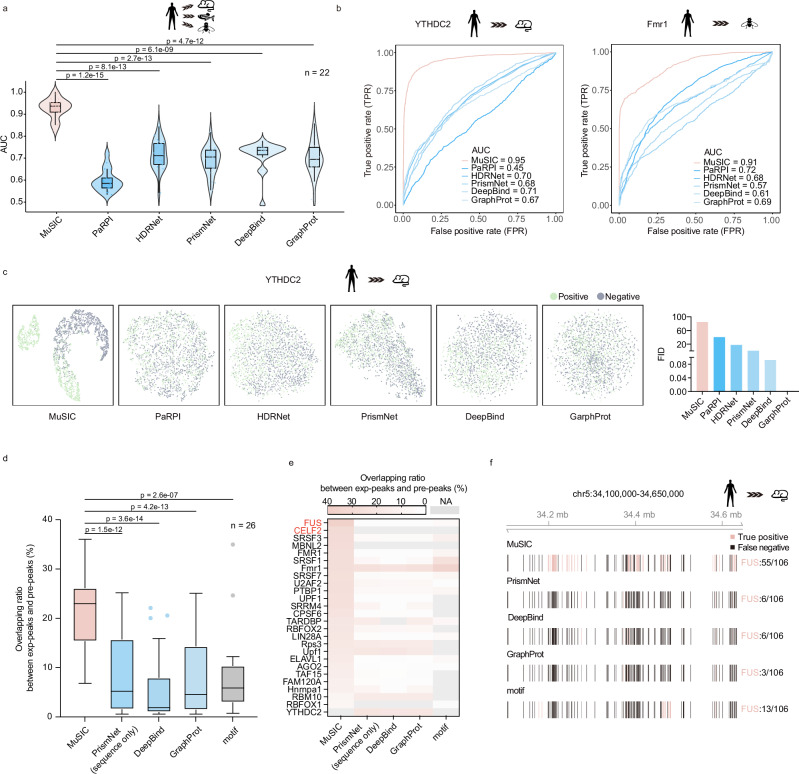
Comparison of prediction performance between MuSIC and other computational methods. **a** Violin plot showing the prediction accuracy of MuSIC and other computational methods for 22 cross-species datasets (*n* = 22 RBP datasets; center line, median; box limits, 25th and 75th percentiles; whiskers, 1.5 × interquartile range; two-sided paired Student’s t-test). **b** ROC curves showing the prediction accuracy of MuSIC and other computational methods for two RBPs, YTHDC2 (left) and Fmr1 (right). **c** Left: t-SNE clustering showing the output feature maps by MuSIC and other computational methods for separating the positive and negative peaks. Right: Bar plot showing the FID which assesses the distance between the positive and negative feature distributions learned by MuSIC and other computational methods for YTHDC2. **d** Box plot showing the overlapping ratio for 26 RBPs between the experimentally-derived and predicted peaks by MuSIC, other computational methods, and motif-based baseline (*n* = 26 RBPs; center line, median; box limits, 25th and 75th percentiles; whiskers, 1.5 × interquartile range; points beyond the whiskers, outliers; one-way repeated-measures ANOVA with Dunnett’s multiple-comparisons test). **e** Heatmap showing the overlapping ratio between the experimentally-derived and predicted peaks by MuSIC and other computational methods. **f** An example of predicted peaks distribution along the local region in mouse genome (chr5:34,100,000–34,650,000). Red indicates true positives and black indicates false negatives, as evaluated against the experimentally-derived peaks. Source data are provided as a Source Data file.

We then evaluated the prediction accuracy of MuSIC and other computational methods in the cross-species prediction across 44 datasets from human, mouse, zebrafish, and fly. MuSIC outperformed other methods, showing an obvious improvement of in AUC (average AUC: MuSIC = 0.91, PaRPI = 0.58, HDRNet = 0.72, PrismNet = 0.70, DeepBind = 0.68, GraphProt = 0.71; Fig. 3a), as well as in the accuracy (ACC) and Area Under the Precision-Recall Curve (AUPRC) metrics (Supplementary Fig. 4b–d). In addition, we found that MuSIC achieves high accuracy for predicting RBP–RNA interactions in both the closely related predictions (e.g., human to mouse) and distantly related predictions (e.g., human to zebrafish or fly) (Supplementary Fig. 4e, f). For example, MuSIC showed AUCs of 0.95 for YTHDC2 (mouse) and 0.91 for Fmr1 (fly) in the cross-species prediction, a significant improvement than other computational methods (Fig. 3b and Supplementary Fig. 5a). Taken together, these results showed that MuSIC could improve the prediction accuracy of RBP–RNA interactions than other computational methods, especially under the cross-species scenario. Consistently, MuSIC showed better performance in separating the positive and negative samples of input RNA sequences in the feature space than other computational methods (Fig. 3c and Supplementary Fig. 5b, c).

Finally, we compared the prediction accuracy of MuSIC and other computational methods by evaluating to what degree the RBP-binding peaks from CLIP-seq experiments could be rediscovered (see “Methods” for details). Briefly, the model was trained based on the RBP-binding peaks in human, followed by predicting peaks across all the transcripts in mouse (for 22 RBPs), zebrafish (for 1 RBP) and fly (for 3 RBPs) (Supplementary Data 4). For each RBP, we observed that MuSIC showed consistently better performance than other computational methods (Fig. 3d and Supplementary Fig. 5d), particularly on the cases of FUS and CELF2 (Fig. 3e). Notably, PaRPI and HDRNet were excluded in this analysis due to its reliance on the experimental RNA structure features, which were not available in the relevant datasets. For all the RBPs analyzed here, the predicted peaks by MuSIC showed high correlation with the experimentally derived ones (r = 0.78, Spearman’s rank correlation test; Supplementary Fig. 6a–c). Here, we showcased the site-level prediction performance of MuSIC using FUS within the local genomic region (chr5: 34,100,000–34,650,000) in mouse, where 55 out of 106 experimentally derived peaks could be successfully recovered by MuSIC (Fig. 3f). In contrast, only 3–13 peaks could be predicted by other computational methods. These findings confirm the feasibility and effectiveness of MuSIC in accurately capturing RBP–RNA interactions across species.

### MuSIC generates systematic RBP-binding predictions in diverse species

**Fig. 4:**
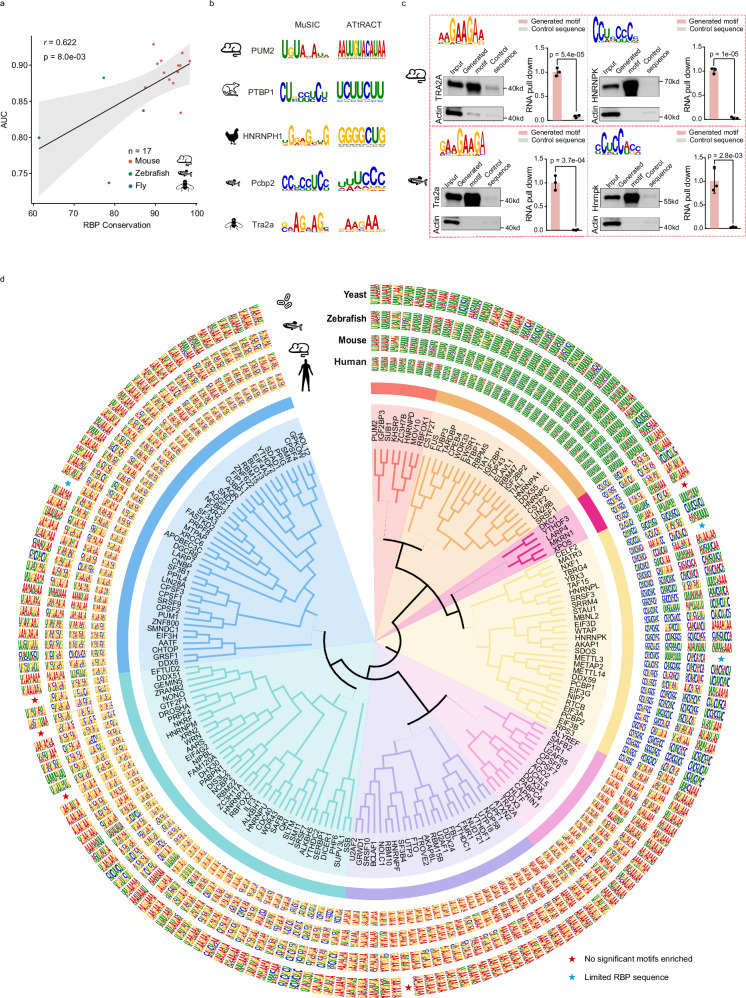
Reliable predictions of RBP-binding peaks and motif clustering by MuSIC. **a** Scatter plot showing the correlation between the cross-species prediction accuracy and RBP conservation for the 17 RBPs from mouse, zebrafish and fly (line, the fitted linear regression line; error bands, 95% confidence interval; Pearson’s correlation test). **b** Examples showing the consistency of the predicted binding motifs and experimentally-derived motifs. **c** In vitro RNA pull-down assays validating the predicted motifs for TRA2A and HNRNPK from mouse (top) and zebrafish (bottom), respectively. The predicted motifs, as well as the results of immunoblotting and RNA pull-down assays were shown. Data are presented as mean values +/- SD (*n* = 3 biologically independent experiments; two-sided unpaired Student’s t-test). **d** Hierarchical clustering of 184 predicted binding motifs from human, mouse, zebrafish, and yeast. Red stars indicate that there are no motifs enriched, and blue stars indicate RBPs with limited sequence availability. Source data are provided as a Source Data file.

The aforementioned results reveal that MuSIC could generate better cross-species predictions than other computational methods, particularly when the source and target species are more evolutionarily close. It will be useful to explore to what degree the source and target species are evolutionarily close, then the MuSIC can generate reliable prediction results. We then evaluated the relationship between the prediction accuracy and the RBP conservation between species in terms of both their sequence and structure (Supplementary Fig. 7a; see “Methods” for details). Notably, we observed a strong positive correlation (r = 0.622, Pearson’s correlation test; Fig. 4a) between the prediction accuracy (measured by AUC) and the RBP conservation based on 17 RBPs shared among mouse, zebrafish and fly, indicating that the degree of RBP conservation could be used as quantitative reference to threshold the prediction results of RBP–RNA interactions. We then predicted the RBP–RNA interactions for the 10 species in a broad evolutionary range using MuSIC, and filtered low-confidence prediction results based on the degree of RBP conservation equivalent to AUC of 0.75 (see “Methods” for details).

As expected, more than 85% of the RBPs showed high-confidence prediction results (i.e., with prediction AUC > 0.75) across a broad range of metazoan species, including orangutan, monkey, mouse, rat, chicken, frog, zebrafish and fly (Supplementary Fig. 7b). In contrast, only a small fraction (37.6%–55.9%) of the RBPs showed high-confidence prediction results in yeast and *A. thaliana* (Supplementary Fig. 7b). Taken together, these findings confirm that MuSIC performs significantly better in the metazoan species, by leveraging the RBP conservation patterns in terms of both sequence and structure (Supplementary Fig. 7b and Supplementary Data 5).

### MuSIC improves RBP-binding motif identification in diverse species

Previous studies have revealed that conserved RBPs tend to have conserved RBP-binding motifs during evolution, which has been validated by some specific RBPs^42,43^. However, this hypothesis has not been systematically tested in a broad set of RBPs. To this end, we first predicted the RBP-binding peaks across all the 11 species using the trained 2,018 RBP models, and identified the enriched RBP-binding motifs in the human and non-human species (Supplementary Data 5). The predicted enriched motifs in the non-human species could be successfully recapitulated by the well-known RBP binding sequence preference^53^. For example, the motif UGUANA for PUM2 and UUC for PTBP1 could be rediscovered in mouse and rat, respectively (Fig. 4b and Supplementary Fig. 7c).

Furthermore, we found that the inferred RBP-binding motifs with high confidence showed distinct degree of conservation during evolution (Supplementary Fig. 7d and Supplementary Data 5). For example, poly-GAA motifs for TRA2A and polyC motifs for HNRNPK were predicted in diverse species ranging from human to yeast. We then performed the RNA pull-down assays to experimentally validate these de novo motifs in mouse and zebrafish (Fig. 4c). These results confirmed the generalizability of MuSIC in identifying enriched binding preference for the RBPs in non-human species.

Finally, we visualized and clustered the RBP-binding motifs across all 11 species ranging from human to yeast based on their similarities (see “Methods” for details). Notably, RBP-binding motif prediction failed for certain RBPs due to the limited availability of protein sequences (e.g., fly: APOBEC3C and IGF2BP2; *A. thaliana*: SDOS; yeast: APOBEC3C, SDOS, and TBRG4), as well as insufficient motif enrichment (e.g., orangutan: HNRNPF; monkey: HNRNPF; fly: MBNL2 and HNRNPF; *A. thaliana*: HNRNPF and MOV10; yeast: XPO5, XRN2, NONO, DDX51, and HNRNPF) (Fig. 4d and Supplementary Fig. 8).

### MuSIC reveals evolutionarily conserved RBP-binding peaks

**Fig. 5:**
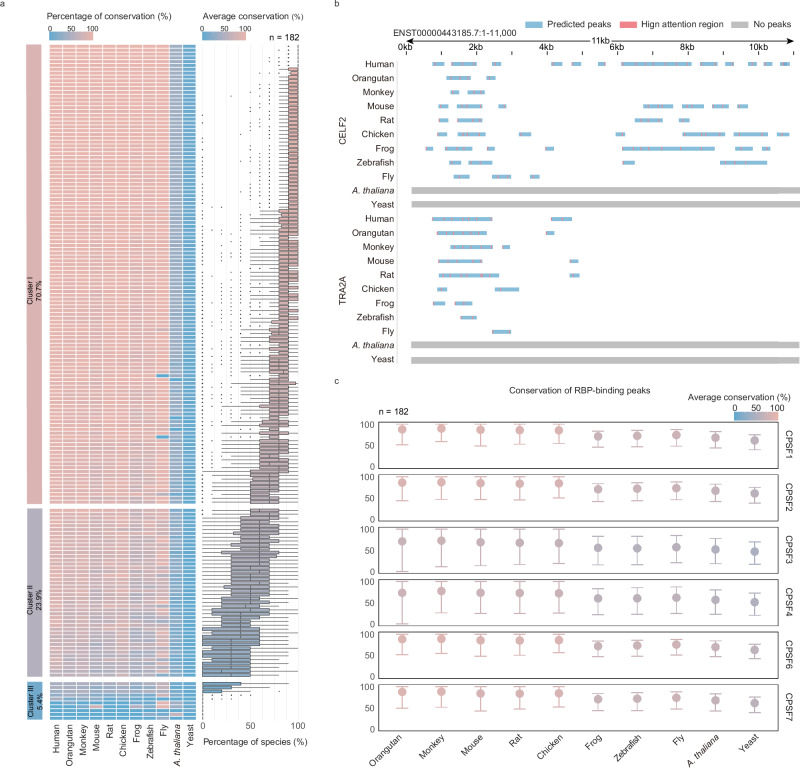
Cross-species conservation of RBP-binding peaks. **a** Heatmap showing the percentage of homologous transcripts containing the conserved peaks bound by RBPs across the 11 species, where all the 184 RBPs can be categorized into three groups. Box plot showing the conservation degree (measured by percentage of species) of the 182 homologous transcripts containing the conserved peaks (*n* = 182 transcripts; center line, median; box limits, 25th and 75th percentiles; whiskers, 1.5 × interquartile range; points beyond the whiskers, outliers). **b** Examples showing the predicted peaks bound by CELF2 and TRA2A along the transcript ENST00000443185.7. **c** Forest plot showing the conservation of the peaks bound by the CPSF family across the 182 homologous transcripts. Points indicate the mean of all values. Error bars indicate the mean of the lowest 25% and the mean of the highest 25% of the ranked values (*n* = 182 transcripts). We include the predicted peaks ranging from human to each of the non-human species shown here according to the phylogenetic tree. Source data are provided as a Source Data file.

It has been shown that the RBP-binding peaks from an RBP are either evolutionarily conserved or species-specific^44,57^. The conservation patterns of RBP-binding peaks could be influenced by both the RBP itself and the conservation of the target RNA sequence. To systematically evaluate the conservation of RBP-binding across species, we analyzed the predicted RBP-binding peaks of 184 RBPs across 11 species in the conserved transcripts (Supplementary Fig. 9a; see “Methods” for details). As expected, we observed higher proportion of conserved RBP-binding peaks in the more closely related species (Fig. 5a). Almost 71% of the total RBPs analyzed here exhibited considerable global patterns of RBP-binding peaks conservation (Fig. 5a). We highlighted two RBPs, CELF2 and TRA2A, with their RBP-binding peaks from 9 species on the evolutionarily conserved genomic region (Fig. 5b). Notably, there were fewer or none evolutionarily conserved RBP-binding peaks in *A. thaliana* and yeast.

We further investigated the conservation of RBP-binding peaks by analyzing the RBPs containing the same protein family from different species. The CPSF family RBPs and the IGF2BP family RBPs exhibited consistently strong RBP-binding peaks conservation across species (Supplementary Fig. 9b), consistent with previous studies reporting gradient conservation of the RBP-binding peaks^44^. Further exploration of the relationship between RBP-binding peaks conservation and species evolution revealed that, while CPSF family RBPs maintained the steady degree of RBP-binding peaks conservation of in mammals, an obviously decrease showed among the distantly related species (i.e., from frog; Fig. 5c). Similar patterns could be also observed in the IGF2BP family RBPs (Supplementary Fig. 9c). Taken together, these results indicate that the RNA-binding domains may play critical roles in maintaining the conserved RBP-binding peaks across species.

### MuSIC prioritizes genetic variation with consistent effects influencing RBP-binding across species

Genetic variations, such as SNVs, can potentially alter RBP-binding affinity by disrupting RNA target recognition, and thus cause functional abnormalities and severe diseases^8,58,59^. The ability of predicting RBP-binding peaks across species by MuSIC enables us to quantify and analyze the effects of SNVs on RBP binding in the homologous regions between human and non-human species, which will be valuable for leveraging model organisms such as mouse to interpret the RBP-mediated mechanisms of human disease-associated SNVs^10,60^.

**Fig. 6:**
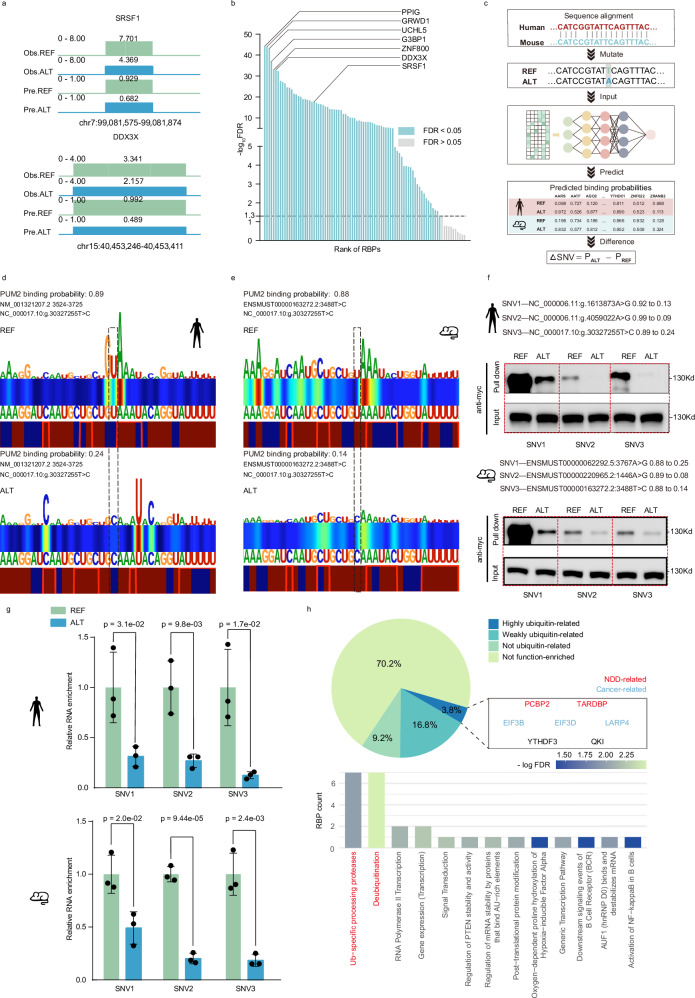
RBP–RNA interactions affected by SNVs and their associations with human diseases. **a** The experimentally-derived and predicted effects of SNVs on SRSF1 and DDX3X binding. Obs.REF, the binding strength from CLIP-seq data for the reference allele; Obs.ALT, the binding strength from CLIP-seq data for the alternative allele; Pre.REF, the binding strength from MuSIC prediction for the reference allele; and Pre.ALT, the binding strength from MuSIC prediction for the alternative allele. **b** Consistency between the experimentally-derived and predicted effects of SNVs on RBP binding for the 92 RBPs. Green bar indicates high consistency (-logFDR ≥ 1.3), whereas grey bar indicates no significance. **c** Schematic showing the workflow for quantifying the effects of homologous SNVs on RBP binding. **d** Example showing the effect of a homologous SNV (NC_000017.10:g.30327255 T > C) on PUM2 binding in human. The saliency maps show the predicted effect of REF (U) (top) and ALT (C) (bottom) on PUM2 binding. The homologous SNV position is highlighted by a dashed box. **e** Example showing the effect of a homologous SNV (ENSMUST00000163272.2:3488 T > C) on PUM2 binding in mouse. The saliency maps show the predicted effect of REF (U) (top) and ALT (C) (bottom) on PUM2 binding. **f** Examples showing in vitro RNA pull-down assays (*n* = 3 biologically independent experiments) examining the effects of three homologous SNVs on PUM2 binding in human (top) and mouse (bottom). **g** Bar plots showing in vivo POND-qPCR quantification of REF/ALT target RNAs binding to human and mouse PUM2 in HEK293T. Green bar represents RNA enrichment for REF sequences, and blue bar for ALT sequences. Data are presented as mean values +/- SD (*n* = 3 biologically independent experiments; two-sided unpaired Student’s t-test). **h** Enriched biological functions of the SNV-affected transcripts. In total, 7 RBPs are identified as highly ubiquitin-related, some of which have been revealed as associated with neurodegenerative diseases (red) and cancer (blue). Source data are provided as a Source Data file.

We first quantified and compared the binding affinity on the local regions with (i.e., alternative allele) and without (i.e., reference allele) SNVs using the eCLIP data^61^ from 103 RBPs (Supplementary Fig. 10a; see “Methods” for details). We found that SNVs detected in CLIP data were relevant to modulating RBP-binding affinity (Supplementary Fig. 10a and Supplementary Data 6). Inspired by these findings, we further evaluated the consistency between MuSIC predictions and experimental data for SNV effects. We found that MuSIC could accurately predict the RBP-binding affinity on the local region containing the reference and alternative alleles, which was exemplified with the cases of SRSF1 and DDX3X (Fig. 6a, b and Supplementary Fig. 10b; see “Methods” for details).

Furthermore, we performed systematic analyses on the SNVs from ClinVar database^7^ using MuSIC to quantify their disruption effects on RBP–RNA binding affinity (Supplementary Fig. 10c; see “Methods” for details). Interestingly, we found that the pathogenic SNVs exhibited significantly larger disruption effects on RBP binding compared with the benign ones (Supplementary Fig. 10d). For example, the c.−103C > T mutation in the 5′ UTR of GJB1 has been revealed to disrupt an internal ribosomal entry site (IRES) and impair GJB1 translation, which is associated with the development of X-linked Charcot–Marie–Tooth neuropathy^62,63^. These results indicate that the pathogenic SNVs could be implicated in the pathogenesis of diverse disease by altering RBP binding and thereby perturbing downstream post-transcriptional regulatory processes.

Next, we ask whether MuSIC could be applied in quantifying the effects of SNVs in the homologous regions between species (Fig. 6c; see “Methods” for details). By applying the analysis framework, we systematically screened the 32,207 synonymous SNVs in human genome, corresponding to 85,599 SNVs in the homologous regions in mouse genome. We predicted 59,811 SNVs in mouse genome that could potentially disrupt RBP binding (i.e., ∆SNV < −0.5) (Fig. 6c). Here, we showed an example: the SNV NC_000017.10: g.30327255 T > C in the human transcript NM_001321207.2 was predicted as disrupting PUM2 binding (∆SNV = −0.65) (Fig. 6d), consistent with the predicted effect of the SNV located in the homologous mouse transcript ENSMUST00000163272.2 (∆SNV = −0.74) (Fig. 6e). Furthermore, we performed in vitro RNA pull-down and in vivo POND-qPCR^64^ assays to validate the predictions by MuSIC. The POND (Protein nanocage-empOwered Non-Destructive) method is a recently developed technique for in vivo detection of RBP–RNA interactions, which enables selective packaging of RBP–RNA complexes into extracellular nanovesicles^64^ (Supplementary Fig. 11a). We focused on three SNVs predicted by MuSIC showing disrupted binding affinity by PUM2 in both human and mouse (Supplementary Data 7). In vitro RNA pull-down assays confirmed that the alternative alleles significantly altered PUM2 binding to the corresponding target RNA sequences in both species (Fig. 6f and Supplementary Fig. 11b, c). Consistently, in vivo POND-qPCR assays further supported these findings (Fig. 6g). Together, these results demonstrate that MuSIC can generate accurate and reliable prediction on the effects of homologous SNVs on RBP binding across species.

### Pervasive influenced RBP-binding implicated in disease mechanisms

Among all the 184 RBPs analyzed for their binding affinity changes on the SNVs in mouse genome, 38 (20.6%) RBPs were found to be enriched with the biological processes associated with ubiquitination regulation and protein degradation (Fig. 6h, Supplementary Fig. 12a, b and Supplementary Data 8). Previous studies have revealed that ubiquitination-related processes are implicated in the pathogenesis of complex diseases, such as neurodegenerative diseases^65,66^ and cancer^67–69^, which can be also validated by the predictions by MuSIC (Supplementary Fig. 12c). These findings suggest the critical roles of SNVs in modulating RBP binding and influencing the ubiquitination-associated processes, which may be implicated in complex disease mechanisms through disrupting protein homeostasis.

In our analysis, TARDBP was prioritized as highly related to the ubiquitination-related processes. TARDBP has been reported to be critical in regulating protein homeostasis and protein folding, and abnormal TARDBP binding on the target RNA transcripts can lead to several diseases in human and mouse^70–74^. We confirmed that the SNV in mouse could disrupt TARDBP binding to *foxp4*, a gene involved in ubiquitin metabolism (Supplementary Fig. 13a). Moreover, the TARDBP-bound genes perturbed by SNVs were significantly enriched in the functions related to development (Supplementary Fig. 13b–d).

## Discussion

Existing computational methods for predicting RBP–RNA interactions^26,28,30,31^ were generally developed for the within-species tasks, having difficulties in predicting cross-species. In this study, we developed MuSIC, a computational method that can accurately predict RBP-binding peaks in the species lacking available CLIP datasets by leveraging the multi-level conservation patterns of the RBP. Compared to other similar computational methods, MuSIC showed superior performance in predicting RBP-binding peaks on the cross-species tasks. We then systematically predicted RBP-binding peaks and their enriched sequence motifs for 184 RBPs in human and 10 non-human species using MuSIC. We confirmed that the conservation patterns of the predicted RBP-binding peaks were consistent with previous studies^43,44,57^. Finally, we applied MuSIC to investigate the effects of SNVs on RBP binding, particularly focusing on the imbalance in RBP binding induced by SNVs and exploring their potential connections to diseases such as cancer and neurodegenerative disorders.

The following improvements in the model architecture are critical for the augmented prediction performance of MuSIC in the cross-species tasks: optimization of input feature length, label smoothing and integrating foundation models. Previous studies have showed that increasing input sequence length can dramatically improve the model performance^24,75^. In our study, we revealed that increasing the input sequence length from 101nt to 200nt could significantly improve the prediction performance across species (AUC from 0.65 to 0.75). In contrast, further increasing the sequence length beyond 200nt, including 300nt, 400nt, 500nt, 1000nt, and 2000nt, led to a consistent decline in model performance. Increasing the input sequence length can not only provide more useful sequence information, but also may include noisy and redundant sequence information for modeling RBP binding. Inspired by the concepts from computer vision, we implemented label smoothing in the model to further improve prediction performance^36–38^. We showed that label smoothing and the gradient weight adaptation mechanism could significantly improve cross-species predictions. This strategy reflects the inherently dynamic nature of biomolecular recognition, which operates on a continuum rather than a binary decision framework^76^. Recent advances in protein and RNA foundation models have enabled the transfer of rich contextual and evolutionary information, improving RBP binding predictions for diverse RBPs from different cell lines and species^31,32,46–48,77^. Consistent with this trend, we showed that integrating protein and RNA foundation models substantially improves cross-species RBP–RNA interaction prediction, highlighting the complementary contributions of foundation models. Importantly, the integration of conservation-aware label smoothing and foundation model representations for RBP and RNA enables MuSIC to more accurately identify dynamic RBP–RNA interactions across species.

Previous studies revealed that genetic variation in RNA can potentially reduce RBP-binding affinity, thereby disrupting downstream post-transcriptional regulatory processes associated with the development of diseases^8,58,62,63,78^. However, the effects of SNVs on RBP binding has not fully explored, particularly in the non-model organisms. In our study, we quantified the effects of SNVs on RBP binding in the homologous regions between human and mouse, and furthermore experimentally validated these prediction results in vitro and in vivo. Our results revealed the evolutionary conservation of genetic variation influencing RBP binding across species, and found that the SNVs that could potentially alter RBP–RNA interactions are associated with human diseases^9,10^. We then performed a comprehensive analysis on the homologous human SNVs in mouse to quantify their binding affinity changes for each of the 184 RBPs. Interestingly, the disrupted binding events generally are recognized by TARDBP, and the target transcripts are enriched with the ubiquitination-associated pathways^31^. The dysregulation of ubiquitin–proteasome system (UPS) can lead to protein misfolding and accumulation, which has been linked to human diseases including cancer and neurodegenerative disorders^79,80^.

In summary, we propose MuSIC as a model to predict RBP-binding peaks across species. MuSIC outperformed state-of-the-art prediction models, and generated a catalog of predicted RBP-binding peaks of 184 RBPs with high accuracy in 11 species. The detailed analysis quantified the effects of genetic variants on RBP binding, particularly highlighted in the homologous regions between human and mouse. MuSIC provides insights into the RBP–RNA interactions in non-model organisms, and generates hypotheses on the interplays between RBP-mediated regulation and disease mechanisms.

## Methods

### Compiling RBP-binding peak datasets

We obtained the RBP-binding peaks of 216 RBPs from POSTAR3^33^ and 29 RBPs from the modENCODE project^55^ for the within- and cross-species model training and testing. We applied a uniform preprocessing pipeline to the peak datasets to minimize putative bias in the datasets. Briefly, we first removed the duplicated and short (length <5nt) peaks in each dataset. Then, we selected the top 5000 peaks with the highest binding strength in each dataset for the downstream analysis. Next, the peaks were extended by 200nt on both sides. We used GFF Annotation Parser (https://github.com/lipan6461188/GAP) to extract the RNA sequences of these extended peaks, which were considered as the positive peaks (i.e., RBP-binding peaks) in the model training and validation. We randomly selected 5000 RNA sequences of 200nt in length from the remaining genomics regions as the negative ones.

For the within-species prediction, 230 datasets of 186 RBPs from human, mouse, zebrafish and fly were used. For the cross-species prediction, 34 datasets of 13 RBPs shared between human and mouse, 4 datasets of one RBP shared between human and zebrafish and 6 datasets of 3 RBP shared between human and fly were used. Notably, the datasets of the same RBP generated by different CLIP-seq technologies were excluded in the cross-species analysis. For the within-species prediction, we used randomly 80% of the positive and negative peaks for the model training, whereas 20% were used for the model validation.

We note that cross-species prediction does not correspond to a traditional cross-validation setting, as training and testing are performed on entirely distinct species. To assess whether the choice of split ratio between training and validation affects evaluation results, we systematically examined different split ratios in the cross-species setting, including 4:1, 2:1, 1:1, 1:2, and 1:4. For instance, when setting the split ratio as 4:1, the training set contains 10,000 sequences while the corresponding validation set contains 2500 sequences. Thus, the different ratios correspond to validation set sizes of 2500; 5000; 10,000; 20,000 and 40,000 sequences, respectively.

For target-species datasets that were insufficient to support certain split ratios, we evaluated robustness through repeated subsampling using three different random seeds, i.e., 40, 50 and 60. For each random seed, 66% of the available target-species data was randomly selected as the validation dataset. And the reported AUC score for these RBPs represents the average performance across the AUC obtained from the three different random seeds. Finally, the training-to-validation ratio of 4:1 was used for model training.

### Reconstructing phylogenetic tree among the 11 species

We aim to develop a computational method to systematically predict RBP–RNA interactions in 11 species, including *Homo sapiens* (human), *Pongo abelii* (orangutan), *Macaca fascicularis* (monkey), *Mus musculus*(mouse), *Rattus norvegicus* (rat), *Gallus gallus* (chicken), *Xenopus laevis* (frog), *Danio rerio* (zebrafish), *Drosophila melanogaster* (fly), *Arabidopsis thaliana* (*A. thaliana*), and *Saccharomyces cerevisiae* (yeast).

We retrieved the 18S rRNA sequences of the 11 species from RNAcentral^81^ (Release 24) for evaluating the evolutionary distance among these species. We then performed multiple sequence alignment of the 18S rRNA sequences using the Sequence Alignment tool in MEGA^82^ v11.0.13, followed by constructing a phylogenetic tree among the 11 species using the neighbor-joining method. We then evaluated the reliability of the reconstructed phylogenetic tree using the Bootstrap test with 1000 replicates.

### Compiling sequence and structural datasets of RBPs

We collected the protein IDs of 216 RBPs^33^ that were used in this study in the 11 species from UniProt^83^ (Supplementary Data 1). For each RBP, the corresponding sequences and structural data were retrieved from UniProt^83^. In the cases where predicted structural data for certain RBPs were unavailable in UniProt, we used AlphaFold2^84^ to predict and supplement the missing ones. The sequence similarity of the full-length RBPs between human and other species was computed using ClustalX^85^ v2.1.1. We used US-align^86^ to evaluate the structural similarity of the full-length RBPs by calculating two metrics: TM-score (overall structure similarity) and pLDDT (confidence of predicted structure).

We obtained the annotations of 130 RBDs in the 11 species from InterPro^87^ (Release 103.0), including their sequence positions and respective fragments in their full-length RBPs. The local structures of RBDs were extracted from the structures of full-length RBPs, ensuring the local structures of RBDs were aligned with the structures of full-length RBPs. Finally, ClustalX^85^ v2.1.1 and US-align^86^ were used to calculate the sequence and structural similarity of the RBDs between human and other species.

### Comparing RBP–RNA interactions in three-dimensional structures between species

We collected three-dimensional structures of homologous RBP RC3H1 (PDB ID 4QIL in human, PDB ID 4QI2 in mouse) and their bound RNA fragments from the PDB^88^. In addition, we used AlphaFold3^54^ webserver to predict the interactions between homologous RBP IGF2BP1 and bound RNA fragments in human and mouse based on the IGF2BP1 protein sequence in human and mouse, as well as well-known RNA sequence with strong preference by IGF2BP1 (5’-UGACUUACUUCAUUAUUGAA-3’). The cartoon and surface diagrams displaying the bound RNA, RBDs, and the residues involved in the interactions were generated using PyMOL v3.1.0 (http://www.pymol.org/pymol).

### Pre-processing MuSIC input data

We used the protein foundation model ProtT5^48^ to extract the RBP sequence of source and target species features into embedding matrix of size (${L}_{{{{\rm{P}}}}{{{\rm{s}}}}}$, 1024) and (${L}_{{{{\rm{P}}}}{{{\rm{t}}}}}$, 1024). The RNA sequence of each peak in the datasets was extracted into the embedding matrix of size (200, 1280) using pre-trained RNA foundation model RiNALMo^46^. The secondary structure of each RNA sequence was predicted using RNAfold^89^ v2.1.0 with default settings. The resulting structure was encoded into a 2-dimensional matrix of size (200, 2), with ‘P’ and ‘U’ representing the paired and unpaired bases, respectively.

### Overview of MuSIC architecture

The RBP feature for MuSIC are defined as **P**. And the input data of RNA for MuSIC is defined as **r**, which contains *N* peaks. The sequence and structure features are represented as ${{{{\rm{r}}}}}_{{{{\rm{seq}}}}}\in {{{{\rm{R}}}}}^{{N\times L}_{r}\times 1280}$ and ${{{{\rm{r}}}}}_{{{{\rm{str}}}}}\in {{{{\rm{R}}}}}^{{N\times L}_{r}\times 2}$ where ${L}_{r}$ denotes the sequence length. After dimensionality reduction of ${{{{\rm{r}}}}}_{{{{\rm{seq}}}}}$ and feature up-sampling of ${{{{\rm{r}}}}}_{{{{\rm{str}}}}}$ using linear layers, two features are concatenated to form the RNA features ${{{\rm{R}}}}\in {{{{\rm{R}}}}}^{{N\times L}_{r}\times 128}$.

These features are used as inputs to MuSIC, and the binding probability ${{{\rm{Y}}}}\in {{{{\rm{R}}}}}^{N}$ is computed by as follows:

1 $${{{\rm{Y}}}}={{\mbox{MuSIC}}}\left({{{\rm{R}}}},{{{\rm{P}}}}\right)$$

MuSIC is defined as follows:

2 $${{\mbox{MuSIC}}}\left({{{\rm{R}}}},{{{\rm{P}}}}\right)={{{\rm{MLP}}}}\left({{{\rm{Li}}}}\left({{{\rm{Lr}}}}\left({{{\rm{R}}}}\right),{{{\rm{P}}}}\right)\right)$$

where ${{{\rm{MLP}}}}$ is the MLP module, ${{{\rm{Li}}}}$ is the learning interaction module and ${{{\rm{Lr}}}}$ is the learning RNA module, respectively.

### Overview of learning RNA module

The learning RNA module is defined as follows:

3 $${{{\rm{Lr}}}}\left({{{\rm{R}}}}\right)={{{\rm{FC}}}}\left({{{{\rm{f}}}}}_{{{{\rm{R}}}}}\left({{{{\rm{f}}}}}_{{{{\rm{S}}}}}\left({{{{\rm{f}}}}}_{{{{\rm{C}}}}}\left({{{\rm{R}}}}\right)\right)\right)\right)$$

where FC is the fully connected layer, ${{{{\rm{f}}}}}_{{{{\rm{R}}}}}$ is the residual blocks, f_S_ is the squeeze-excitation block (SE), and ${{{{\rm{f}}}}}_{{{{\rm{C}}}}}$ is the convolutional block, respectively.

#### Convolutional block

First, we use a convolutional layer to extract features. The convolutional block ${{{{\rm{f}}}}}_{{{{\rm{C}}}}}\left({{{\rm{R}}}}\right)$ is defined as follows:

4 $${{{{\rm{f}}}}}_{{{{\rm{C}}}}}\left({{{\rm{R}}}}\right)={{{\mbox{ReLU}}}}\left({{{\mbox{BN}}}}\left({{{\mbox{Conv}}}}_{K,P}\left({{{\rm{R}}}}\right)\right)\right)$$

where ${{{\mbox{Conv}}}}_{K,P}\left({{{\rm{R}}}}\right)$ is a 2D convolutional layer with kernel $K$ and padding $P$. The convolutional layer is designed to extract local sequence and structural features from the input data.

#### Squeeze-and-excitation (SE) block

We apply a SE block to recalibrate channel-wise feature responses. The SE block is defined as follows:

5 $${{{{\rm{f}}}}}_{{{{\rm{S}}}}}\left({{{\rm{X}}}}\right)={{{\rm{X}}}}\otimes {{\mbox{SE}}}\left({{{\rm{X}}}}\right)$$

6 $${{\mbox{SE}}}\left({{{\rm{X}}}}\right)={{{\rm{\sigma }}}}\left({{{{\rm{f}}}}}_{{{\mbox{ex}}}}\left({{{{\rm{f}}}}}_{{{{\rm{sq}}}}}\left({{{\rm{X}}}}\right)\right)\right)$$

where ${{{\rm{\sigma }}}}$ is the activation function, ${{{{\rm{f}}}}}_{{{{\rm{sq}}}}}$ is a global average pooling function to squeezes the global sequence and structural features into a channel statistic and ${{{{\rm{f}}}}}_{{{\mbox{ex}}}}$ applies a nonlinear transformation to the squeezed feature map. The SE block helps the model focus on the most relevant features for RBP binding.

#### Residual blocks

We then use residual blocks to capture both sequence and structural features over long ranges. These residual blocks are designed to learn the hierarchical features by passing information through several convolutional layers. Two types of residual blocks are used here:

ResidualBlock2D, which operates on 2D feature maps, is defined as follows:

7 $${{{{\rm{f}}}}}_{{{{\rm{R}}}}2}\left({{{\rm{X}}}}\right)={{\mbox{ResidualBlock}}}2{{\mbox{D}}}\left({{\mbox{Conv}}}\left({{\mbox{SE}}}\left({{{\rm{X}}}}\right)\right)\right)$$

ResidualBlock1D, which operates on 1D feature maps, is defined as follows:

8 $${{{{\rm{f}}}}}_{{{{\rm{R}}}}1}\left({{{\rm{X}}}}\right)={{\mbox{ResidualBlock}}}1{{\mbox{D}}}\left({{\mbox{AvgPool}}}\left({{{\rm{X}}}}\right)\right)$$

#### Fully connected layer

Finally, we apply a fully connected layer to generate the RNA representations for each RNA input feature, which is defined as follows:

9 $${{{{\rm{R}}}}}_{{{{\rm{rep}}}}}={{{{\rm{f}}}}}_{{{\mbox{FC}}}}\left({{{\rm{X}}}}\right)$$

### Overview of learning interaction module

In order to achieve information interaction between RNA representations and protein representations, inspired by the multi-head cross-attention mechanism, we adopt a similar approach to enable explicit information exchange. Formally, the definition of the learning interaction module is as follows:

10 $${{{\rm{Li}}}}\left({{{{\rm{R}}}}}_{{{{\rm{rep}}}}},{{{{\rm{P}}}}}_{{{{\rm{rep}}}}}\right)=\,{{{\rm{LN}}}}\left({{{{\rm{R}}}}}_{{{{\rm{rep}}}}}+{{{\rm{FFN}}}}\left({{{\rm{LN}}}}\left({{{{\rm{R}}}}}_{{{{\rm{rep}}}}}+{{{\rm{Attention}}}}\left({{{{\rm{R}}}}}_{{{{\rm{rep}}}}},{{{{\rm{P}}}}}_{{{{\rm{rep}}}}},{{{{\bf{P}}}}}_{{{{\rm{rep}}}}}\right)\right)\right)\right)$$

11 $${{{\mbox{Attention}}}}\left({{{{\bf{R}}}}}_{{{{\rm{rep}}}}},{{{{\bf{P}}}}}_{{{{\rm{rep}}}}},{{{{\bf{P}}}}}_{{{{\rm{rep}}}}}\right)={{{\mbox{MultiHeadAttention}}}}\left({{{\bf{Q}}}}=\,{{{{\bf{R}}}}}_{{{{\rm{rep}}}}},{{{\bf{K}}}}\,={{{{\bf{P}}}}}_{{{{\rm{rep}}}}},{{{\bf{V}}}}\,={{{{\bf{P}}}}}_{{{{\rm{rep}}}}}\right)$$

12 $${{{\rm{FFN}}}}\left({{{\bf{x}}}}\right)={{{{\bf{W}}}}}_{{{{\bf{2}}}}}{{{\rm{GELU}}}}\left({{{{\bf{W}}}}}_{{{{\bf{1}}}}}{{{\bf{x}}}}+{{{{\bf{b}}}}}_{{{{\mathbf{1}}}}}\right)+{{{{\bf{b}}}}}_{{{{\mathbf{2}}}}}$$

where ${{{{\bf{R}}}}}_{{{{\rm{rep}}}}}$ and ${{{{\bf{P}}}}}_{{{{\rm{rep}}}}}$ denote the RNA and protein representations, respectively. ${{\mbox{Attention}}}$ is the multi-head cross-attention mechanism, ${{{\rm{LN}}}}$ is the layer normalization and ${{{\rm{FFN}}}}$ is a position-wise feed-forward network consisting of two linear transformations with a ${{{\rm{GELU}}}}$ activation.

### Overview of MLP module

Finally, the MLP module processes the RBP–RNA joint representations from the learning interaction module through a fully connected neural network and generates the final output prediction.

13 $${{{\rm{MLP}}}}\left({{{\bf{x}}}}\right)=\,{{{{\bf{W}}}}}^{\left(L\right)}{{{\rm{ReLU}}}}\left({{{{\bf{W}}}}}^{\left(L-1\right)}{{{\rm{ReLU}}}}\left(\cdots {{{\rm{ReLU}}}}\left({{{{\bf{W}}}}}^{\left(0\right)}{{{\bf{x}}}}+{{{{\bf{b}}}}}^{\left(0\right)}\right)\cdots \right)+{{{{\bf{b}}}}}^{\left(L-1\right)}\right)+{{{{\bf{b}}}}}^{\left(L\right)}$$

where ${{{{\bf{W}}}}}^{\left(L\right)}$ and ${{{{\bf{b}}}}}^{\left(L\right)}$ represent the learnable weights and biases for layer $L$, ${{{\bf{x}}}}$ represents the refined RBP–RNA joint representations.

### Label smoothing strategy and gradient weight adaptation

We applied the label smoothing strategy to smooth the one-hot label distribution. Given a one-hot label distribution $q\left(x\right)$, the smoothed label distribution $q{{\hbox{'}}}\left(x\right)$ is defined as follows:

14 $$q{{\hbox{'}}}\left(x\right)=\alpha q\left(x\right)+\left(1-\alpha \right)u\left(x\right)$$

where $\alpha$ is the RBP conservation score as a smoothing coefficient, and $u\left(x\right)$ is the uniform distribution.

The one-hot label distribution $q\left(x\right)$ and a predicted binding probability distribution ${p}^{S}\left(x\right)$ from source RBP are used for computing the classification loss as follows:

15 $${L}_{{{\rm{true}}}}{{=}}{{\mathscr{H}}}\left(q\left(x\right),{p}^{S}\left(x\right)\right)=-{\sum }_{i=1}^{C}q\left({x}_{i}\right)\log \left({p}^{S}\left({x}_{i}\right)\right)$$

The smoothed label distribution $q\hbox{'}\left(x\right)$ and a predicted binding probability distribution ${p}^{T}\left(x\right)$ from target RBP are used for computing the smoothing loss as follows:

16 $${L}_{{{\rm{smooth}}}}{{=}}{{\mathscr{H}}}\left(q{{\hbox{'}}}\left(x\right),{p}^{T}\left(x\right)\right)=-{\sum }_{i=1}^{C}q{{\hbox{'}}}\left(x\right)\log \left({p}^{T}\left({x}_{i}\right)\right)$$

17 $${L}_{{{\rm{smooth}}}}{{=}}{{\mathscr{H}}}\left(q{{\hbox{'}}}\left(x\right),{p}^{T}\left(x\right)\right)=-{\sum }_{i=1}^{C}\alpha q\left({x}_{i}\right)\log \left({p}^{T}\left(x\right)\right)-{\sum }_{i=1}^{C}\left(1-\alpha \right)u\left({x}_{i}\right)\log \left({p}^{T}\left(x\right)\right)$$

The first term, *L*_true_, represents the typical classification loss, while the second term, *L*_smooth_, is the smoothing loss. Finally, the total loss is defined as the weighted combination of classification loss and smoothing loss as follows:

18 $${L}_{{{{\rm{total}}}}}=\alpha {L}_{{{{\rm{true}}}}}+\beta {L}_{{{{\rm{smooth}}}}}$$

We proposed gradient weight adaptation to address the conflict between the gradient directions of ${L}_{{{{\rm{true}}}}}$ and ${L}_{{{{\rm{smooth}}}}}$. First, the gradients of both losses are computed with respect to the feature encoder parameters and classifier parameters as follows:

19 $$\nabla {L}_{{{{\rm{true}}}}\left({{\mbox{encoder}}}\right)}=\frac{\partial {L}_{{{{\rm{true}}}}}}{\partial {\theta }_{{{\mbox{encoder}}}}},\,\nabla {L}_{{{{\rm{smooth}}}}\left({{\mbox{encoder}}}\right)}=\frac{\partial {L}_{{{{\rm{smooth}}}}}}{\partial {\theta }_{{{\mbox{encoder}}}}}$$

20 $$\nabla {L}_{{{{\rm{true}}}}\left({{{\rm{classifier}}}}\right)}=\frac{\partial {L}_{{{{\rm{true}}}}}}{\partial {\theta }_{{{{\rm{classifier}}}}}},\,\nabla {L}_{{{{\rm{smooth}}}}\left({{{\rm{classifier}}}}\right)}=\frac{\partial {L}_{{{{\rm{smooth}}}}}}{\partial {\theta }_{{{{\rm{classifier}}}}}}$$

The weight of each loss term can be dynamically adjusted based on the ratio of the current loss to the previous one as follows:

21 $${\Delta }_{{{{\rm{true}}}}}=\frac{{L}_{{{{\rm{true}}}}}^{i}}{{L}_{{{{\rm{true}}}}}^{i-1}},{\Delta }_{{{{\rm{smooth}}}}}=\frac{{L}_{{{{\rm{smooth}}}}}^{i}}{{L}_{{{{\rm{smooth}}}}}^{i-1}}$$

22 $${w}_{{{{\rm{true}}}}}=\frac{1}{{\Delta }_{{{{\rm{true}}}}}},{w}_{{{{\rm{smooth}}}}}=\frac{1}{{\Delta }_{{{{\rm{smooth}}}}}}$$

23 $${w}_{{{{\rm{true}}}}}=\frac{{w}_{{{{\rm{true}}}}}}{{w}_{{{{\rm{smooth}}}}}+{w}_{{{{\rm{true}}}}}},{w}_{{{{\rm{smooth}}}}}=\frac{{w}_{{{{\rm{smooth}}}}}}{{w}_{{{{\rm{smooth}}}}}+{w}_{{{{\rm{true}}}}}}$$

Subsequently, the gradient can be expressed as follows:

24 $$\nabla {L}_{{{\mbox{total}}}\left({{\mbox{encoder}}}\right)}={w}_{{{{\rm{true}}}}}\nabla {L}_{{{{\rm{true}}}}\left({{\mbox{encoder}}}\right)}+{w}_{{{{\rm{smooth}}}}}\nabla {L}_{{{{\rm{smooth}}}}\left({{\mbox{encoder}}}\right)}$$

25 $$\nabla {L}_{{{\mbox{total}}}\left({{{\rm{classifier}}}}\right)}={w}_{{{{\rm{true}}}}}\nabla {L}_{{{{\rm{true}}}}\left({{{\rm{classifier}}}}\right)}+{w}_{{{{\rm{smooth}}}}}\nabla {L}_{{{{\rm{smooth}}}}\left({{{\rm{classifier}}}}\right)}$$

By dynamically adjusting ${w}_{{{{\rm{true}}}}}$ and ${w}_{{{{\rm{smooth}}}}}$, the model can effectively prioritize the loss terms that show significant changes in the optimization process.

### MuSIC model training

We applied the label smoothing strategy to smooth the one-hot label distribution and calculated the total loss as described above. The gradient weight adaptation dynamically modulated relative weights between ${L}_{{{{\rm{true}}}}}$ and ${L}_{{{{\rm{smooth}}}}}$ according to the ratio of the current loss to the previous one, ensuring efficient gradient propagation.

The parameters in MuSIC model were optimized by minimizing the total loss, this loss integrates the classification loss and the smoothed label loss. We used Adam to optimize the model parameters, and Gradual Warmup Scheduler to adjust the learning rates. Throughout model training, we periodically evaluated the prediction performance of the model on the validation set to find the optimized parameters for the model.

### MuSIC hyperparameter settings

The hyperparameters of MuSIC were empirically adjusted based on the cross-species datasets. The hyperparameter settings for MuSIC are defined as follows:

Dimensionality reduction for RNA embeddings: dimensionality reduction uses a learnable linear projection that maps the RNA embeddings from 1280 to 64 dimensions.

Up-sampling RNA structure features: RNA structural features are a learnable linear projection that expands the feature dimension from 2 to 64.

Convolutional layer: the convolutional layer uses a kernel size of (3, 3) with 16 output channels, followed by Batch Normalization (BN) and ReLU activation for non-linearity.

Pooling layer: average pooling is applied with a kernel size of (6, 1), which reduces the feature map along the first axis of the input tensor.

Full connection layer: the fully connected layer at the end of the network has 1 output unit, which corresponds to the predicted binding probability for each sample.

MLP module: RBP–RNA joint representations are further processed by a MLP consisting of 2 hidden layers that progressively reduce the feature dimension from 1024 to 1.

Dropout probability: dropout is applied with probabilities of 0.1, 0.5, and 0.3 at different layers in the network to prevent overfitting.

L2 norm penalty: the L2 norm penalty (weight decay) is set to $1\times {10}^{-6}$, which applies regularization to the model’s weights during optimization.

Batch size: the batch size is set to 64 for training and evaluation.

Learning rate: the learning rate is set to 0.001 for the Adam optimizer, which is used to optimize the network’s parameters.

Positive weight in loss function: the positive weight in the loss function is set to 1, which accounts for class imbalance in the dataset, giving more importance to positive samples during training.

Training epochs: the number of training epochs is set to 200, which determines how many times the model will iterate over the entire training dataset.

Early stop: early stopping is applied with a patience of 20 epochs, meaning training will halt if the model’s performance does not improve on the validation set for 20 consecutive epochs.

Initial weights: the initial weights of the model are set using the Kaiming normal initialization for convolutional layers and Xavier initialization for fully connected layers.

Training was conducted on two NVIDIA A800 GPUs, each with 80GB of memory.

### Ablation experiments of MuSIC model

We performed the ablation experiments to explore to what degree the features and the frameworks could enhance the model performance: (i) input sequence length: we tried different input sequence lengths of 101nt, 200nt, 300nt, 400nt, 500nt, 1000nt and 2000nt. (ii) sequence and structural features : we constructed the models using different types of input data, including only sequences, only predicted structures, combined sequences and predicted structures, combined sequences and randomized structures, combined sequences and experimentally-measured structures, and combined embeddings and predicted structures. (iii) transfer learning frameworks: we compared the proposed label smoothing strategy with domain adaptation approaches^90–93^. (iv) pre-trained models: we constructed the models using embeddings derived from different RNA foundation models^45,46^. (v) classifiers: we benchmarked MuSIC against a range of conventional machine learning and deep learning classifiers (Supplementary Data 3).

### Performance comparison with other computational methods

We compared the prediction performance of MuSIC with other recently published computational methods, including PaRPI (https://github.com/ljquanlab/PaRPI)^32^, HDRNet (https://github.com/zhuhr213/HDRNet)^31^, PrismNet (https://github.com/kuixu/PrismNet)^30^, DeepBind (https://github.com/jisraeli/DeepBind)^28^, and GraphProt (https://github.com/dmaticzka/GraphProt)^26^. Notably, to make a fair comparison, each of these computational methods was trained and validated on the same datasets as used in MuSIC, using their default parameter settings.

We extracted the features from the fully connected layers of MuSIC and the other five computational methods, including PaRPI^32^, HDRNet^31^, PrismNet^30^, DeepBind^28^, and GraphProt^26^. These features were projected into a two-dimensional space using t-SNE and FID to visualize and compare their classification performance.

We used MuSIC and the other five computational methods to train cross-species models and predict RBP-binding peaks for 22 RBPs from mouse, one RBP from zebrafish and 3 RBPs from fly. We then estimated the prediction performance of the computational methods based on the overlap between the predicted peaks with binding probabilities above 0.8 and the experimentally-derived peaks. To further assess the robustness of this evaluation, we systematically tested multiple prediction thresholds (0.9, 0.8, 0.7, and 0.6) and examined their effects on the degree of overlap. In addition, we calculated the correlation between the predicted peaks and experimentally-derived peaks as we did in the meta-transcript analysis mentioned above.

### Thresholding high-confidence RBP across species predicted by MuSIC

We considered the six RBP features for RBP conservation analysis, including the full-length sequence similarity, the full-length structural TM-score, the full-length structural pLDDT, the RBD sequence similarity, the RBD structural TM-score, and the RBD structural pLDDT. We applied a RBP conservation score by assigning each feature a weight proportional to its Pearson correlation with cross-species prediction performance. The conservation score was computed as a normalized linear combination of the six similarity features.

We then analyzed the correlation between the RBP conservation scores and the AUC scores of 17 RBPs derived from the cross-species analysis. We observed a linear correlation and constructed a regression model between the two variables, which can be used for estimating the cross-species prediction accuracy (i.e., AUC score) of the peaks (Supplementary Data 5). We applied a threshold of 0.75 for the AUC scores to retain the high-confidence predicted peaks.

### Identifying and clustering RBP-binding motifs

A total of 2018 models for predicting RBP–RNA interactions in 11 species were trained using 184 high-quality peak datasets from human^33^. RNA sequences from each species were segmented into the fragments of 200nt in length, and 20,000 fragments were randomly sampled for further analysis. In each species, each of the MuSIC models of the 184 RBPs generated predicted peaks on these fragments. We then applied MEME^94^ v5.5.7 to identify the enriched RBP-binding motifs using the fragments with binding probabilities above 0.9.

We performed motif clustering in two steps. First, for each motif, we calculated base frequency by averaging the nucleotide frequencies (A, C, G, U) from its position weight matrix (PWM) as follows:

26 $${{{{\bf{B}}}}}_{i}=\left({A}_{i},{C}_{i},{G}_{i},{U}_{i}\right)$$

where *A*_*i*_*, C*_*i*_*, G*_*i*_*, U*_*i*_ are the average frequencies of adenine (A), cytosine (C), guanine (G), and uracil (U) for the motif *i*, respectively.

Second, we calculated the similarity matrix by integrating the Jensen–Shannon divergence (${{\mbox{JSdiv}}}$) of PWM distributions and the Euclidean distance between base frequency vectors:

27 $${S}_{{ij}}=\alpha {{{\rm{JS}}}}\left(i,j\right)+\left(1-\alpha \right){{{{\rm{d}}}}}_{{{\mbox{euclid}}}}\left(i,j\right)$$

28 $${{\rm{JS}}}\left(i,j\right)=\frac{1}{8}{\sum }_{k=1}^{8}{\mbox{JSdiv}}\left({p}_{i}^{\left(k\right)},{p}_{j}^{\left(k\right)}\right)$$

29 $${{{{\rm{d}}}}}_{{{\mbox{euclid}}}}\left(i,j\right)=\sqrt{{\left({A}_{i}-{A}_{j}\right)}^{2}+{\left({C}_{i}-{C}_{j}\right)}^{2}+{\left({G}_{i}-{G}_{j}\right)}^{2}+{\left({U}_{i}-{U}_{j}\right)}^{2}}$$

where ${p}_{i}^{\left(k\right)}$ is the $k$-th row of the PWM matrix for motif $i$, ${p}_{j}^{\left(k\right)}$ is the $k$-th row of the PWM matrix for motif $j$, ${{{{\rm{d}}}}}_{{{\mbox{euclid}}}}\left(i,j\right)$ is the Euclidean distance between ${{{\bf{B}}}}$ of motif $i$ and $j$, $\alpha$ is a hyperparameter balancing the weight of the JS divergence and Euclidean distance.

Finally, we grouped the motifs using hierarchical clustering to show their overall similarity. We used the itol.toolkit package^95^ v1.1.10 to visualize and map the motif logo to the clustering results.

### Analyzing conservation patterns of RBP-binding peaks predicted by MuSIC

Using human transcripts as the reference, we applied the Mashmap^96^ v3.1.3 to compare pairwise species (i.e., human and non-human species) and select the highest conserved transcripts with segment length > 500nt. For each species, MuSIC was used to predict RBP-binding peaks with 200nt and high attention regions^30^ with 40nt for 184 RBPs in these conserved transcripts.

The conserved transcripts from 11 species were subjected to multi-sequence alignment by using Muscle5^97^ v5.1 with default parameters, and high attention region coordinates were aligned accordingly. RBP-binding peaks overlap counts across species were statistically analyzed within an extended window of 200nt. We then used IGV^98^ v2.16.2 to visualize the RBP-binding peaks in the conserved transcripts from 11 species.

### Evaluating consistency between MuSIC predictions and experimental data for SNV effects

We downloaded the eCLIP peaks of 103 RBPs in HepG2 cell line from ENCODE. The SNVs in the RBP-binding peaks of each RBP were then identified from the eCLIP mapping files (i.e., BAM files) using the BCFtools mpileup^99^ v1.20, where the replicates were merged. Peaks regions were filtered (p.adj ≤ 0.05) and merged across three replicates to retain high-confidence RBP-binding peaks. Using BEDTools^100^ v2.27.1, SNV positions were overlapped with peak regions to classify peaks into SNV-overlapping and non-overlapping groups. A paired t-test was then applied to compare signal differences between the two groups.

We analyzed the RNA-seq alignment files from the smartSHAPE datasets (GEO accession: GSE145805) to estimate the background allele ratio. For both eCLIP and RNA-seq datasets, we calculated their reference allele (REF) and alternative allele (ALT) ratios using BCFtools mpileup^99^ v1.20, and then obtained ${{{{\rm{diff}}}}}_{{{{\rm{ratio}}}}}$, the difference between the REF and ALT ratios from the RNA-seq and eCLIP datasets as follows:

30 $${{{{\rm{diff}}}}}_{{{{\rm{ratio}}}}}=\frac{{{{{\rm{eCLIP}}}}}_{{{{\rm{ALTratio}}}}}}{{{{{\rm{RNAseq}}}}}_{{{{\rm{ALTratio}}}}}}-\frac{{{{{\rm{eCLIP}}}}}_{{{{\rm{REFratio}}}}}}{{{{{\rm{RNAseq}}}}}_{{{{\rm{REFratio}}}}}}$$

In parallel, we also calculated ${{{{\rm{diff}}}}}_{{{{\rm{score}}}}}$, the predicted difference between the REF and ALT ratios as follows:

31 $${{{{\rm{diff}}}}}_{{{{\rm{score}}}}}={{{{\rm{Pred}}}}}_{{{{\rm{ALT}}}}}-{{{{\rm{Pred}}}}}_{{{{\rm{REF}}}}}$$

where ${{{{\rm{Pred}}}}}_{{{{\rm{ALT}}}}}$ and ${{{{\rm{Pred}}}}}_{{{{\rm{REF}}}}}$ are predicted binding possibilities for the sequences containing the ALT and REF, respectively.

We pre-processed the SNV effects by retaining |${{{{\rm{diff}}}}}_{{{{\rm{ratio}}}}}$ | ≥ 0.2 and |${{{{\rm{diff}}}}}_{{{{\rm{score}}}}}$ | ≥ 0.2, and then extracting the top 80% ranked by ${{{{\rm{diff}}}}}_{{{{\rm{score}}}}}$. We used the experimental labels (${{{{\rm{label}}}}}_{\exp }$) and the predicted labels (${{{{\rm{label}}}}}_{{{{\rm{pre}}}}}$) to evaluate the consistency between experimental data and MuSIC predictions for SNV effects. The ${{{{\rm{label}}}}}_{\exp }$ and ${{{{\rm{label}}}}}_{{{{\rm{pre}}}}}$ are defined as follows:

$${{{{\rm{label}}}}}_{\exp }=1\,{{{\rm{when}}}}\,{{{{\rm{diff}}}}}_{{{{\rm{ratio}}}}}\ge 0;{{{{\rm{label}}}}}_{\exp }=0{{{\rm{when}}}}\,{{{{\rm{diff}}}}}_{{{{\rm{ratio}}}}} < 0$$

$${{{{\rm{label}}}}}_{{{{\rm{pre}}}}}=1\,{{{\rm{when}}}}\,{{{{\rm{diff}}}}}_{{{{\rm{score}}}}}\ge 0;{{{{\rm{label}}}}}_{{{{\rm{pre}}}}}=0{{{\rm{when}}}}\,{{{{\rm{diff}}}}}_{{{{\rm{score}}}}} < 0$$

The consistency between ${{{{\rm{label}}}}}_{\exp }$ and ${{{{\rm{label}}}}}_{{{{\rm{pre}}}}}$ was evaluated using Fisher’s exact test, with multiple testing corrected by the Benjamini–Hochberg method, and results with FDR ≤ 0.05 were considered statistical significance.

### Quantifying the effects of pathogenic and benign SNVs on RBP-binding using MuSIC

We downloaded SNVs curated in the ClinVar^7^. Variants were categorized into pathogenic (Pathogenic; Likely_pathogenic; Pathogenic/Likely_pathogenic) and benign (Benign; Likely_benign; Benign/Likely_benign) SNVs according to their annotated clinical significance. For each SNV, human RNA sequences were extracted using GAP, and paired RNA sequences containing the reference (REF) and alternative (ALT) alleles were generated.

We then predicted RBP–RNA binding probabilities on both REF- and ALT-containing sequences for 184 RBPs using MuSIC. The effect of the RBP-binding affinity was quantified as the absolute difference between the predicted binding probabilities of the ALT and REF alleles:

32 $$\Delta \,{{{\rm{score}}}}=\left|{{{{\rm{P}}}}}_{{{{\rm{ALT}}}}}-{{{{\rm{P}}}}}_{{{{\rm{REF}}}}}\right|$$

where ${{{{\rm{P}}}}}_{{{{\rm{ALT}}}}}$ and ${{{{\rm{P}}}}}_{{{{\rm{REF}}}}}$ denote the predicted binding probabilities for the ALT and REF alleles, respectively.

### Quantifying the effects of homologous SNVs between human and mouse on RBP-binding using MuSIC

We downloaded the human and mouse RNA sequences from Ensembl^101^ (Release 113). We extracted the human RNA sequences overlapping with the nonsense SNV positions from dbSNP^6^ using the GAP, and then generated the two sequences containing the REF and ALT for each SNV. We then identified the homologous regions of the human SNV-containing sequences in the mouse genome using BLAST^102^ v2.16.0. In total, 32,207 nonsense SNVs in human, and 85,599 homologous nonsense SNVs in mouse were included for further analyses.

We then used MuSIC to predict the binding possibilities on the RNA sequences containing the REF and ALT in human and mouse for each of the 184 RBPs. For each sequence, the effect of the SNV on RBP binding was defined as follows:

23 $$\Delta {{{\rm{SNV}}}}={{{{\rm{P}}}}}_{{{{\rm{ALT}}}}}-{{{{\rm{P}}}}}_{{{{\rm{REF}}}}}$$

where P_ALT_ is the binding probability for the ALT, P_REF_ is the binding probability for the REF.

### Plasmid construction

The myc-tagged zebrafish Tra2a and Hnrnpk coding sequences were codon-optimized for expression in HEK293T cells, synthesized by Azenta (Beijing, China), and PCR-amplified using 2× Phanta Max Master Mix (Vazyme). The myc-tagged mouse Tra2a, Hnrnpk and Pum2 coding sequences were PCR-amplified from V6.5 mouse embryonic stem cell–derived cDNA. The myc-tagged human PUM2 coding sequences were PCR-amplified from HEK293T cell–derived cDNA. All RBP coding sequences were cloned into a piggyBac backbone vector under the control of a CAGGS promoter using the Hieff Clone® Plus One Step Cloning Kit (Yeasen).

For expression of human PUM2 and mouse Pum2 SNV targets, wild-type (REF) and SNP variant (ALT) oligonucleotides were synthesized and annealed by heating to 95 °C for 5 min, followed by gradual cooling to 25 °C. The REF and ALT sequences were cloned into the 3′ untranslated region (3′UTR) of GFP, which is driven by a CAGGS promoter, using T4 DNA ligase (Thermo Fisher Scientific).

The EPN24–hPUM2 and EPN24–mPum2 constructs were generated by assembling EPN24 (synthesized by Azenta) with hPUM2 or mPum2 coding sequences into a piggyBac backbone vector under the control of a CAGGS promoter, using 2× MultiF Seamless Assembly Mix (ABclonal).

All constructs were validated by Sanger sequencing. The RBP sequences and the primer sequences used for RBP cloning are listed in Supplementary Data 5 and Supplementary Data 7.

### Cell transfection

For RNA pull-down assays, 2 × 10⁶ HEK293T cells per plate were seeded onto poly-D-lysine–coated 10-cm dishes. After 16–20 h, cells were transfected with 5–10 µg myc-tagged RBP plasmids using JetPRIME reagent (Polyplus). Cells were harvested 48 h post-transfection for subsequent RNA pull-down assays.

For POND-qPCR assay, a total of 150,000 HEK293T cells per well were seeded into poly-D-lysine–coated 24-well plates. After 16–20 h, cells were co-transfected with 200 ng of EPN24-PUM2 plasmid together with 75 ng of either GFP-REF or GFP-ALT target plasmid using JetPRIME (Polyplus). The culture medium was replaced 4–6 h post-transfection, and cell culture supernatants were collected 24 h after medium replacement for subsequent analysis.

### RNA pull-down assay

The in vitro RNA pull-down assay was performed as followed^103^. Briefly, 200 pmol of biotinylated RNA oligonucleotides were refolded by heating at 90 °C for 1 min, followed by incubation at 30 °C for 5 min. ~1 × 10⁷ HEK293T cells expressing Myc-tagged RBP were lysed in 500 µl of lysis buffer (150 mM NaCl, 1 mM EDTA, 1% Triton X-100, 0.5 mM DTT, 50 mM Tris-HCl, pH 7.5, 0.5% sodium deoxycholate) supplemented with 5 µl PMSF (100 mM; Beyotime), 10 µl phosphatase inhibitor cocktail (50×; Beyotime), 10 µl protease inhibitor cocktail (50×; Beyotime), 2.5 µl SUPERase•In RNase inhibitor (20 U µl⁻¹; Invitrogen), and 5 µl RNase inhibitor (40 U µl⁻¹; Beyotime). Refolded RNA was incubated with 500 µl of cell lysate at 4 °C for 3–4 h. Subsequently, 100 µl of pre-washed streptavidin beads (VAHTS CA-28; Vazyme) were added and incubated at 4 °C for 1–2 h. Beads were washed once with high-salt buffer (50 mM Tris-HCl, pH 7.5, 1 M NaCl, 1% Triton X-100) at room temperature for 5 min, followed by two washes with low-salt buffer (50 mM Tris-HCl, pH 7.5, 150 mM NaCl, 1% Triton X−100) under the same conditions. Bound proteins were eluted in 50 µl of 1× SDS–PAGE loading buffer (Beyotime) by heating at 98 °C for 20 min.

Protein electrophoresis was performed using 8–16% BeyoGel™ Elite precast polyacrylamide gels (Beyotime), followed by transfer of proteins to polyvinylidene fluoride (PVDF) membranes. The membranes were incubated with HRP-conjugated mouse monoclonal anti-Myc antibody (Beyotime, AF2867; 1:1000) at 4 °C overnight, or with anti-β-actin antibody (CST, 4970; 1:10,000) for 1 h at room temperature, followed by incubation with HRP-conjugated secondary antibodies (Beyotime, A0208; 1:1000) for 1 h at room temperature. After three washes with 1× TBST, signals were detected using enhanced chemiluminescence (ECL) reagent (Merck Millipore, WBKLS0050) and visualized using an eBLOT14 Touch Imager (e-BLOT).

### POND-qPCR

Cell culture supernatants were clarified by centrifugation at 3,000×g for 5 min, followed by filtration through a 0.45 µm cellulose acetate filter (Corning). An aliquot of 200 µl supernatant was mixed with 800 µl TRIzol reagent (Magen) and 1 µl of diluted External RNA Controls Consortium (ERCC) synthetic spike-in RNAs (1:1,000; Thermo Fisher Scientific). RNA was extracted according to the manufacturer’s instructions and resuspended in 20 µl nuclease-free water.

For reverse transcription, 3 µl of supernatant RNA or 250 ng of total cellular RNA was reverse transcribed using HiScript® III RT SuperMix (Vazyme) at 37 °C for 15 min. The resulting cDNA was diluted fivefold with nuclease-free water, and 1 µl was used for quantitative real-time PCR using AceQ qPCR SYBR Green Master Mix (Vazyme) in 96-well plates on a StepOnePlus Real-Time PCR System (Applied Biosystems).

The qPCR primer sequences are listed in Supplementary Data 7.

### Large-scale screening of RBPs affected by SNVs in UPS

For each RBP, SNVs with strong effects were identified: $\Delta {{{\rm{SNV}}}}$ < −0.2 and ${{{{\rm{P}}}}}_{{{{\rm{REF}}}}}$ > 0.6. We annotated the transcripts affected by SNVs using the GAP.

We used the STRING^104^ database (https://string-db.org/) to annotate the enriched biological functions of SNV-affected transcripts. Among the top five enriched biological functions, we selected those related to ubiquitination or deubiquitination. Then, we categorized the RBPs into four groups according to the functional enrichment with ubiquitination or deubiquitination for their target transcripts affected by SNVs, i.e., the highly ubiquitin-related (both ubiquitination- and deubiquitination-related), the weakly ubiquitin-related (either ubiquitination- or deubiquitination-related), the not ubiquitin-related (not ubiquitination- or deubiquitination-related), and the not function-enriched.

We then performed the KEGG enrichment analysis on the SNV-affected transcripts annotated with highly ubiquitin-related. The enriched disease pathways revealed potential regulatory associations among SNVs, RBPs, and disease-relevant UPS genes.

### Quantification and statistical analysis

All statistical analyses were performed with R v4.4.0. Where applicable, the sample size (n) was specified either in the plot or in the corresponding figure legends. Analysis details could be found in the method section, such as statistical tests, definitions, etc. Box plots were used to visualize the distribution of the data, with the box representing the interquartile range (IQR). The median was indicated by a horizontal line within the box, while the upper and lower hinges represented the 75^th^ and 25^th^ percentiles, respectively. The whiskers extended to the most extreme data points within 1.5 times the IQR from the box. Any data points beyond this range were considered outliers and plotted individually. Statistical significance was determined using appropriate tests, with p reported in the figure panels or legends.

### Reporting summary

Further information on research design is available in the Nature Portfolio Reporting Summary linked to this article.

## Supplementary information

Supplementary Information

Description of Additional Supplementary Files

Supplementary Data 1

Supplementary Data 2

Supplementary Data 3

Supplementary Data 4

Supplementary Data 5

Supplementary Data 6

Supplementary Data 7

Supplementary Data 8

Reporting Summary

Transparent Peer Review file

## Source data

Source Data

## Data Availability

The 216 RBP sequences for the 11 species used in this study were downloaded from UniProt^83^ database (https://www.uniprot.org/). The corresponding entry IDs are listed in Supplementary Data 1 file. The 18S rRNA reference sequences for the 11 species were downloaded from RNAcentral^81^ database under the following accession IDs: *Homo sapiens* (https://rnacentral.org/search?q=URS0000726FAB_9606), *Pongo abelii* (https://rnacentral.org/search?q=URS000302FF38_9601), *Macaca fascicularis* (https://rnacentral.org/search?q=URS00005800AF_1035826), *Mus musculus* (https://rnacentral.org/search?q=URS00005B0A54_10090), *Rattus norvegicus* (https://rnacentral.org/search?q=URS0002A14804_10116), *Gallus gallus* (https://rnacentral.org/search?q=URS000263BE37_9031), *Xenopus laevis* (https://rnacentral.org/search?q=URS0002349D5A_8355), *Danio rerio* (https://rnacentral.org/search?q=URS0000668FC4_7955), *Drosophila melanogaster* (https://rnacentral.org/search?q=URS0000A2DABB_7227), *Arabidopsis thaliana* (https://rnacentral.org/search?q=URS00021C577A_3702), and *Saccharomyces cerevisiae* (https://rnacentral.org/search?q=URS0000B21DE0_559292). The 3D structures of RBP-RNA interactions were obtained from the Protein Data Bank (PDB)^88^ database with the following entry IDs: Human: 4QIL and Mouse: 4QI2. The CLIP datasets used for model training, validation, and RNA pattern conservation analysis is available in POSTAR3^33^ database (https://cloud.tsinghua.edu.cn/d/8133e49661e24ef7a915/). The fly RBP-binding datasets used for model training and validation is available in ENCODE database under accession code ENCSR432JLI^55^ (https://www.encodeproject.org/experiments/ENCSR432JLI/). The eCLIP data for 103 RBPs used in the SNV impact analysis was downloaded from the ENCODE database under the accession number ENCSR456FVU^61^ (https://www.encodeproject.org/publication-data/ENCSR456FVU/). The smartSHAP sequencing dataset, used as background data, is available under the GEO accession number GSE145805^30^ (https://www.ncbi.nlm.nih.gov/geo/query/acc.cgi?acc=GSE145805). The foundation models and pre-trained weights were obtained from the following publicly available repositories: RNA-FM^45^ (https://github.com/ml4bio/RNA-FM), RiNALMo^46^ (https://github.com/lbcb-sci/RiNALMo), ProtT5^48^ (https://github.com/agemagician/ProtTrans). All oligonucleotides used for experimental validation in this study, including primers and RNA sequences, as well as the RBP sequences and plasmids, are provided in Supplementary Data 5 and 7 and the Source Data file. Source data are provided with this paper.

## Code availability

The MuSIC^105^ scripts and test data are available at (https://github.com/GALE1228/MuSIC_pretrain). The large datasets are deposited on Zenodo at (10.5281/zenodo.19353389).
