# Supplementary material for: Augmented prediction of multi-species protein–RNA interactions using evolutionary conservation of RNA-binding proteins
Source: Nat Commun. 2026 Apr 27;17:5764. doi: 10.1038/s41467-026-72351-6 (PMC13324433; doi:10.1038/s41467-026-72351-6)
Supplement: Supplementary file 1 — Supplementary Information [file 41467_2026_72351_MOESM1_ESM.pdf]

Figure S1

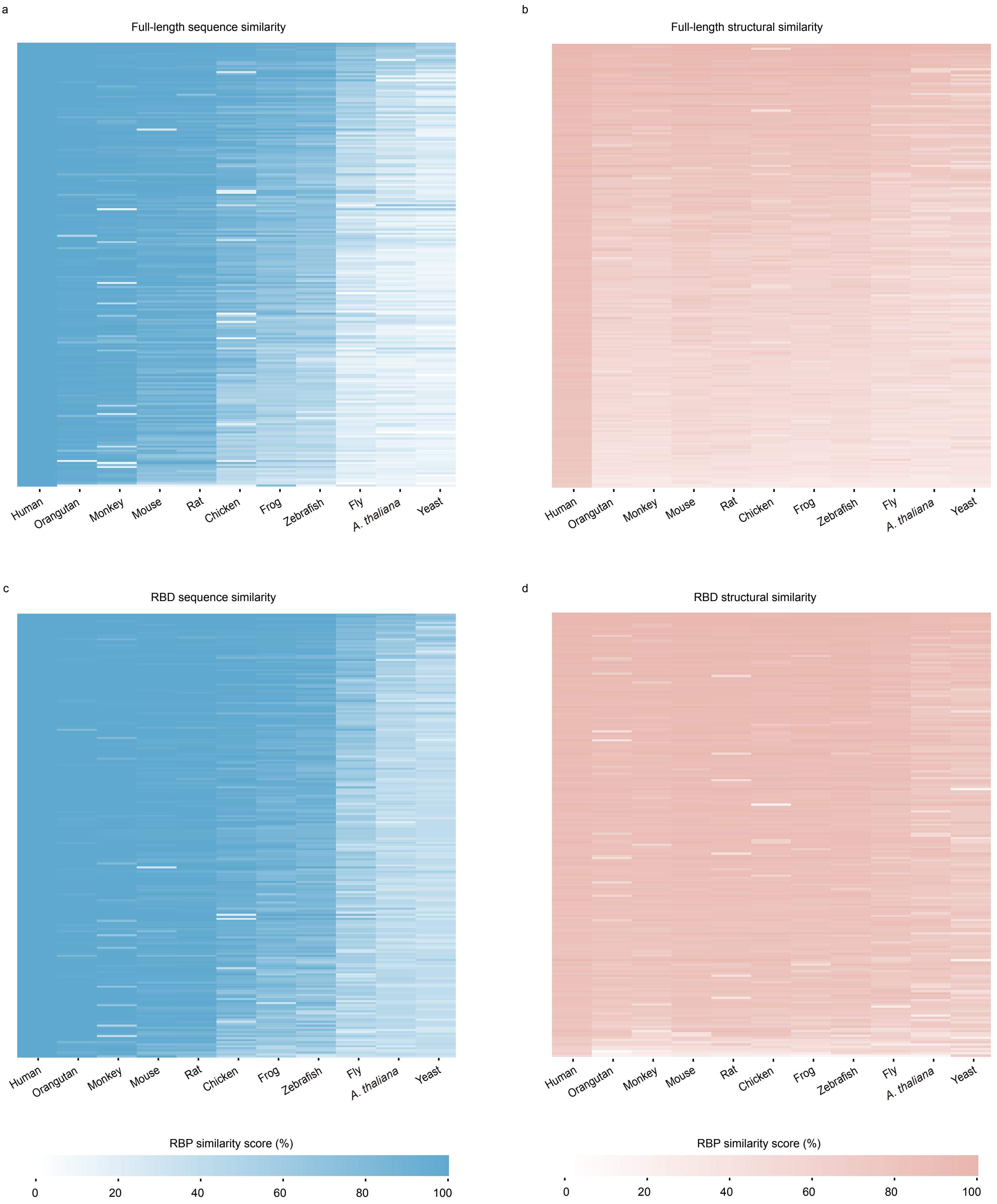

a
Full-length sequence similarity
Human
Orangutan
Monkey
Mouse
Rat
Chicken
Frog
Zebrafish
Fly
A. thaliana
Yeast
b
Full-length structural similarity
Human
Orangutan
Monkey
Mouse
Rat
Chicken
Frog
Zebrafish
Fly
A. thaliana
Yeast
c
RBD sequence similarity
Human
Orangutan
Monkey
Mouse
Rat
Chicken
Frog
Zebrafish
Fly
A. thaliana
Yeast
d
RBD structural similarity
Human
Orangutan
Monkey
Mouse
Rat
Chicken
Frog
Zebrafish
Fly
A. thaliana
Yeast
RBP similarity score (%)
0
20
40
60
80
100
RBP similarity score (%)
0
20
40
60
80
100

Supplementary Figure 1: Evolutionary conservation of RBPs across 11 species

(a-b) Heatmaps showing the full-length sequence similarity (a) and structural similarity (b) of RBPs across species. (c-d) Heatmaps showing the RBD sequence similarity (c) and structural similarity (d) of RBPs across species. Source data are provided as a Source Data file.

Figure S2

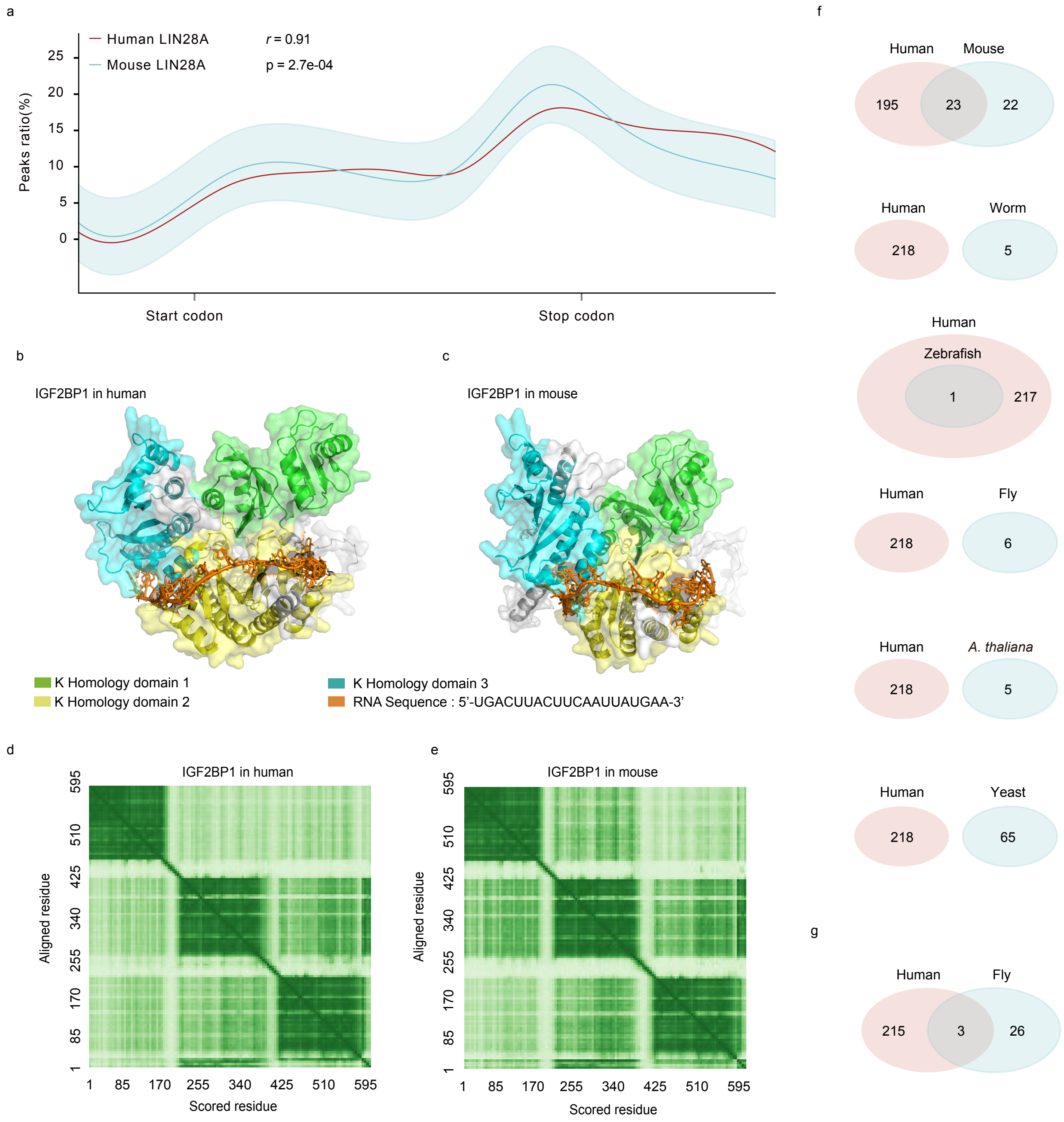
a
Human LIN28A
Mouse LIN28A
r = 0.91
p = 2.7e-04
25
20
15
10
5
0
Peaks ratio(%)
Start codon
Stop codon
b
IGF2BP1 in human
c
IGF2BP1 in mouse
K Homology domain 1
K Homology domain 2
K Homology domain 3
RNA Sequence : 5'-UGACUUACUUCAAUUAUGAA-3'
d
IGF2BP1 in human
e
IGF2BP1 in mouse
Aligned residue
595
510
425
340
255
170
85
1
1
85
170
255
340
425
510
595
Scored residue
f
Human
Mouse
195
23
22
Human
Worm
218
5
Human
Zebrafish
1
217
Human
Fly
218
6
Human
A. thaliana
218
5
Human
Yeast
218
65
g
Human
Fly
215
3
26

Supplementary Figure 2: Evolutionary conservation of RBP-targeted RNAs

(a) LIN28A binding distribution along meta-transcript from human and mouse. Data are presented as mean values +/- SD (Spearman's rank correlation test). (b-c) Three-dimensional structures of IGF2BP1 from human (b) and mouse (c), showing the residues of bound RNA and three KH domains. (d-e) Heatmaps showing residue interaction score for IGF2BP1 from human (d) and mouse (e), with darker green represents stronger interaction scores. (f) Venn diagrams showing the overlap of RBPs between different species (human, mouse, zebrafish, fly, worm, *A. thaliana*, and yeast) collected from POSTAR3 database. (g) Venn diagram showing the overlap of RBPs between human and fly collected from modENCODE project.

Figure S3

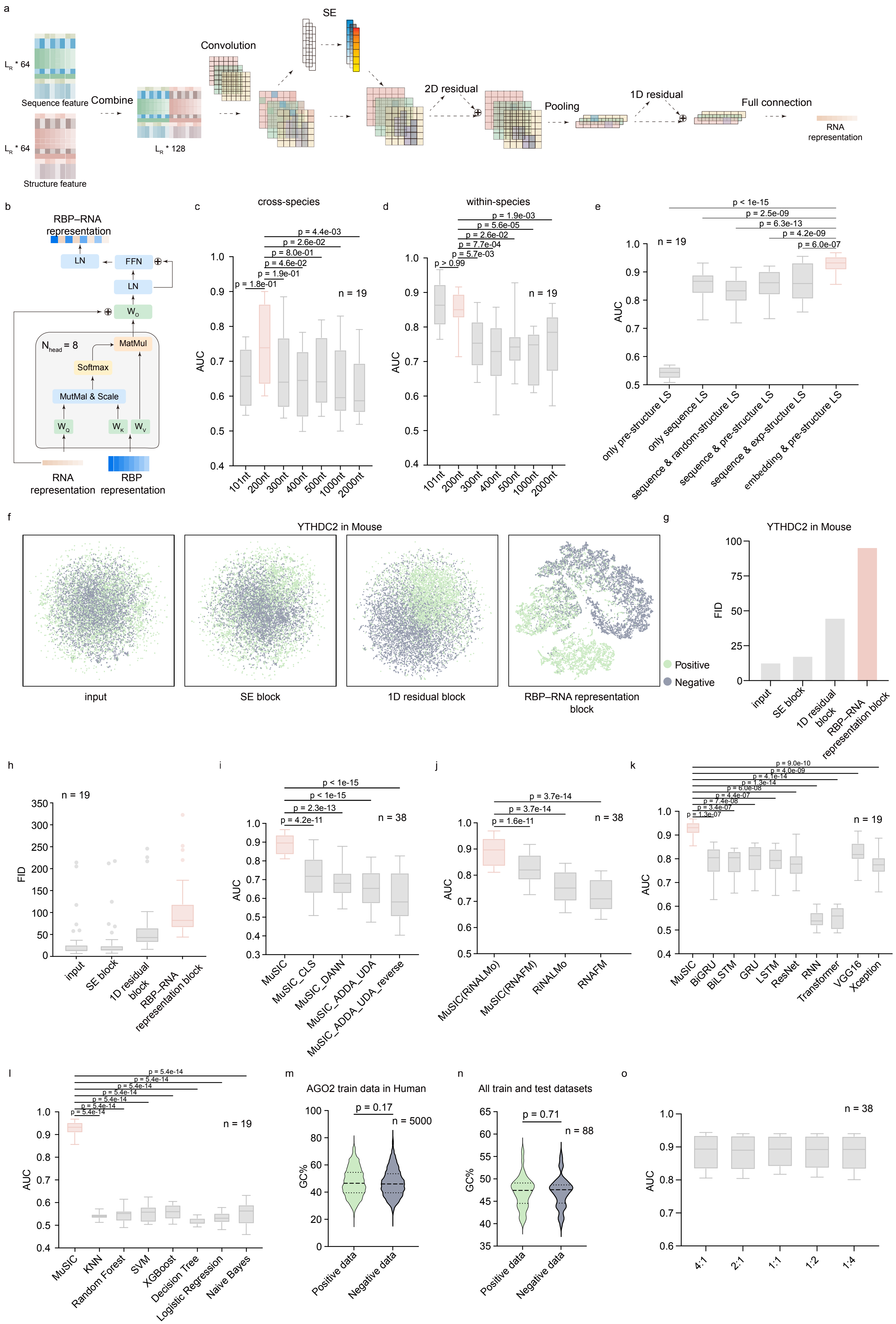
a
Sequence feature
Structure feature
Combine
Convolution
SE
2D residual
Pooling
1D residual
Full connection
RNA representation
b
RBP–RNA representation
LN
FFN
W_O
MatMul
Softmax
MutMal & Scale
W_Q
W_K
W_V
N_{head} = 8
RNA representation
RBP representation
c
cross-species
d
within-species
e
only pre-structure LS
only sequence LS
sequence & random-structure LS
sequence & pre-structure LS
sequence & exp-structure LS
embedding & pre-structure LS
f
YTHDC2 in Mouse
input
SE block
1D residual block
RBP–RNA representation block
Positive
Negative
g
FID
h
i
MuSIC
MuSIC_CLS
MuSIC_DANN
MuSIC_ADDA_UDA
MuSIC_ADDA_UDA_reverse
j
MuSIC(RiNALMo)
MuSIC(RNAFM)
RiNALMo
RNAFM
k
BiGRU
BiLSTM
GRU
LSTM
ResNet
RNN
Transformer
VGG16
Xception
l
KNN
Random Forest
SVM
XGBoost
Decision Tree
Logistic Regression
Naive Bayes
m
AGO2 train data in Human
p = 0.17
n = 5000
Positive data
Negative data
n
All train and test datasets
p = 0.71
n = 88
o
n = 38
4:1
2:1
1:1
1:2
1:4

Supplementary Figure 3: Optimization of input feature and model architecture

(a) Overview of the learning RNA module. The combined RNA features are processed sequentially through the convolutional layers, SE modules, residual blocks, average pooling, and fully connected layer to generate the RNA representations. (b) Overview of the learning interaction module. The RNA and RBP representations are integrated through multi-head cross-attention and a position-wise feed-forward network to generate the joint representations. (c-d) Box plot showing the performance of MuSIC using different input sequence lengths for 19 cross-species datasets (c, n = 19 datasets) and 19 within-species datasets (d, n = 19 datasets). (e) Box plot showing the performance of MuSIC using different input features with 200nt for 19 cross-species datasets (n = 19 datasets). (f) t-SNE clustering showing the output feature maps by different blocks (input, SE block, 1D residual block, RBP–RNA representation block) for separating the positive and negative peaks. (g) Bar plot showing the FID between the positive and negative feature distributions learned by different blocks for YTHDC2. (h) Bar plot showing the FID between the positive and negative feature distributions learned by different blocks for 19 cross-species datasets (n = 19 datasets). (i) Box plot showing the prediction accuracy of the MuSIC with label smooth and four domain adaptation methods for 38 cross-species datasets (n = 38 datasets). (j) Box plot showing the prediction accuracy of the MuSIC with RiNALMo and other RNA foundation models for 38 cross-species datasets (n = 38 datasets). (k) Box plot showing the prediction accuracy of MuSIC and other deep learning models for 19 cross-species datasets (n = 19 datasets). (l) Box plot showing the prediction accuracy of MuSIC and other machine learning models for 19 cross-species datasets (n = 19 datasets). (m) Violin plot showing the GC-content distributions of positive and negative samples (n = 5000 samples) in the human AGO2 training dataset. (n) Violin plot showing the mean GC content of positive and negative samples for 88 training and test datasets (n = 88 datasets). (o) Box plot showing the performance of MuSIC under different training-validation split ratios for 38 cross-species datasets (n = 38 datasets). For each box plot, center line indicates median; box limits indicate the 25th and 75th percentiles; whiskers indicate 1.5× the interquartile range; points beyond the whiskers indicate outliers. For each violin plot, center lines indicate medians and dashed lines indicate quartiles. Statistical tests were as follows: one-way repeated-measures ANOVA with Holm-Sidak multiple-comparisons test in(c,d); one-way repeated-measures ANOVA with Dunnett's multiple-comparisons test in(e,h,i,j,k,l); two-sided Mann-Whitney U test in(m); and two-sided Wilcoxon signed-rank test in(n). Source data are provided as a Source Data file.

Figure S4

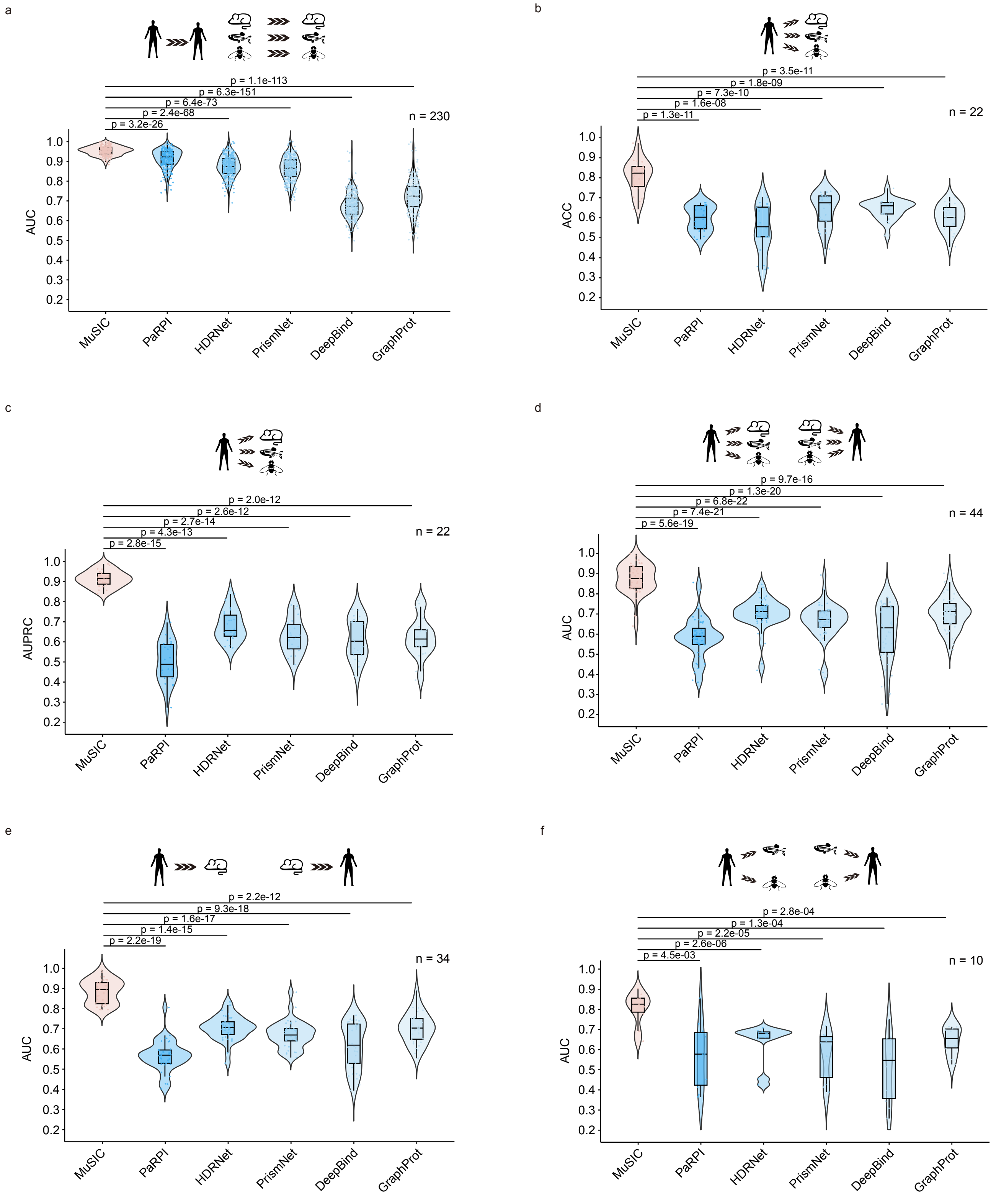

a
p = 1.1e-113
p = 6.3e-151
p = 6.4e-73
p = 2.4e-68
p = 3.2e-26
n = 230
AUC
1.0
0.9
0.8
0.7
0.6
0.5
0.4
0.3
0.2
MuSIC
PaRPI
HDRNet
PrismNet
DeepBind
GraphProt
b
p = 3.5e-11
p = 1.8e-09
p = 7.3e-10
p = 1.6e-08
p = 1.3e-11
n = 22
ACC
c
p = 2.0e-12
p = 2.6e-12
p = 2.7e-14
p = 4.3e-13
p = 2.8e-15
n = 22
AUPRC
d
p = 9.7e-16
p = 1.3e-20
p = 6.8e-22
p = 7.4e-21
p = 5.6e-19
n = 44
AUC
e
p = 2.2e-12
p = 9.3e-18
p = 1.6e-17
p = 1.4e-15
p = 2.2e-19
n = 34
AUC
f
p = 2.8e-04
p = 1.3e-04
p = 2.2e-05
p = 2.6e-06
p = 4.5e-03
n = 10
AUC

Supplementary Figure 4: The performance comparison between MuSIC and other computational methods

(a) Violin plot showing the prediction accuracy of MuSIC and other computational methods for 230 within-species datasets (n = 230 datasets). (b-c) Violin plot showing the prediction accuracy (ACC and AUPRC) of MuSIC and other computational methods for 22 cross-species datasets (n = 22 datasets). (d) Violin plot showing the prediction accuracy of MuSIC and other computational methods for 44 cross-species datasets (n = 44 datasets). (e) Violin plot showing the prediction accuracy of MuSIC and other computational methods for 34 cross-species datasets between human and mouse (n = 34 datasets). (f) Violin plot showing the prediction accuracy of MuSIC and other computational methods for 10 cross-species datasets between human and distantly related species (zebrafish and fly) (n = 10 datasets). For each violin plot, center line indicates median; box limits indicate the 25th and 75th percentiles; whiskers indicate 1.5× the interquartile range. And the statistical test is two-sided paired Student's t-test. Source data are provided as a Source Data file.

Figure S5

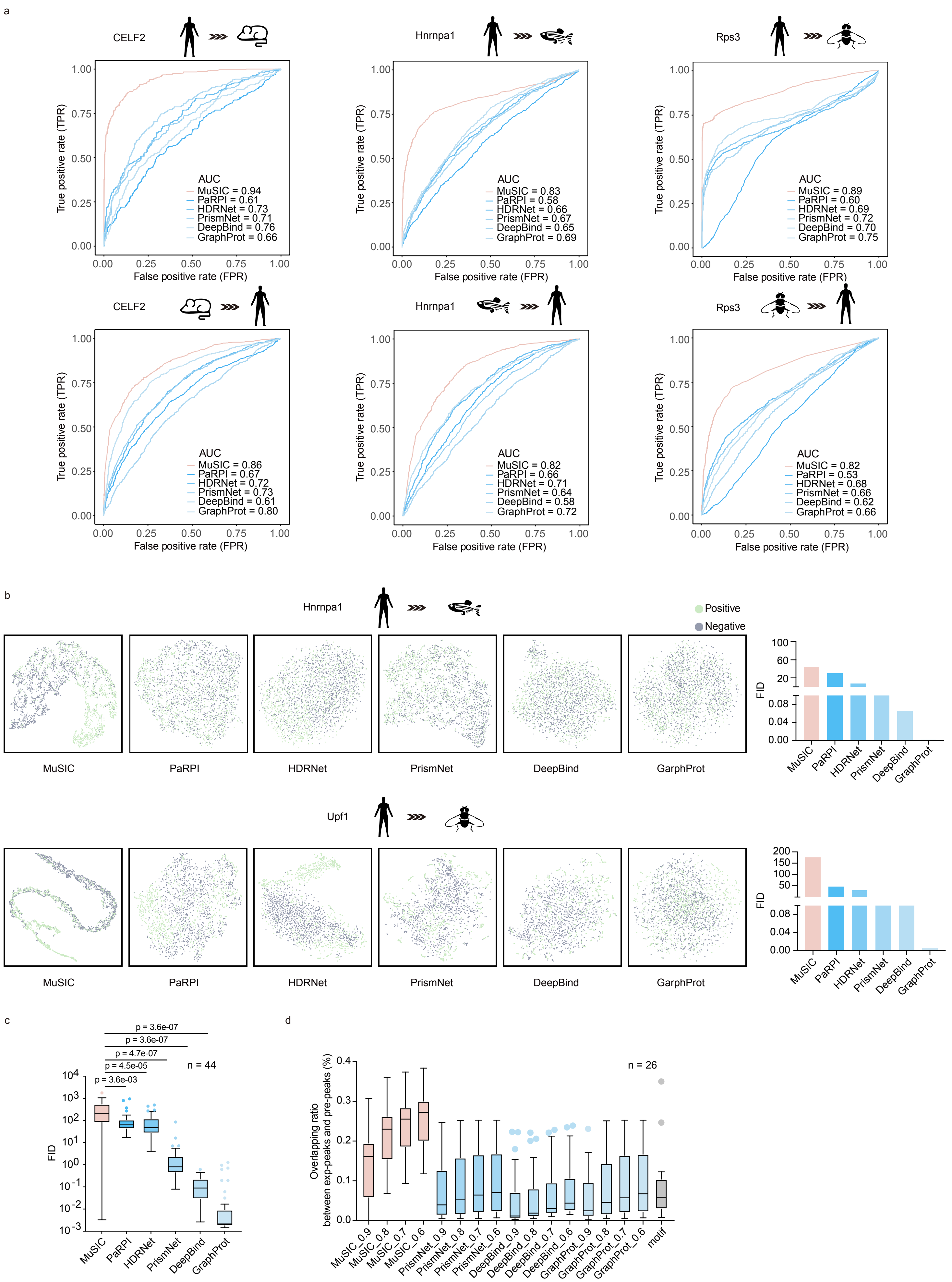

a
CELF2
Hnrnpa1
Rps3
True positive rate (TPR)
False positive rate (FPR)
AUC
MuSIC = 0.94
PaRPI = 0.61
HDRNet = 0.73
PrismNet = 0.71
DeepBind = 0.76
GraphProt = 0.66
MuSIC = 0.83
PaRPI = 0.58
HDRNet = 0.66
PrismNet = 0.67
DeepBind = 0.65
GraphProt = 0.69
MuSIC = 0.89
PaRPI = 0.60
HDRNet = 0.69
PrismNet = 0.72
DeepBind = 0.70
GraphProt = 0.75
MuSIC = 0.86
PaRPI = 0.67
HDRNet = 0.72
PrismNet = 0.73
DeepBind = 0.61
GraphProt = 0.80
MuSIC = 0.82
PaRPI = 0.66
HDRNet = 0.71
PrismNet = 0.64
DeepBind = 0.58
GraphProt = 0.72
MuSIC = 0.82
PaRPI = 0.53
HDRNet = 0.68
PrismNet = 0.66
DeepBind = 0.62
GraphProt = 0.66
b
Hnrnpa1
Upf1
Positive
Negative
MuSIC
PaRPI
HDRNet
PrismNet
DeepBind
GarphProt
FID
c
p = 3.6e-07
p = 3.6e-07
p = 4.7e-07
p = 4.5e-05
p = 3.6e-03
n = 44
d
n = 26
Overlapping ratio between exp-peaks and pre-peaks (%)
MuSIC_0.9
MuSIC_0.8
MuSIC_0.7
MuSIC_0.6
PrismNet_0.9
PrismNet_0.8
PrismNet_0.7
PrismNet_0.6
DeepBind_0.9
DeepBind_0.8
DeepBind_0.7
DeepBind_0.6
GraphProt_0.9
GraphProt_0.8
GraphProt_0.7
GraphProt_0.6
motif

Supplementary Figure 5: Comparison of feature learning discriminative power between MuSIC and other computational methods

(a) ROC curves showing the prediction accuracy of MuSIC and other computational methods. Left, the mouse RBP; middle, the zebrafish RBP; right, the fly RBP. (b) Left: t-SNE clustering showing the output feature maps by MuSIC and other computational methods for separating the positive and negative peaks. Right: Bar plot showing the FID which assesses the distance between the positive and negative feature distributions learned by MuSIC and other computational methods. (c) Box plot showing the FID which assesses the distance between the positive and negative feature distributions learned by MuSIC and other computational methods for 44 cross-species datasets ($n = 44$ datasets; center line, median; box limits, 25th and 75th percentiles; whiskers, 1.5 × interquartile range; points beyond the whiskers, outliers; one-way repeated-measures ANOVA with Holm–Sidak multiple-comparisons test). (d) Box plot showing the overlapping ratio for 26 RBPs between the experimentally-derived and predicted peaks by MuSIC (red), other computational methods (blue) and motif-baseline (grey) across multiple prediction thresholds ($n = 26$ RBPs; center line, median; box limits, 25th and 75th percentiles; whiskers, 1.5 × interquartile range; points beyond the whiskers, outliers). Source data are provided as a Source Data file.

Figure S6

a

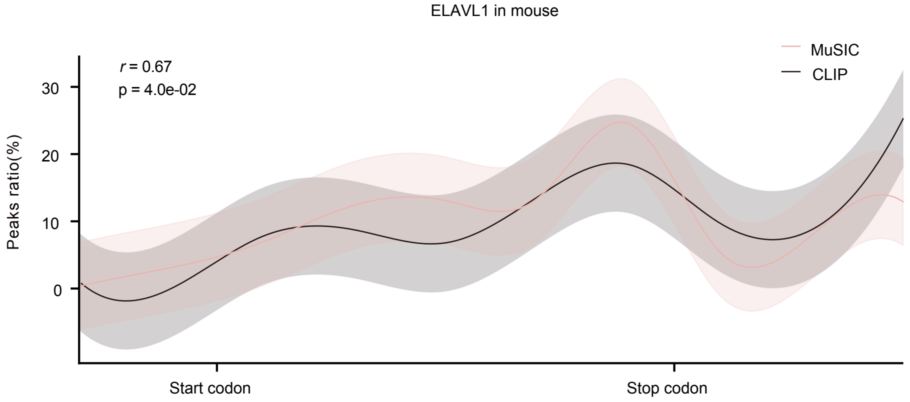

b

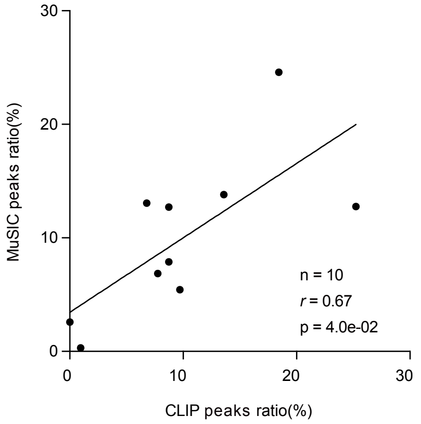

c

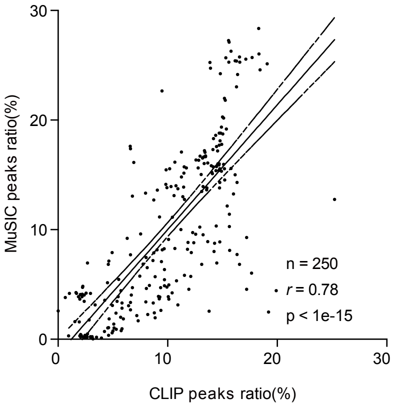

Supplementary Figure 6: Consistency of RBP-binding distributions between MuSIC predictions and experimental data

(a) ELAVL1 binding distributions along meta-transcript in MuSIC predictions and experimentally-derived data. Data are presented as mean values +/- SD (simple linear regression). (b) Scatter plot showing the correlation of RBP-binding distributions from MuSIC predictions and experimental data for ELAVL1 (line, the fitted linear regression line). (c) Scatter plot showing the correlation of RBP-binding distributions from MuSIC predictions and experimental data (line, the fitted linear regression line; error bands, 95% confidence interval). Source data are provided as a Source Data file.

Figure S7

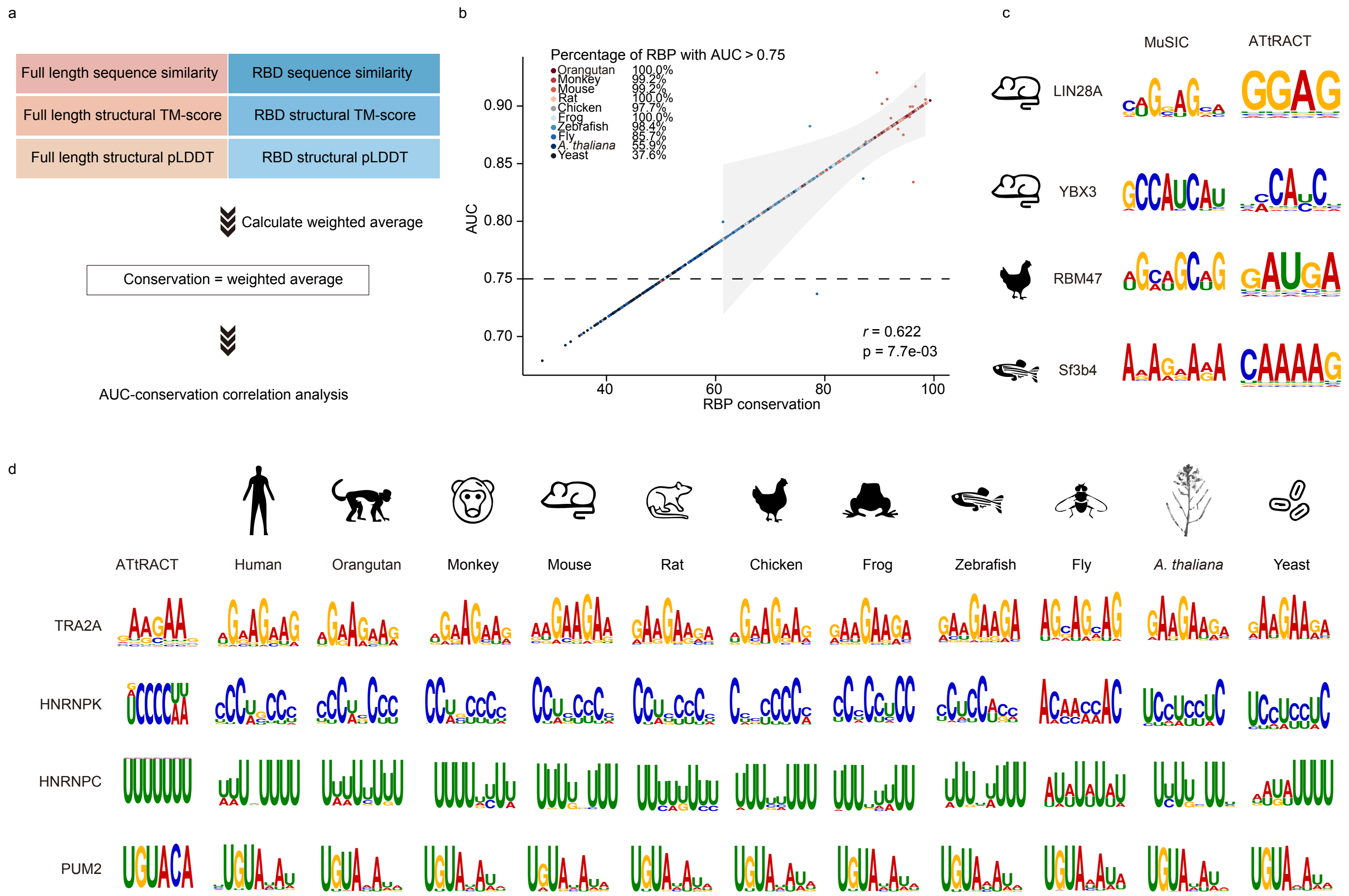
a
Full length sequence similarity
RBD sequence similarity
Full length structural TM-score
RBD structural TM-score
Full length structural pLDDT
RBD structural pLDDT
Calculate weighted average
Conservation = weighted average
AUC-conservation correlation analysis
b
Percentage of RBP with AUC > 0.75
Orangutan 100.0%
Monkey 99.2%
Mouse 99.2%
Rat 100.0%
Chicken 97.7%
Frog 100.0%
Zebrafish 98.4%
Fly 85.7%
A. thaliana 55.9%
Yeast 37.6%
r = 0.622
p = 7.7e-03
AUC
RBP conservation
c
MuSIC
ATtRACT
LIN28A
YBX3
RBM47
Sf3b4
d
ATtRACT
Human
Orangutan
Monkey
Mouse
Rat
Chicken
Frog
Zebrafish
Fly
A. thaliana
Yeast
TRA2A
HNRNPK
HNRNPC
PUM2

Supplementary Figure 7: Evolutionary conservation of high-confidence RBP-binding motifs

(a) Flowchart showing the calculation of RBP conservation. (b) Scatter plot showing the correlation between the MuSIC-predicted accuracy and RBP conservation for RBPs from the 10 non-human species (line, the fitted linear regression line; error bands, 95% confidence interval; two-sided Pearson's correlation test). (c) Examples showing the consistency of the predicted binding motifs and experimentally-derived motifs. (d) Examples showing RBP-binding motifs across the 11 species, including human, orangutan, monkey, mouse, rat, chicken, frog, zebrafish, fly, *A. thaliana*, and yeast. Source data are provided as a Source Data file.

Figure S8

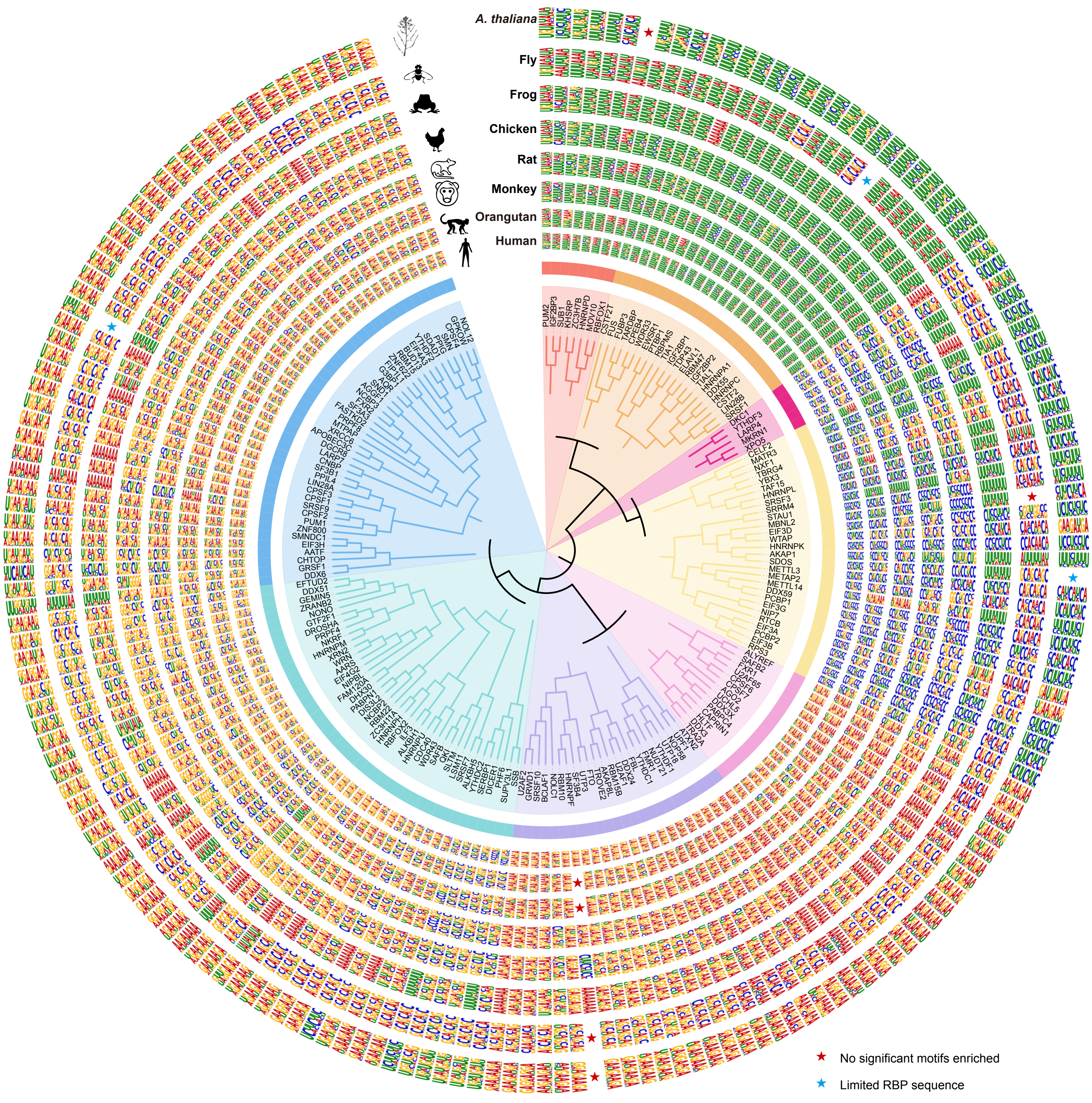

A. thaliana
Fly
Frog
Chicken
Rat
Monkey
Orangutan
Human
No significant motifs enriched
Limited RBP sequence

Supplementary Figure 8: Clustering of RBP-binding motifs

Hierarchical clustering of 184 predicted binding motifs in human, orangutan, monkey, rat, chicken, frog, fly, and yeast. Red stars indicate that there are no motifs enriched and blue stars indicate RBPs with limited sequence availability. Source data are provided as a Source Data file.

Figure S9

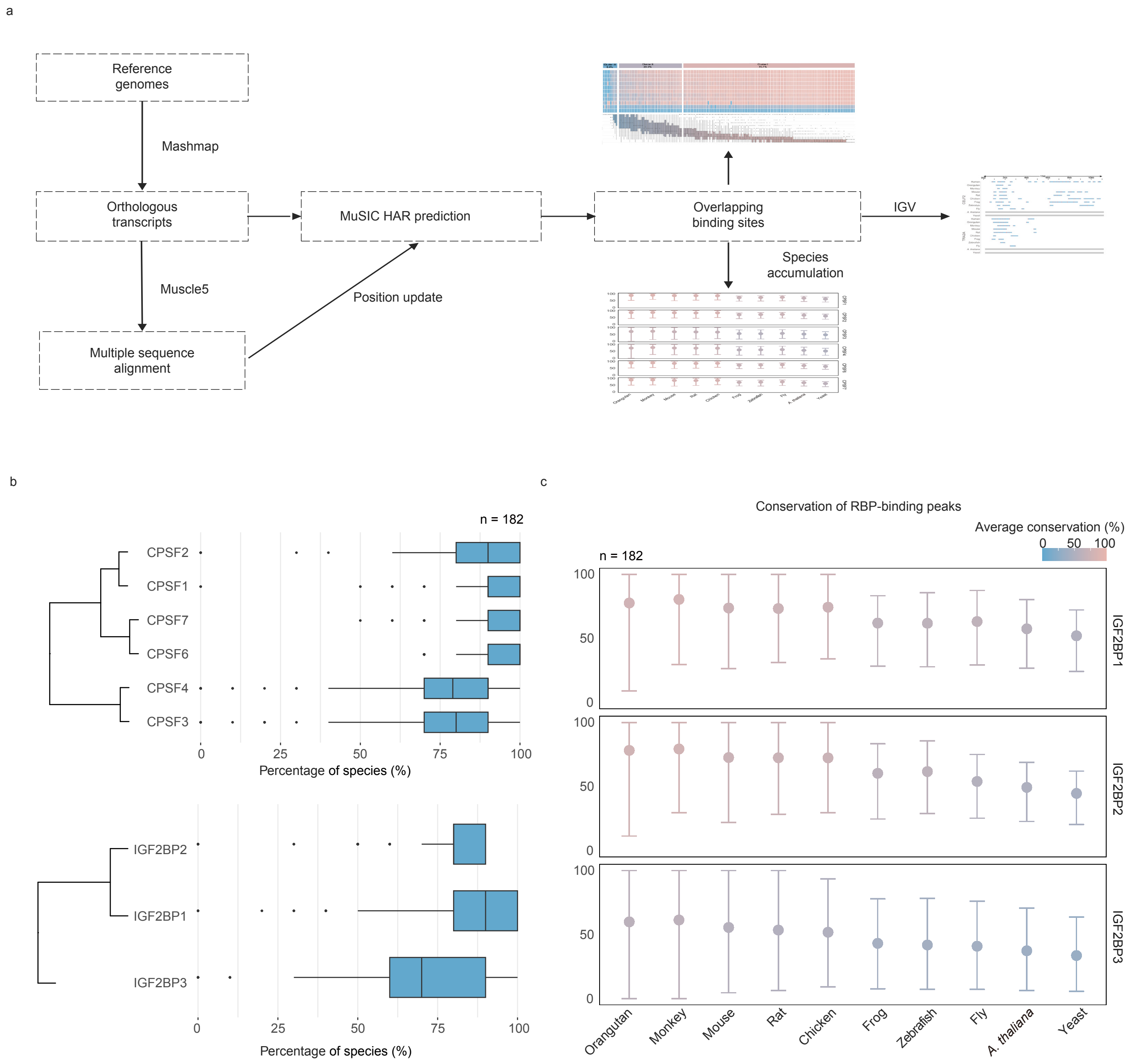
a
Reference genomes
Mashmap
Orthologous transcripts
Muscle5
Multiple sequence alignment
Position update
MuSIC HAR prediction
Overlapping binding sites
IGV
Species accumulation
b
n = 182
CPSF2
CPSF1
CPSF7
CPSF6
CPSF4
CPSF3
0
25
50
75
100
Percentage of species (%)
IGF2BP2
IGF2BP1
IGF2BP3
Percentage of species (%)
c
Conservation of RBP-binding peaks
Average conservation (%)
0 50 100
n = 182
IGF2BP1
IGF2BP2
IGF2BP3
Orangutan
Monkey
Mouse
Rat
Chicken
Frog
Zebrafish
Fly
A. thaliana
Yeast

Supplementary Figure 9: Cross-species conservation of predicted RBP-binding peaks

(a) Schematic showing the workflow for cross-species RBP-binding peaks conservation analysis. (b) Box plot showing the conservation degree of the 182 homologous transcripts containing the conserved peaks for CPSF family and IGF2BP family (n = 182 transcripts; center line, median; box limits, 25th and 75th percentiles; whiskers, 1.5 × interquartile range; points beyond the whiskers, outliers). (c) Forest plot showing the conservation of the peaks bound by the IGF2BP family across the 182 homologous transcripts. Points indicate the mean of all values. Error bars indicate the mean of the lowest 25% and the mean of the highest 25% of the ranked values (n = 182 transcripts). We include the predicted peaks ranging from human to each of the non-human species shown here according to the phylogenetic tree. Source data are provided as a Source Data file.

Figure S10

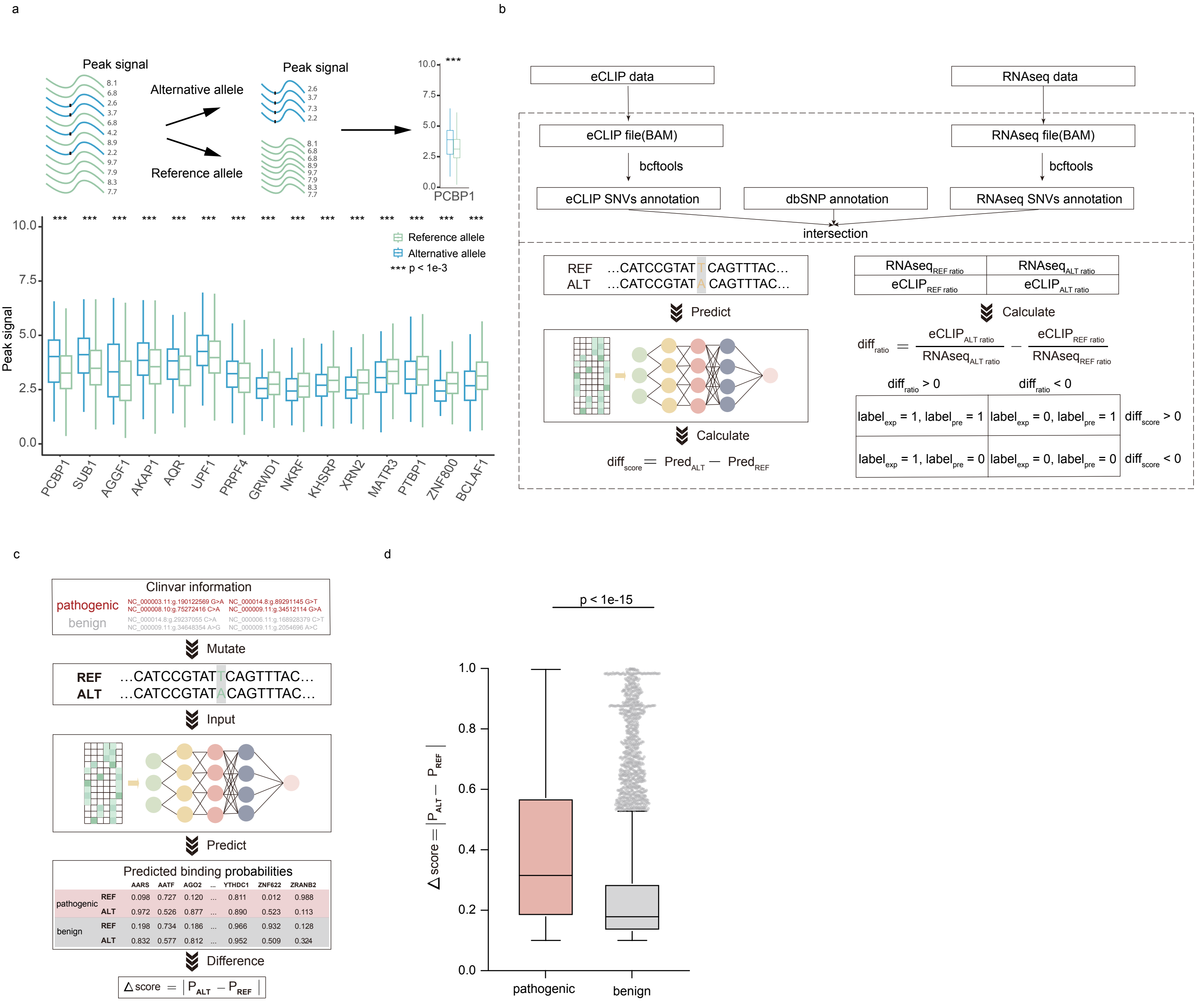

		AARS	AATF	AGO2	...	YTHDC1	ZNF622	ZRANB2
pathogenic	REF	0.098	0.727	0.120	...	0.811	0.012	0.988
	ALT	0.972	0.526	0.877	...	0.890	0.523	0.113
benign	REF	0.198	0.734	0.186	...	0.966	0.932	0.128
	ALT	0.832	0.577	0.812	...	0.952	0.509	0.324

Supplementary Figure 10: Consistency between the predicted and experimentally-derived effects of SNVs and the effects of pathogenic and benign SNVs on RBP binding

(a) Schematic showing the workflow for separating peak signals into the REF and ALT groups (center line, median; box limits, 25th and 75th percentiles; whiskers, 1.5 × interquartile range; two-sided Mann–Whitney U test). (b) Schematic showing the workflow for evaluating consistency of SNV effects between MuSIC predictions and experimental data. (c) Schematic showing the workflow for quantifying the effects of pathogenic and benign SNVs on RBP binding. (d) Box plot showing the effects of pathogenic (red) and benign (grey) SNVs on RBP–RNA binding affinity (center line, median; box limits, 25th and 75th percentiles; whiskers, 1.5 × interquartile range; points beyond the whiskers, outliers; two-sided Mann–Whitney U test). Source data are provided as a Source Data file.

Figure S11

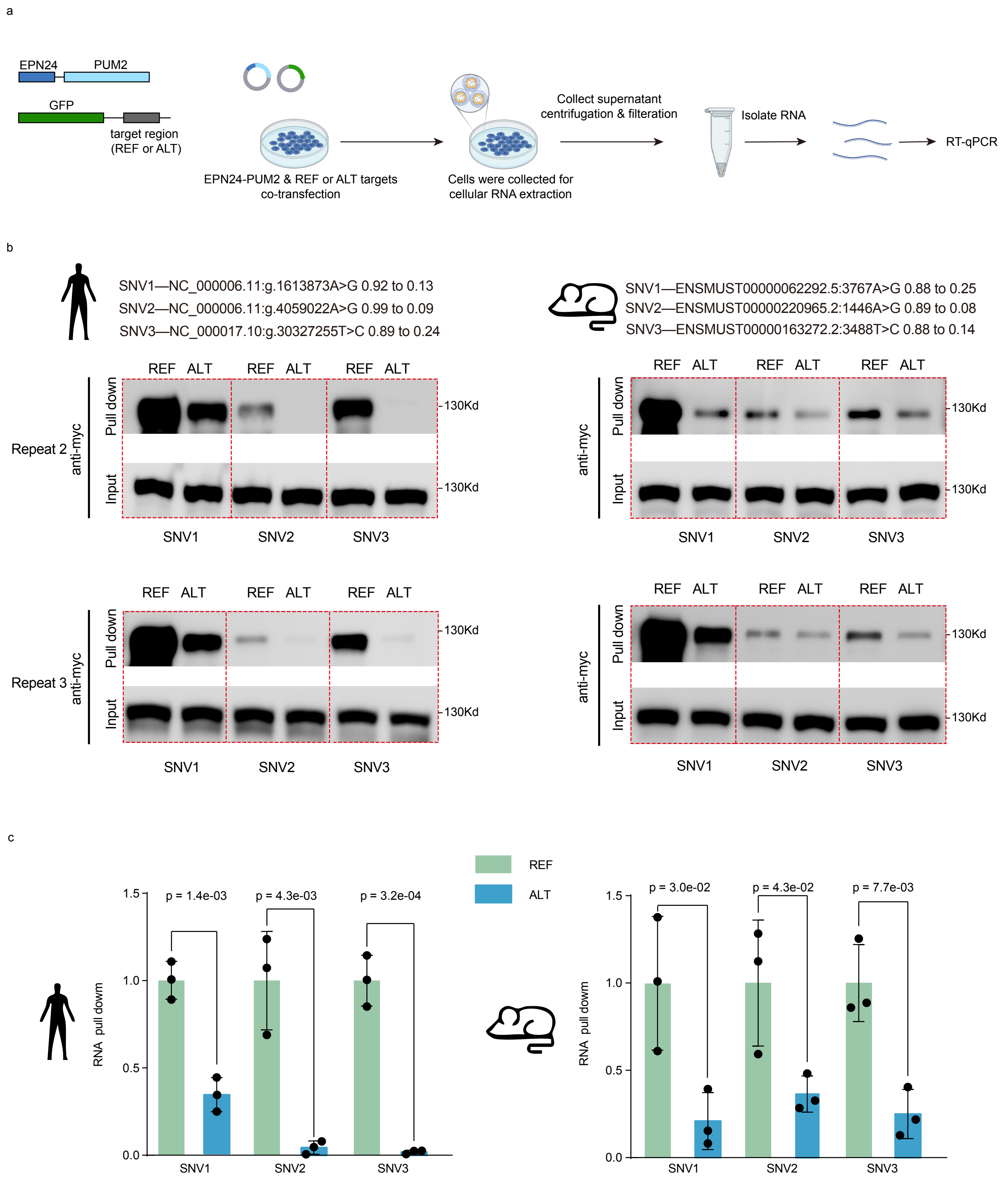

a
EPN24
PUM2
GFP
target region
(REF or ALT)
EPN24-PUM2 & REF or ALT targets
co-transfection
Cells were collected for
cellular RNA extraction
Collect supernatant
centrifugation & filteration
Isolate RNA
RT-qPCR
b
SNV1—NC_000006.11:g.1613873A>G 0.92 to 0.13
SNV2—NC_000006.11:g.4059022A>G 0.99 to 0.09
SNV3—NC_000017.10:g.30327255T>C 0.89 to 0.24
SNV1—ENSMUST00000062292.5:3767A>G 0.88 to 0.25
SNV2—ENSMUST00000220965.2:1446A>G 0.89 to 0.08
SNV3—ENSMUST00000163272.2:3488T>C 0.88 to 0.14
REF ALT REF ALT REF ALT
Repeat 2
anti-myc
Pull down
Input
130Kd
SNV1 SNV2 SNV3
Repeat 3
c
REF
ALT
RNA pull dowm
p = 1.4e-03
p = 4.3e-03
p = 3.2e-04
p = 3.0e-02
p = 4.3e-02
p = 7.7e-03
1.5
1.0
0.5
0.0
SNV1 SNV2 SNV3

Supplementary Figure 11: Experimental validations for the predicted effects of SNVs on RBP–RNA interactions in human and mouse

(a) Flowchart of using the POND method to detect the interaction between PUM2 and REF/ALT target RNA. PUM2 is fused to the C-terminus of the EPN24 nanocage monomer, while REF and ALT RNA targets are cloned into the 3'UTR of GFP on a separate plasmid. In HEK293T cells, EPN24-PUM2 is co-transfected with either GFP-REF or GFP-ALT RNA target plasmids. After transfection, EPN24-PUM2 packaged target RNAs into extracellular supernatant. Both supernatant and cells are collected for RNA extraction, followed by analysis of differential enrichment between REF and ALT target RNAs with RT-qPCR. (b) Examples showing in vitro RNA pull-down assays examining the effects of three homologous SNVs on PUM2 binding in human (left) and mouse (right). The upper blot represents the results of repeat 2, and the lower blot represents the results of repeat 3. (c) Bar plots showing *in vitro* RNA pull-down enrichment of human and mouse PUM2 protein binding to REF and ALT target RNAs. Green bars represent REF sequences, and blue bars represent ALT sequences. Data are presented as mean values +/- SD (n = 3 biologically independent experiments; two-sided unpaired Student's t-test). Source data are provided as a Source Data file.

Figure S12

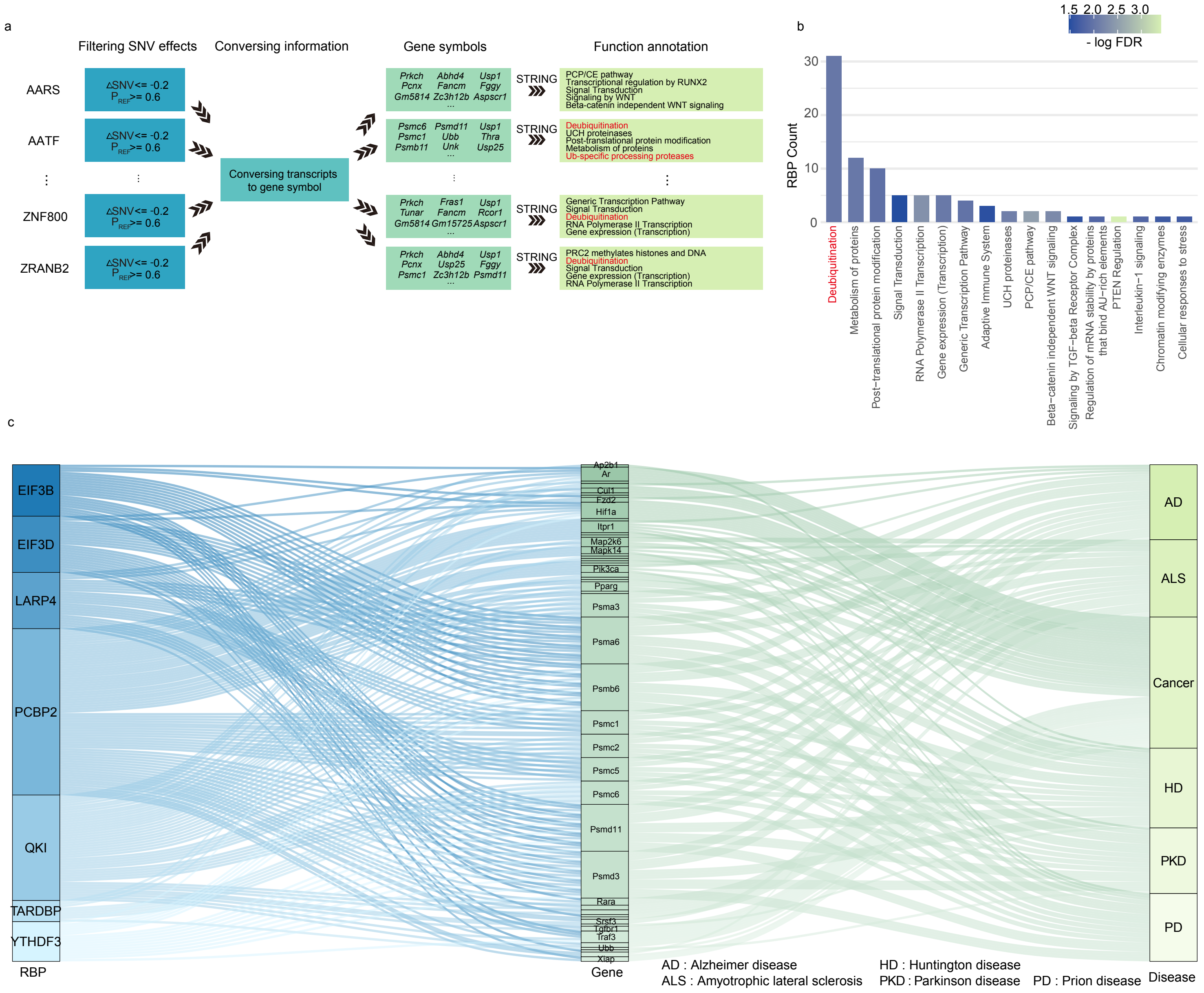
a
Filtering SNV effects
Conversing information
Gene symbols
Function annotation
AARS
AATF
ZNF800
ZRANB2
ΔSNV<= -0.2
P REF >= 0.6
Conversing transcripts to gene symbol
Prkch Abhd4 Usp1
Pcnx Fancm Fggy
Gm5814 Zc3h12b Aspscr1
Psmc6 Psmd11 Usp1
Psmc1 Ubb Thra
Psmb11 Unk Usp25
Prkch Fras1 Usp1
Tunar Fancm Rcor1
Gm5814 Gm15725 Aspscr1
Prkch Abhd4 Usp1
Pcnx Usp25 Fggy
Psmc1 Zc3h12b Psmd11
STRING
PCP/CE pathway
Transcriptional regulation by RUNX2
Signal Transduction
Signaling by WNT
Beta-catenin independent WNT signaling
Deubiquitination
UCH proteinases
Post-translational protein modification
Metabolism of proteins
Ub-specific processing proteases
Generic Transcription Pathway
Signal Transduction
Deubiquitination
RNA Polymerase II Transcription
Gene expression (Transcription)
PRC2 methylates histones and DNA
Deubiquitination
Signal Transduction
Gene expression (Transcription)
RNA Polymerase II Transcription
b
1.5 2.0 2.5 3.0
- log FDR
RBP Count
30
20
10
0
Deubiquitination
Metabolism of proteins
Post−translational protein modification
Signal Transduction
RNA Polymerase II Transcription
Gene expression (Transcription)
Generic Transcription Pathway
Adaptive Immune System
UCH proteinases
PCP/CE pathway
Beta−catenin independent WNT signaling
Signaling by TGF−beta Receptor Complex
Regulation of mRNA stability by proteins that bind AU−rich elements
PTEN Regulation
Interleukin−1 signaling
Chromatin modifying enzymes
Cellular responses to stress
c
EIF3B
EIF3D
LARP4
PCBP2
QKI
TARDBP
YTHDF3
RBP
Ap2b1
Ar
Cul1
Fzd2
Hif1a
Itpr1
Map2k6
Mapk14
Pik3ca
Pparg
Psma3
Psma6
Psmb6
Psmc1
Psmc2
Psmc5
Psmc6
Psmd11
Psmd3
Rara
Srsf3
Tgfbr1
Traf3
Ubb
Xiap
Gene
AD
ALS
Cancer
HD
PKD
PD
Disease
AD : Alzheimer disease
ALS : Amyotrophic lateral sclerosis
HD : Huntington disease
PKD : Parkinson disease
PD : Prion disease

Supplementary Figure 12: Predicted effects of SNVs on RBP–RNA interactions and their associations with human diseases

(a) Schematic showing the workflow of biological function enrichment analysis for RBPs affected by SNVs. (b) Bar chart showing the terms of biological functions associated with the weakly ubiquitin-related RBPs. (c) Sankey diagram showing the potential regulatory associations among RBPs, SNVs, and UPS-related disease. Source data are provided as a Source Data file.

Figure S13

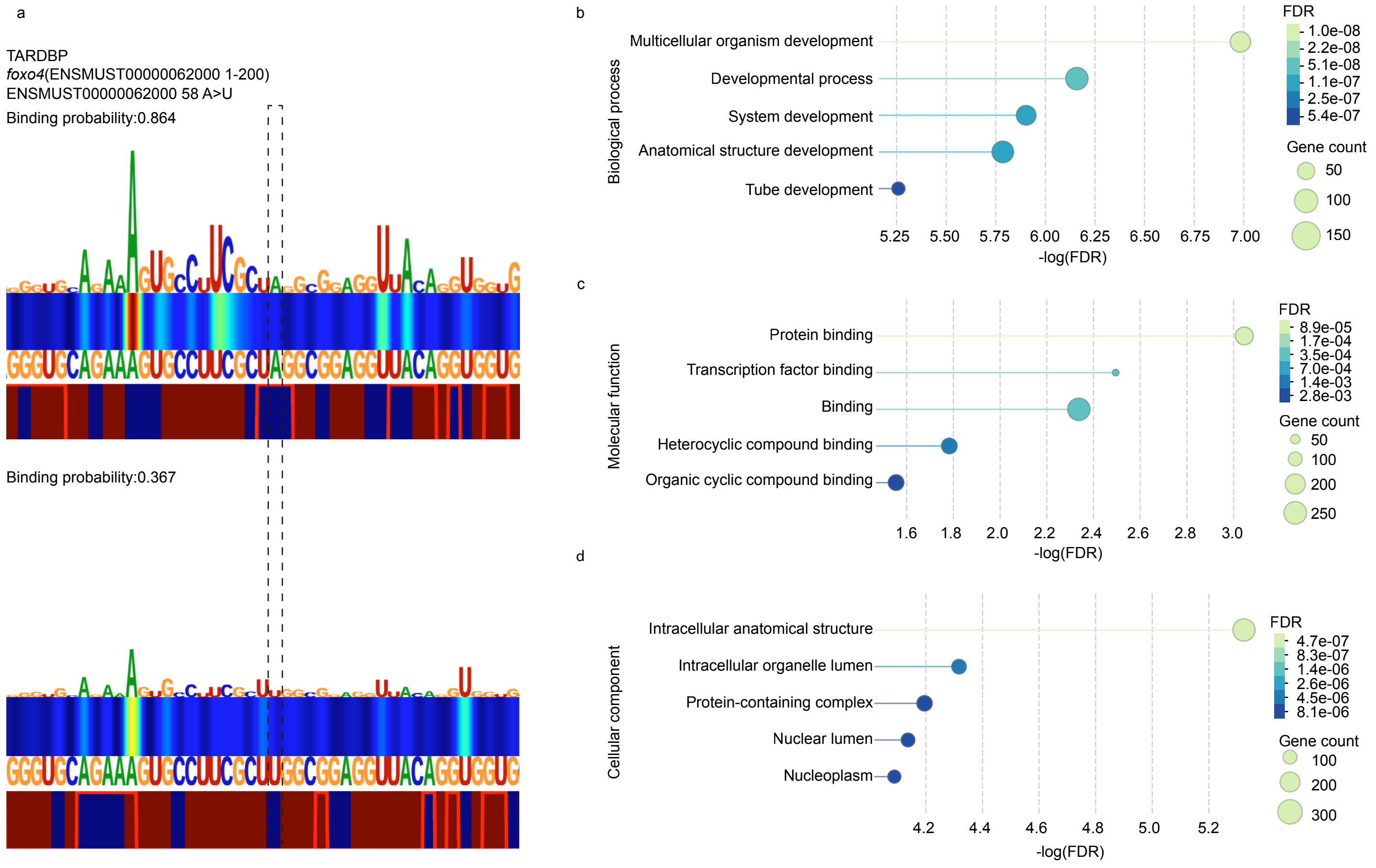

a
TARDBP
foxo4(ENSMUST00000062000 1-200)
ENSMUST00000062000 58 A>U
Binding probability:0.864
GGGUGCAGAAAGUGCCUUCGCUAGGCGGAGGUUACAGGUGGUG
Binding probability:0.367
GGGUGCAGAAAGUGCCUUCGCUUGGCGGAGGUUACAGGUGGUG
b
Biological process
Multicellular organism development
Developmental process
System development
Anatomical structure development
Tube development
5.25 5.50 5.75 6.00 6.25 6.50 6.75 7.00
-log(FDR)
FDR
1.0e-08
2.2e-08
5.1e-08
1.1e-07
2.5e-07
5.4e-07
Gene count
50
100
150
c
Molecular function
Protein binding
Transcription factor binding
Binding
Heterocyclic compound binding
Organic cyclic compound binding
1.6 1.8 2.0 2.2 2.4 2.6 2.8 3.0
-log(FDR)
FDR
8.9e-05
1.7e-04
3.5e-04
7.0e-04
1.4e-03
2.8e-03
Gene count
50
100
200
250
d
Cellular component
Intracellular anatomical structure
Intracellular organelle lumen
Protein-containing complex
Nuclear lumen
Nucleoplasm
4.2 4.4 4.6 4.8 5.0 5.2
-log(FDR)
FDR
4.7e-07
8.3e-07
1.4e-06
2.6e-06
4.5e-06
8.1e-06
Gene count
100
200
300

Supplementary Figure 13: Predicted effects of SNVs on TARDBP binding and the enriched biological functions of the transcripts containing the SNVs

(a) Example showing the effect of a homologous SNV (ENSMUST00000062000:58A>U) on TARDBP binding in mouse. The saliency maps show the predicted effect of REF (A) (top) and ALT (U) (bottom) on TARDBP binding. (b-d) Gene Ontology enrichment result showing the biological process, molecular function and cellular component pathways for the TARDBP-bound genes perturbed by SNVs. Source data are provided as a Source Data file.
