# Supplementary material for: Augmented prediction of multi-species protein–RNA interactions using evolutionary conservation of RNA-binding proteins
Source: Nat Commun. 2026 Apr 27;17:5764. doi: 10.1038/s41467-026-72351-6 (PMC13324433; doi:10.1038/s41467-026-72351-6)
Supplement: Supplementary file 2 — Description of Additional Supplementary Files [file 41467_2026_72351_MOESM2_ESM.pdf]

Description of Additional supplementary files

Supplementary Data 1: Evolutionary conservation of RBPs across 11 species

Supplementary Data 2: Conservationbased grouping of 216 RBPs

Supplementary Data 3: Ablation experiments of MuSIC

Supplementary Data 4: Prediction performance of MuSIC

Supplementary Data 5: Predicted RBPbinding motifs from MuSIC

Supplementary Data 6: Consistency of SNV effects

Supplementary Data 7: Experimental validation of SNV effects Supplementary Data 8: Functional enrichment of SNV-affected genes
