# Supplementary material for: Augmented prediction of multi-species protein–RNA interactions using evolutionary conservation of RNA-binding proteins
Source: Nat Commun. 2026 Apr 27;17:5764. doi: 10.1038/s41467-026-72351-6 (PMC13324433; doi:10.1038/s41467-026-72351-6)
Supplement: Supplementary file 11 — Reporting Summary [file 41467_2026_72351_MOESM11_ESM.pdf]

nature portfolio

Corresponding author(s): Prof Lei Sun, Prof Yucheng T. Yang

Last updated by author(s): Apr 2, 2026

Reporting Summary

Nature Portfolio wishes to improve the reproducibility of the work that we publish. This form provides structure for consistency and transparency in reporting. For further information on Nature Portfolio policies, see our Editorial Policies and the Editorial Policy Checklist.

Statistics

For all statistical analyses, confirm that the following items are present in the figure legend, table legend, main text, or Methods section.

n/a	Confirmed	
☐	☒	The exact sample size (*n*) for each experimental group/condition, given as a discrete number and unit of measurement
☐	☒	A statement on whether measurements were taken from distinct samples or whether the same sample was measured repeatedly
☐	☒	The statistical test(s) used AND whether they are one- or two-sided<br>*Only common tests should be described solely by name; describe more complex techniques in the Methods section.*
☐	☒	A description of all covariates tested
☐	☒	A description of any assumptions or corrections, such as tests of normality and adjustment for multiple comparisons
☐	☒	A full description of the statistical parameters including central tendency (e.g. means) or other basic estimates (e.g. regression coefficient) AND variation (e.g. standard deviation) or associated estimates of uncertainty (e.g. confidence intervals)
☐	☒	For null hypothesis testing, the test statistic (e.g. *F*, *t*, *r*) with confidence intervals, effect sizes, degrees of freedom and *P* value noted<br>*Give P values as exact values whenever suitable.*
☒	☐	For Bayesian analysis, information on the choice of priors and Markov chain Monte Carlo settings
☒	☐	For hierarchical and complex designs, identification of the appropriate level for tests and full reporting of outcomes
☐	☒	Estimates of effect sizes (e.g. Cohen's *d*, Pearson's *r*), indicating how they were calculated

Our web collection on statistics for biologists contains articles on many of the points above.

Software and code

Policy information about availability of computer code

Data collection	All data collection for this study was performed using the Python language (v3.11.0). We did not use any commercial software for data collection in this study.
Data analysis	MEGA (v11.0.13), ClustalX (v2.1.1), US-align (https://github.com/pylelab/USalign), PyMOL (v3.1.0), AlphaFold2 (https://github.com/google-deepmind/alphafold), AlphaFold3 webserver(https://alphafoldserver.com/), GFF Annotation Parser (GAP) (https://github.com/lipan6461188/GAP), MuSIC (v1.0.1) (https://github.com/GALE1228/MuSIC_pretrain), PaRPI(https://github.com/ljquanlab/PaRPI),HDRNet (https://github.com/zhuhr213/HDRNet), PrismNet (https://github.com/kuixu/PrismNet), DeepBind (https://github.com/jisraeli/DeepBind), and GraphProt (https://github.com/dmaticzka/GraphProt), MEME (v5.5.7), itol.toolkit package (v1.1.10), Mashmap (v3.1.3), Muscle5 (v5.1), IGV (v2.16.2), BCFtools (v1.20), BEDTools (v2.27.1), STRING website (https://string-db.org/).

For manuscripts utilizing custom algorithms or software that are central to the research but not yet described in published literature, software must be made available to editors and reviewers. We strongly encourage code deposition in a community repository (e.g. GitHub). See the Nature Portfolio guidelines for submitting code & software for further information.

Data

Policy information about availability of data

All manuscripts must include a data availability statement. This statement should provide the following information, where applicable:

- Accession codes, unique identifiers, or web links for publicly available datasets
- A description of any restrictions on data availability
- For clinical datasets or third party data, please ensure that the statement adheres to our policy

The 216 RBP sequences for the 11 species used in this study were downloaded from UniProt database (https://www.uniprot.org/). The corresponding entry IDs are listed in Supplementary Data 1 file.
The 18S rRNA reference sequences for the 11 species were downloaded from RNAcentral database under the following accession IDs: Homo sapiens (https://rnacentral.org/search?q=URS0000726FAB_9606), Pongo abelii (https://rnacentral.org/search?q=URS000302FF38_9601), Macaca fascicularis (https://rnacentral.org/search?q=URS00005800AF_1035826), Mus musculus (https://rnacentral.org/search?q=URS00005B0A54_10090), Rattus norvegicus (https://rnacentral.org/search?q=URS0002A14804_10116), Gallus gallus (https://rnacentral.org/search?q=URS000263BE37_9031), Xenopus laevis (https://rnacentral.org/search?q=URS0002349D5A_8355), Danio rerio (https://rnacentral.org/search?q=URS0000668FC4_7955), Drosophila melanogaster (https://rnacentral.org/search?q=URS0000A2DABB_7227), Arabidopsis thaliana (https://rnacentral.org/search?q=URS00021C577A_3702), and Saccharomyces cerevisiae (https://rnacentral.org/search?q=URS0000B21DE0_559292).
The 3D structures of RBP-RNA interactions were obtained from the Protein Data Bank (PDB) with the following entry IDs: Human: 4QIL (https://www.rcsb.org/structure/4QIL) and Mouse: 4QI2 (https://www.rcsb.org/structure/4QI2).
The CLIP datasets used for model training, validation, and RNA pattern conservation analysis is available in POSTAR3 database (https://cloud.tsinghua.edu.cn/d/8133e49661e24ef7a915/)
The fly RBP-binding datasets used for model training and validation is available in ENCODE database under accession code ENCSR432JLI (https://www.encodeproject.org/experiments/ENCSR432JLI/).
The eCLIP data for 103 RBPs used in the SNV impact analysis was downloaded from the ENCODE database under the accession number ENCSR456FVU (https://www.encodeproject.org/publication-data/ENCSR456FVU/).
The smartSHAP sequencing dataset, used as background data, is available under the GEO accession number GSE14580530 (https://www.ncbi.nlm.nih.gov/geo/query/acc.cgi?acc=GSE145805).
The foundation models and pre-trained weights were obtained from the following publicly available repositories: RNA-FM (https://github.com/ml4bio/RNA-FM), RiNALMo (https://github.com/lbcb-sci/RiNALMo), ProtT5 (https://github.com/agemagician/ProtTrans).
All oligonucleotides used for experimental validation in this study, including primers and RNA sequences, as well as the RBP sequences and plasmids, are provided in Supplementary Data 5 and 7 and the Source Data file. Source data are provided with this paper.

Research involving human participants, their data, or biological material

Policy information about studies with human participants or human data. See also policy information about sex, gender (identity/presentation), and sexual orientation and race, ethnicity and racism.

Reporting on sex and gender	N/A. This work does not involve human research participants
Reporting on race, ethnicity, or other socially relevant groupings	N/A. This work does not involve human research participants
Population characteristics	N/A. This work does not involve human research participants
Recruitment	N/A. This work does not involve human research participants
Ethics oversight	N/A. This work does not involve human research participants

Note that full information on the approval of the study protocol must also be provided in the manuscript.

Field-specific reporting

Please select the one below that is the best fit for your research. If you are not sure, read the appropriate sections before making your selection.

☒ Life sciences ☐ Behavioural & social sciences ☐ Ecological, evolutionary & environmental sciences

For a reference copy of the document with all sections, see nature.com/documents/nr-reporting-summary-flat.pdf

Life sciences study design

All studies must disclose on these points even when the disclosure is negative.

Sample size	No statistical methods were used to predetermine the sample size. All sample sizes were determined based on prior experience with similar experiments and literature reports (Sun et al., Cell Res, 2021, PMID: 33623109; Zhu et al., Nat Commun, 2023, PMID: 37884495).
Data exclusions	During CLIP-seq data processing, we excluded peak segments shorter than 8nt from the positive datasets to prevent short fragments from impacting model performance. No other exclusions were made.

Replication	The results were confirmed in three biological replicates for each experiment, both in vivo and in vitro, unless otherwise stated. All attempts to replicate the data were successful.
Randomization	The datasets were randomly split into training, validation, and test sets, with the use of random seeds described in the Methods section (Compiling RBP-binding peak datasets). No manual sample assignment was performed.
Blinding	Blinding was not applicable because the focus of this study was on the development of a new method to predict cross-species RBP-RNA interactions, which did not involve group allocation or blinding.

Reporting for specific materials, systems and methods

We require information from authors about some types of materials, experimental systems and methods used in many studies. Here, indicate whether each material, system or method listed is relevant to your study. If you are not sure if a list item applies to your research, read the appropriate section before selecting a response.

Materials & experimental systems

n/a	Involved in the study
☐	☒ Antibodies
☐	☒ Eukaryotic cell lines
☒	☐ Palaeontology and archaeology
☒	☐ Animals and other organisms
☒	☐ Clinical data
☒	☐ Dual use research of concern
☒	☐ Plants

Methods

n/a	Involved in the study
☒	☐ ChIP-seq
☒	☐ Flow cytometry
☒	☐ MRI-based neuroimaging

Antibodies

Antibodies used	Anti-myc tag (HRP Conjugated) (Beyotime, catalog number AF2867, 1:1000) ; Anti-β-actin antibody (CST, 4970, 1:10,000); HRP-conjugated secondary antibodies (Beyotime, A0208, 1:1000)
Validation	Anti-myc tag (HRP Conjugated) : https://www.beyotime.com/product/AF2867-200%CE%BCl.htm<br><br>Anti-β-actin antibody : https://www.cellsignal.com/products/primary-antibodies/beta-actin-13e5-rabbit-monoclonal-antibody/4970?srsltid=AfmBOoppnfSzT-99Sl94fswFYKn5m6bVQHoLmghoP0-koLKYhqXXHiKE<br><br>HRP-conjugated secondary antibodies : https://www.beyotime.com/product/A0208.htm

Eukaryotic cell lines

Policy information about cell lines and Sex and Gender in Research

Cell line source(s)	HEK293T (ATCC, CRL-3216) cells were used in this study.
Authentication	HEK293T cells were obtained from ATCC (CRL-3216). Authentication information was based on ATCC vendor documentation (https://www.atcc.org/products/crl-3216).
Mycoplasma contamination	It was confirmed that the HEK293T cells were tested negative for mycoplasma contamination.
Commonly misidentified lines<br>(See ICLAC register)	No commonly misidentified lines was used.

Plants

Seed stocks	n/a
Novel plant genotypes	n/a
Authentication	n/a
