# Supplementary material for: Augmented prediction of multi-species protein–RNA interactions using evolutionary conservation of RNA-binding proteins
Source: Nat Commun. 2026 Apr 27;17:5764. doi: 10.1038/s41467-026-72351-6 (PMC13324433; doi:10.1038/s41467-026-72351-6)
Supplement: Supplementary file 12 — Transparent Peer Review file [file 41467_2026_72351_MOESM12_ESM.pdf]

nature portfolio

Peer Review File

Augmented prediction of multi-species protein–RNA interactions using evolutionary conservation of RNA-binding proteins

Corresponding Author: Professor Lei Sun

This file contains all reviewer reports in order by version, followed by all author rebuttals in order by version.

Version 0:

Reviewer comments:

Reviewer #1

(Remarks to the Author)
This manuscript presents MuSIC, a deep learning framework for predicting cross-species RNA-binding protein (RBP) interactions by leveraging evolutionary conservation and label smoothing techniques. The study addresses a critical gap in computational biology by enabling accurate RBP-RNA interaction prediction across multiple species, including those with limited experimental data. The authors provide a comprehensive evaluation of MuSIC's performance, demonstrating its superiority over existing methods and its utility in motif discovery and variant impact analysis. While the manuscript is well-structured and the results are promising, several areas could benefit from further clarification, expanded analysis, or methodological refinement.
Major:
1. The ablation study on input sequence length (100, 200, 400 nt) provides useful insights into model performance. However, the range of tested lengths remains relatively narrow and may not fully capture the impact of longer contextual windows on cross-species generalization. I recommend extending the analysis to include additional lengths.
2. The use of RNA structure: I found the ablation results on RNA structure quite compelling. I'm curious whether the performance gain is due to biologically meaningful structure information or if it acts more like a regularizing feature. Have you considered testing with randomized RNA structures of the same length or letting the model learn structure representations directly from sequence? I wonder how much of the gain is attributable to true structural signal, also why the experimental structures do not improve the performances.
3. In Supplementary Fig. 3d, the t-SNE plots suggest improved separation between positive and negative samples after adding SE and residual blocks. However, the SE model vs Input, and the Residual vs FCL does not clearly show the difference. Have you considered quantifying this improvement using metrics? It would be helpful to understand how much the feature space becomes more discriminative beyond visual inspection.
4. Although MuSIC demonstrates superior performance over existing methods, the evaluation is limited to a relatively small number of RBPs (14 for cross-species tasks). This narrow scope may limit the generalizability of the findings. Expanding the benchmark to include more diverse RBPs and species would enhance the credibility of the performance claims.
5. While motif rediscovery in non-human species is promising, the validation of predicted motifs relies heavily on known motifs from databases. This may bias the evaluation toward well-characterized RBPs. Incorporating de novo motif discovery validation or experimental confirmation for novel motifs would provide stronger evidence of MuSIC's generalizability.
6. While MuSIC demonstrates strong performance for RBPs with well-characterized binding profiles and conservation data, it remains unclear how the model handles RBPs with limited or ambiguous training data—particularly those lacking annotated RBDs or CLIP-seq datasets. This is especially relevant for de novo RBD discovery, where no prior binding motifs or structural templates exist.
7. The SNV impact analysis is a valuable application, yet the validation appears limited to a few illustrative examples. A more systematic evaluation—such as comparing MuSIC predictions with high-throughput mutagenesis or disease variant databases—would better demonstrate the model's utility in variant interpretation.
Minor: The figure in Supp Table 2 about the definition of different conserved level is confusing.

(Remarks on code availability)

Reviewer #2

(Remarks to the Author)
I co-reviewed this manuscript with one of the reviewers who provided the listed reports. This is part of the Nature Communications initiative to facilitate training in peer review and to provide appropriate recognition for Early Career Researchers who co-review manuscripts.

(Remarks on code availability)

Reviewer #3

(Remarks to the Author)
This paper introduces MuSIC (Multi-Species RBP–RNA Interactions using Conservation), a deep learning framework for predicting RNA-binding protein (RBP)–RNA interactions across multiple species. The key contributions are as follows:

1. Cross-species prediction: MuSIC predicts RBP–RNA interactions across 11 diverse species, from human to yeast.
2. Conservation-Guided Label Smoothing: The model integrates multi-level RBP conservation patterns (sequence and structure) into a conservation-weighted label smoothing scheme and employs gradient weight adaptation to dynamically balance loss components during training.
3. Variant effect prediction: MuSIC quantifies the impact of homologous single-nucleotide variants (SNVs) on RBP binding across species, with experimental validation in mouse models.

While the work is well executed and biologically grounded, it appears to be incremental rather than groundbreaking. Several models already address cross-species RBP prediction—such as PaRPI (which integrates cross-protocol and cross-batch datasets) and RBPsuite 2.0 (which achieves wide species and protein coverage using deep learning)—yet these are not included in the comparison, weakening the claim of state-of-the-art performance.

Moreover, MuSIC’s cross-species performance gains are primarily limited to closely related species (e.g., human→mouse). Prediction accuracy drops markedly for distant taxa (e.g., fly, yeast), indicating restricted generalization beyond highly conserved domains.

The label smoothing strategy, though effective, is conceptually incremental; applying conservation-based weighting is interesting but not a major methodological advance. Furthermore, the paper does not compare its approach against alternative domain adaptation or transfer learning frameworks (e.g., adversarial alignment, contrastive pretraining, or large pretrained RNA foundation models). Architecturally, the model design (CNN, residual, SE modules) is standard and similar to existing frameworks such as PrismNet, HDRNet, and DeepCLIP.

In summary, MuSIC provides a solid, technically competent framework that meaningfully integrates evolutionary conservation into RBP–RNA prediction and demonstrates credible cross-species results. However, the methodological innovation is modest, and the lack of comprehensive benchmarking against other cross-species or domain adaptation approaches limits its impact. The study would benefit from exploring stronger transfer-learning strategies for modeling RBP conservation patterns. Given these issues, I do not recommend acceptance.

(Remarks on code availability)

Reviewer #4

(Remarks to the Author)
Accurately modeling RNA–protein interactions remains a central challenge in computational genomics. Reliable prediction of RBP binding is essential for understanding post-transcriptional regulation and the impact of non-coding variants. He et al propose a new deep learning based model to predict RBP-RNA interactions across species. Their model is based on a CNN which utilizes conservation-based label smoothing. They perform evaluations that include within-species binding prediction, cross-species binding prediction as well as SNV effect prediction. The method is well-explained and the figures are clear.

The authors first demonstrate that evolutionarily conserved RBPs exhibit similar RNA-binding preferences. While this analysis is useful, it is somewhat repetitive, as previous studies have already reported similar patterns (e.g., Ray et al., 2013).

Regarding the performance of MuSIC, it’s important to note that MuSIC’s within-species prediction is only slightly better than HDRNet and PrismNet (0.88 vs 0.87 and 0.86). MuSIC’s performance in cross-species prediction of binding is much superior than other methods. However, a key concern here is that the model’s higher performance in closely related species may be largely driven by trivial sequence homology, not true cross-species generalizability. Because conservation signals are explicitly incorporated into the model's label smoothing strategy, the reported improvements in closely related species may reflect circular reasoning. The model is evaluated on its ability to recover conserved binding sites, but conservation

itself is already provided as an input. The authors should eloborate on this. How would a simple baseline scanning model using the same motif (as PWM or PFM9) weighted by conservation values score?

The authors have to explain the effect of sequence length on performance. How is it possible that using ~100nt and ~400 nt as input sequence length can give random prediction results (i.e. AUCs close to 0.5)? If structure features were included one could argue that it's due to the local folding of RNA molecule, but here it is not the case.

In rediscovering the peaks from CLIP-seq experiments (Pg 11), 0.9 threshold is used to classify a prediction as a hit. This threshold is chosen in an ad-hoc manner and may not be an optimal threshold value for other methods. It would be interesting to see how the other methods perform when the top X ranked predictions are compared to experimentally derived peaks where X is a fixed number.

It could be valuable to have similar GC-content in positive and negative sequences. Can the authors comment on whether they take into account this?

In the cross-species prediction task, it is unclear why the authors use a 4:1 split between training and validation. Since the training and test datasets come from entirely separate species (e.g., human vs. mouse), this is not a traditional cross-validation setting. It is therefore unclear why the authors subsample the mouse AGO2 iCLIP dataset to create a test set with only 34 positive and 60 negative sequences. A more straightforward approach would be to use the entire mouse dataset as the test set. At minimum, if subsampling is necessary, the authors should repeat the procedure multiple times with different random seeds and report the average and variability of the results to ensure robustness.

Minor comments:

Line 336.
"Notably, RBP-binding motif prediction failed for certain RBPs due to their weak or undetectable binding signals in the distantly related species, such as yeast, fly, and zebrafish. " It would be valuable to list those within the sentence.

Line 300. "evolutionarily closed" -> "evolutionarily close"

Line 308. The word "quantificationally" should be replaced with some other word
Figure S7 -> legnth

(Remarks on code availability)
The authors provide fully accessible code for all analyses, accompanied by rigorous and detailed documentation.

Version 1:

Reviewer comments:

Reviewer #1

(Remarks to the Author)
The authors have thoroughly and constructively addressed each of my points with new experiments, rigorous quantitative analyses, and experimental validation. The expanded sequencelength ablation, inclusion of structure controls and foundationmodel embeddings, and the use of FID to quantify feature separability substantially deepen the study. The broadened crossspecies benchmarking and the experimental validation of de novo motifs further support the model's generalizability. These revisions have materially strengthened the manuscript, and I recommend acceptance.

(Remarks on code availability)

Reviewer #2

(Remarks to the Author)
I co-reviewed this manuscript with one of the reviewers who provided the listed reports. This is part of the Nature Communications initiative to facilitate training in peer review and to provide appropriate recognition for Early Career Researchers who co-review manuscripts.

(Remarks on code availability)

Reviewer #3

(Remarks to the Author)
I have no further comments.

(Remarks on code availability)

Reviewer #4

(Remarks to the Author)
The authors have addressed all my concerns. I think the study is acceptable in its current form.

(Remarks on code availability)
The authors provide fully accessible code for all analyses, accompanied by rigorous and detailed documentation.

Open Access This Peer Review File is licensed under a Creative Commons Attribution 4.0 International License, which permits use, sharing, adaptation, distribution and reproduction in any medium or format, as long as you give appropriate credit to the original author(s) and the source, provide a link to the Creative Commons license, and indicate if changes were made.
In cases where reviewers are anonymous, credit should be given to 'Anonymous Referee' and the source.
The images or other third party material in this Peer Review File are included in the article's Creative Commons license, unless indicated otherwise in a credit line to the material. If material is not included in the article's Creative Commons license and your intended use is not permitted by statutory regulation or exceeds the permitted use, you will need to obtain permission directly from the copyright holder.
To view a copy of this license, visit https://creativecommons.org/licenses/by/4.0/

Response Letter to

Augmented prediction of multi-species protein–RNA interactions using evolutionary conservation of RNA-binding proteins

Point-to-Point response to Reviewer #1

Reviewer #1: This manuscript presents MuSIC, a deep learning framework for predicting cross-species RNA-binding protein (RBP) interactions by leveraging evolutionary conservation and label smoothing techniques. The study addresses a critical gap in computational biology by enabling accurate RBP-RNA interaction prediction across multiple species, including those with limited experimental data. The authors provide a comprehensive evaluation of MuSIC's performance, demonstrating its superiority over existing methods and its utility in motif discovery and variant impact analysis. While the manuscript is well-structured and the results are promising, several areas could benefit from further clarification, expanded analysis, or methodological refinement.

RESPONSE:

Thanks for your positive and constructive evaluation of our work. In brief, MuSIC addresses a key challenge in the cross-species RBP–RNA interaction prediction using conservation information and label smoothing techniques, and achieves improved accuracy and generalization across species compared to existing tools. We performed extensive prediction performance evaluations and experimental validations, and demonstrated that MuSIC could consistently outperform existing computational tools in cross-species tasks, and capture biologically meaningful binding patterns, as supported by the motif discovery and variant impact analyses.

MuSIC was developed to highlight the transferable features rather than the species-specific noise in RBP–RNA interactions, although cross-species prediction can inevitably show the evolutionary and regulatory differences of RBP binding between species. In response to your suggestions, we have performed additional analyses, including testing a range of sequence lengths, incorporating foundation models for RNA structural feature extraction, assessing feature-space discriminability using the Fréchet Inception Distance (FID), expanding the datasets from *Drosophila melanogaster* (fly), conducting large-scale analysis of ClinVar genetic variants, and experimentally confirming novel motifs. We have also carefully revised the manuscript, with a point-by-point response attached.

Reviewer #1 comment 1

The ablation study on input sequence length (100, 200, 400 nt) provides useful insights into model performance. However, the range of tested lengths remains relatively narrow and may not fully capture the impact of longer contextual windows on cross-species generalization. I recommend extending the analysis to include additional lengths.

RESPONSE:

Thanks for pointing out the importance of contextual window size in the cross-species generalization. In response, we extended the ablation analysis to cover a broader range of input sequence length (i.e., 101, 200, 300, 400, 500, 1000, and 2000nt), allowing to assess the effects of sequence length on cross-species performance in a more comprehensive manner.

We found that, in the cross-species setting, MuSIC could consistently achieve the best prediction performance under the input length of 200nt (**Fig. R1a**). Notably, extending the contextual window size did not improve the prediction performance, instead leading to declined prediction performance. We also performed the same analysis in the within-species setting, and found that MuSIC could achieve the best prediction performance under the input length of 101nt, while 200nt showing a comparable prediction performance (**Fig. R1b**). Given the primary objective of addressing the cross-species generalization issue, we selected 200nt as the default input sequence length in MuSIC.

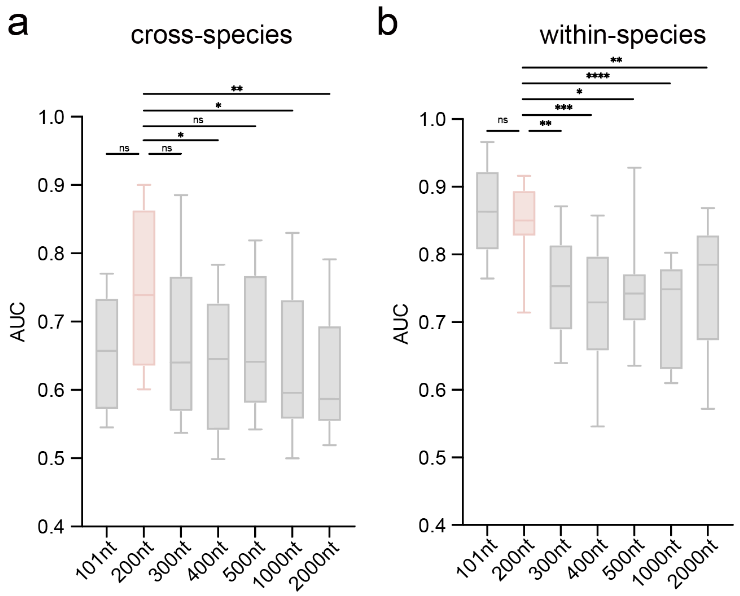

Figure R1 Performance comparison across different input sequence lengths.

Boxplot showing the performance of MuSIC using different input sequence lengths in (a) cross-species and (b) within-species tasks. ns $P > 0.05$, * $P < 0.05$, ** $P < 0.01$, *** $P < 0.005$, **** $P < 0.001$, Wilcoxon test.

Excerpt (main text)

We replaced **Fig. R1a** with **Supplementary Fig. 3c** and **Fig. R1b** with **Supplementary Fig. 3d.** And we added the relevant descriptions to the **Results** (L242 – L251), the **Discussion** (L532 – L538) and the **Methods** (L891 – L893) sections:

Results (L242 – L251):

> We found that increasing the input sequence length to 200nt could significantly improve the Area Under the Curve (AUC) score, reaching 0.75 in cross-species prediction compared to sequences of lengths 101nt, 300nt, 400nt, 500nt, 1000nt, and 2000nt (**Fig. 2f**, **Supplementary Fig. 3c** and **Supplementary Table 3**). We also performed the same analysis in the within-species setting, and found that MuSIC could achieve the best prediction performance under the input length of 101nt, while 200nt showing a comparable prediction performance (**Supplementary Fig. 3d**). Given the primary objective of addressing the cross-species generalization issue, we selected 200nt as the default input sequence length in MuSIC.

Discussion (L532 – L538):

> In our study, we revealed that increasing the input sequence length from 101nt to 200nt could significantly improve the prediction performance across species (AUC from 0.65 to 0.75). In contrast, further increasing the sequence length beyond 200nt, including 300nt, 400nt, 500nt, 1000nt, and 2000nt, led to a consistent decline in model performance. Increasing the input sequence length can not only provide more useful sequence information, but also may include noisy and redundant sequence information for modeling RBP binding.

Methods (L891 – L893):

> (i) input sequence length: we tried different input sequence lengths of 101nt, 200nt, 300nt, 400nt, 500nt, 1000nt and 2000nt.

Reviewer #1 comment 2

The use of RNA structure: I found the ablation results on RNA structure quite compelling. I'm curious whether the performance gain is due to biologically meaningful structure information or if it acts more like a regularizing feature. Have you considered testing with randomized RNA structures of the same length or letting the model learn structure representations directly from sequence? I wonder how much of the gain is attributable to true structural signal, also why the experimental structures do not improve the performances.

RESPONSE:

Thanks for this insightful suggestion regarding the contribution of RNA structural information. It is necessary to disentangle whether the observed performance gains arise from biologically meaningful structural information or just regularization effect. In response, we conducted additional control experiments in which RNA structures were randomized while preserving the original sequence length. As expected, the prediction performance exhibited mild improvement when using the predicted RNA structures, indicating the value of biologically informative structural patterns (**Fig. R2, boxes 3 and 4**). We also noted that experimentally derived RNA structures did not lead to obvious performance improvements (**Fig. R2, boxes 4 and 5**). We anticipate that this is due to the limited transcriptome-wide signal coverage, experimental noise, and strong species-specificity signal bias in these structural probing datasets. The predicted RNA structures can generate uniform transcriptome-wide signal coverage, as well as consistent signal resolution across different species, which are essential for the cross-species modeling in MuSIC.

We sincerely appreciate and follow your suggestion to explore how the inclusion of RNA secondary structure information from the RNA foundation models can augment the prediction performance of MuSIC. Existing RNA foundation models, such as RNA-FM and RiNALMo, were trained based on large-scale RNA datasets (e.g., RNAcentral) containing conserved RNA structures. Thus, we anticipate that the potential RNA secondary structure information could be inherently learned by the foundation models. Surprisingly,

the prediction performance in the cross-species setting showed a significant improvement when using the sequence embeddings from the RNA foundation models (**Fig. R2**). Given these results, we integrated the sequence embedding from the RNA foundation models RiNALMo in the revised version of MuSIC.

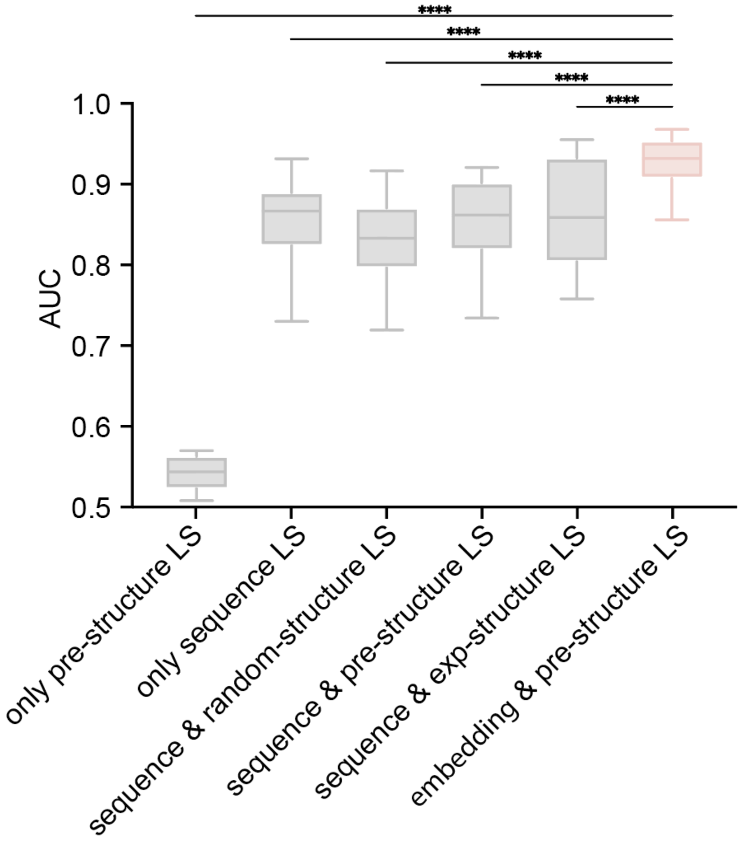

Figure R2 Contribution of RNA structural features to cross-species prediction.

Boxplot showing the performance of MuSIC using different input features with 200nt in cross-species tasks. **** *P* < 0.001, Wilcoxon test.

Excerpt (main text)

We replaced **Fig. R2** with **Supplementary Fig. 3e**. And we added relevant descriptions to the **Results** (L252 – L269), the **Discussion** (L544 – L549) and the **Methods** (L893 – L897) sections:

Results (L252 – L269):

Notably, incorporating predicted RNA structures could further enhance the AUC score to 0.85 in cross-species prediction (**Fig. 2f**). We confirmed that using predicted RNA structures could have better prediction performance than using the randomized RNA structures (**Supplementary Fig. 3e**). Furthermore, we assessed the prediction performance using experimentally measured *in vivo* RNA structures instead of the predicted RNA structures (**Supplementary Fig. 3e**). We used the predicted RNA structures in MuSIC because of the limited performance gains by using the *in vivo* RNA structures, as well as very few *in vivo* RNA structure datasets are available in other species.

The protein and RNA foundation models trained on large-scale unlabeled datasets provide potential binding features for RBP and RNA [45–48]. We incorporated protein foundation model (ProtT5 [48]) together with RNA foundation model (RiNALMo [46]) to extract high-dimensional representations for both RBPs and RNAs. Incorporating the ProtT5-derived RBP embeddings could improve the average AUC from 0.85 to 0.86. Furthermore, incorporating RiNALMo-derived RNA embeddings could lead to an additional improvement of average AUC from 0.86 to 0.91 (**Fig. 2f**; see **Methods** for details).

Discussion (L544 – L549):

Recent advances in protein and RNA foundation models have enabled the transfer of rich contextual and evolutionary information, improving RBP binding predictions for diverse RBPs from different cell lines and species [31,32,46–48,77]. Consistent with this trend, we showed that integrating protein and RNA foundation models substantially improves cross-species RBP–RNA interaction prediction, highlighting the complementary contributions of foundation models.

Methods (L893 – L897):

(ii) sequence and structural features: we constructed the models using different types of input data, including only sequences, only predicted structures, combined sequences and predicted structures, combined sequences and randomized structures, combined sequences and experimentally-measured structures, and combined embeddings and predicted structures.

Reviewer #1 comment 3

In Supplementary Fig. 3d, the t-SNE plots suggest improved separation between positive and negative samples after adding SE and residual blocks. However, the SE model vs Input, and the Residual vs FCL does not clearly show the difference. Have you considered quantifying this improvement using metrics? It would be helpful to understand how much the feature space becomes more discriminative beyond visual inspection.

RESPONSE:

Thanks for your constructive suggestion. We agree that visualization using t-SNE plots is insufficient to assess the feature-space discriminability of the input RNA sequences, particularly when the differences from different prediction blocks are subtle. In response, we quantified the differences between the positive and negative RNA sequences in the embedding space using Fréchet Inception Distance (FID), which measures the similarity of embeddings, where a higher value signifies better discriminability. As expected, we observed consistently FID increase after incorporating the SE block, the 1D residual block, and the RBP–RNA representation block (**Fig. R3**). Together with the t-SNE plots, these results indicate improved separation between the positive and negative RNA sequences in the learned feature space, and the architectural components of MuSIC can effectively enhance feature discriminability.

In addition, we compared the ability to separate the positive and negative RNA sequences in the feature space of MuSIC and other five computational methods using the same quantitative evaluation, FID. As expected, we found that MuSIC showed better performance than other computational methods (**Fig. R4**).

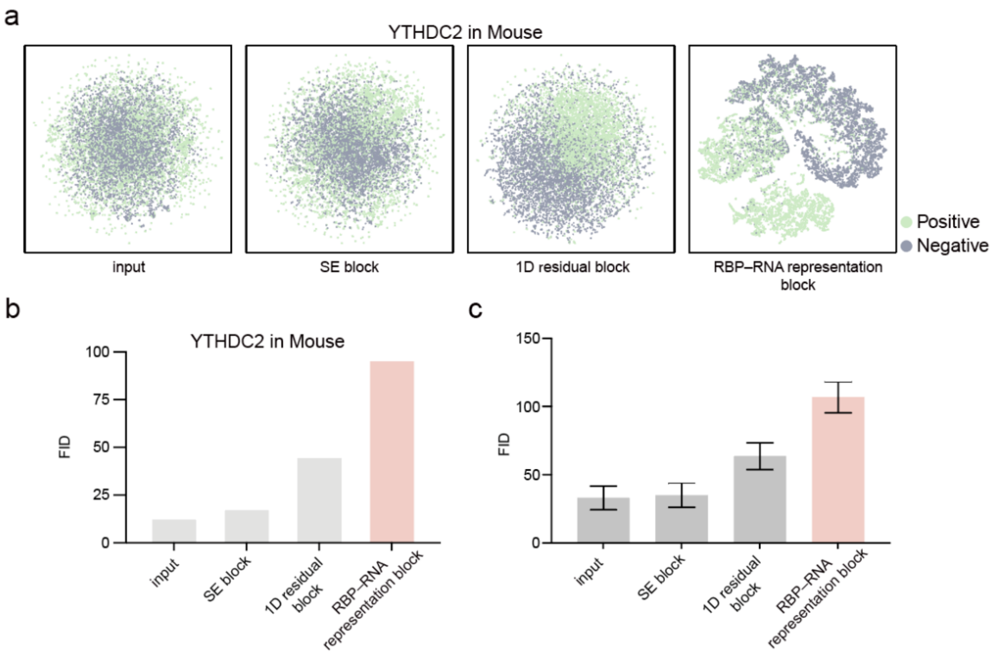

Figure R3 Quantitative assessment of feature-space separability using FID

(a) t-SNE clustering showing the output feature maps by different blocks (input, SE block, 1D residual block, RBP–RNA representation block) for separating the positive (cyan) and negative (grey) peaks. (b-c) Bar plots showing the FID which assesses the distance between the positive and negative feature distributions learned by different blocks for YTHDC2 (b) and all datasets (c).

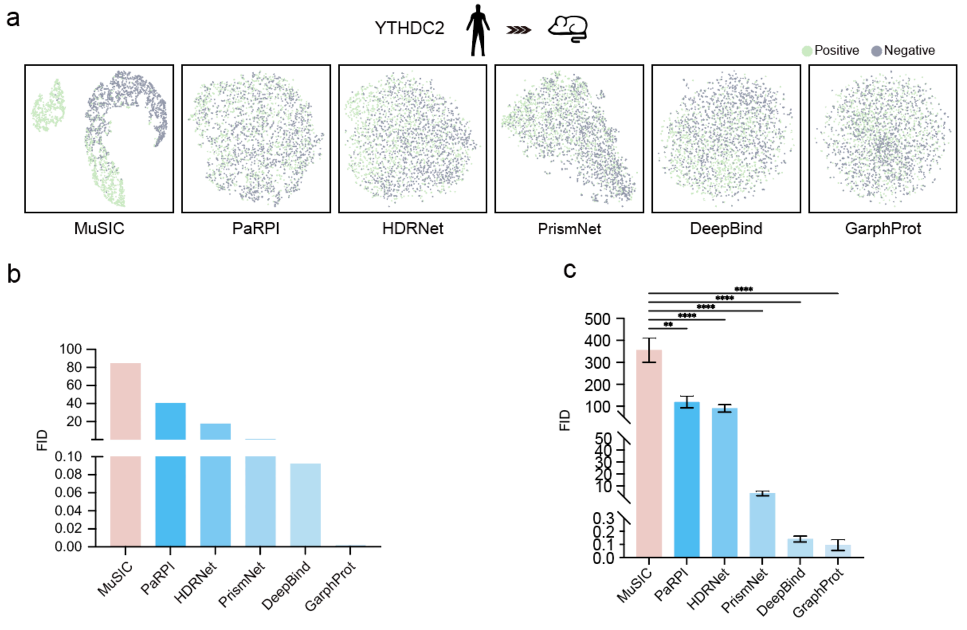

Figure R4 MuSIC outperforms other methods in output feature separability

(a) t-SNE clustering showing the output feature maps by MuSIC and other computational methods for separating the positive (cyan) and negative (grey) peaks. (b-c) Bar plots showing the FID of MuSIC (red) and other computational methods (blue) for (b) YTHDC2 and (c) all datasets. ** $P < 0.01$, **** $P < 0.001$, Wilcoxon test.

Excerpt (main text)

We replaced **Fig. R3a** with **Supplementary Fig. 3f**, **Fig. R3b** with **Supplementary Fig. 3g**, **Fig. R3c** with **Supplementary Fig. 3h**, **Fig. R4a** with **Fig. 3c**, **Fig. R4b** with **Fig. 3c**,and **Fig. R4c** with **Supplementary Fig. 5c**. And we added relevant descriptions to the **Results** (L273 – L281 and L330 – L332) and **Methods** (L913 – L916) sections:

Results (L273 – L281):

> We then characterized the contribution of each component of the MuSIC framework to the performance of feature learning using t-SNE and the Fréchet Inception Distance (FID), which measures the similarity of embeddings, with a higher value indicating better discriminability. The positive and negative samples of input RNA sequences cannot be clearly separated in the original feature space. After adding the SE and 1D residual block of the learning RNA module processing, the t-SNE clustering performance on the positive and negative samples could be significantly improved, thus leading to the apparent discriminative power in the RBP–RNA representation block of the learning interaction module (**Supplementary Fig. 3f-h**).

Results (L330 – L332):

> Consistently, MuSIC showed better performance in separating the positive and negative samples of input RNA sequences in the feature space than other computational methods (**Fig. 3c** and **Supplementary Fig. 5b,c**).

Methods (L913 – L916):

We extracted the features from the fully connected layers of MuSIC and the other five computational methods, including PaRPI [32], HDRNet [31], PrismNet [30], DeepBind [28], and GraphProt [26]. These features were projected into a two-dimensional space using t-SNE and FID to visualize and compare their classification performance.

Reviewer #1 comment 4

Although MuSIC demonstrates superior performance over existing methods, the evaluation is limited to a relatively small number of RBPs (14 for cross-species tasks). This narrow scope may limit the generalizability of the findings. Expanding the benchmark to include more diverse RBPs and species would enhance the credibility of the performance claims.

RESPONSE:

Thanks for your constructive suggestion. In response, we expanded the benchmark to include a more diverse set of RBPs and species. Specifically, we collected three RBPs datasets (Rps3, Fmr1 and Upf1) in *Drosophila melanogaster* (fly) from the modENCODE project to evaluate the prediction performance of MuSIC in the distantly related species.

We evaluated the prediction accuracy of the revised version of MuSIC and other computational methods in the cross-species prediction, where we trained the model in human and validated in mouse, zebrafish and fly (22 datasets). As expected, MuSIC outperformed other methods, showing an obvious improvement of average AUC score (MuSIC = 0.91, PaRPI = 0.58, HDRNet = 0.72, PrismNet = 0.70, DeepBind = 0.68, GraphProt = 0.71; **Fig. R5a**). For example, MuSIC showed higher AUC of 0.89 (from human to fly) and 0.82 (from fly to human) for Rps3 in the distantly related cross-species prediction (**Fig. R5b**). Consistent with these observations, MuSIC showed better performance in separating the positive and negative input RNA sequences in the feature space than other computational methods for Upf1 (**Fig. R5c**).

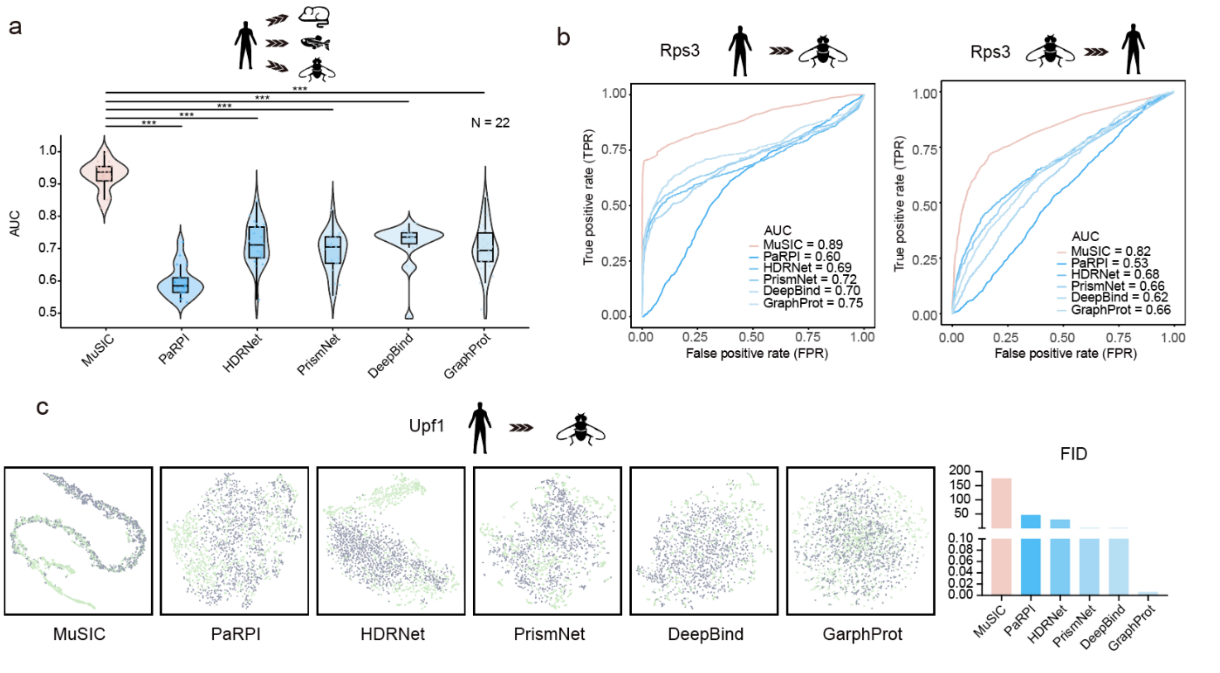

Figure R5 MuSIC outperforms other methods in expanded benchmark

(a) Violin plot showing the prediction accuracy of MuSIC (red) and other computational methods (blue) for 22 cross-species datasets. *** $P < 0.005$, Wilcoxon test. (b) ROC curves showing the prediction accuracy of MuSIC (red) and other computational methods (blue) for Rps3 (left: human to fly, right: fly to human). (c) Left, t-SNE clustering showing the output feature maps by MuSIC and other computational methods for separating the positive (cyan) and negative (grey) peaks. Right, bar plot showing the FID of MuSIC (red) and other computational methods (blue).

Excerpt (main text)

We replaced **Fig. R5a** with **Fig. 3a**, **Fig. R5b** with **Supplementary Fig. 5a** and **Fig. R5c** with **Supplementary Fig. 5b**. And we added relevant descriptions to the **Results** (L196 – L202 and L316 – L328) and the **Methods** (L637 – L640) sections:

Results (L196 – L202):

> We initially found 23 homologous RBPs between human and mouse, one homologous RBP between human and zebrafish from POSTAR3 [33], and three homologous RBPs between human and fly with available datasets from the modENCODE project [55] (**Supplementary Fig. 2f,g**). In total, 44 datasets from 17

RBPs were curated for the training and validation of the cross-species model (see **Methods** for details; **Supplementary Table 3**).

Results (L316 – L328):

We then evaluated the prediction accuracy of MuSIC and other computational methods in the cross-species prediction across 44 datasets from human, mouse, zebrafish, and fly. MuSIC outperformed other methods, showing an obvious improvement of in AUC (average AUC: MuSIC = 0.91, PaRPI = 0.58, HDRNet = 0.72, PrismNet = 0.70, DeepBind = 0.68, GraphProt = 0.71; **Fig. 3a**), as well as in the accuracy (ACC) and Area Under the Precision-Recall Curve (PRAUC) metrics (**Supplementary Fig. 4b-d**). In addition, we found that MuSIC achieves high accuracy for predicting RBP–RNA interactions in both the closely related predictions (e.g., human to mouse) and distantly related predictions (e.g., human to zebrafish or fly) (**Supplementary Fig. 4e,f**). For example, MuSIC showed AUCs of 0.95 for YTHDC2 (mouse) and 0.91 for Fmr1 (fly) in the cross-species prediction, a significant improvement than other computational methods (**Fig. 3b** and **Supplementary Fig. 5a**).

Methods (L637 – L640):

For the within-species prediction, 230 datasets of 186 RBPs from human, mouse, zebrafish and fly were used. For the cross-species prediction, 34 datasets of 13 RBPs shared between human and mouse, 4 datasets of one RBP shared between human and zebrafish and 6 datasets of 3 RBP shared between human and fly were used.

Reviewer #1 comment 5

While motif rediscovery in non-human species is promising, the validation of predicted motifs relies heavily on known motifs from databases. This may bias the evaluation toward well-characterized RBPs. Incorporating de novo motif discovery validation or experimental confirmation for novel motifs would provide stronger evidence of MuSIC's generalizability.

RESPONSE:

Thanks for this important suggestion. In response, we performed *de novo* motif discovery based on the predictions by MuSIC to identify the motifs that have not been well annotated in the existing motif databases. Here we exemplify the *de novo* motif discovery with TRA2A and HNRNPK. For TRA2A, we identified enriched poly-GAA binding motifs in mouse and zebrafish. For HNRNPK, we revealed a conserved binding preference of polyC motifs in mouse and zebrafish (**Fig. R6**). We then synthesized corresponding RNA oligonucleotides and performed RNA pull-down assays to experimentally validate these *de novo* motifs. Consistent with the computational predictions, the experimental results provided direct evidence for the *de novo* motifs of TRA2A and HNRNPK (**Fig. R6**). These results revealed and confirmed the generalizability of MuSIC in identifying enriched binding preference for the RBPs in non-human species.

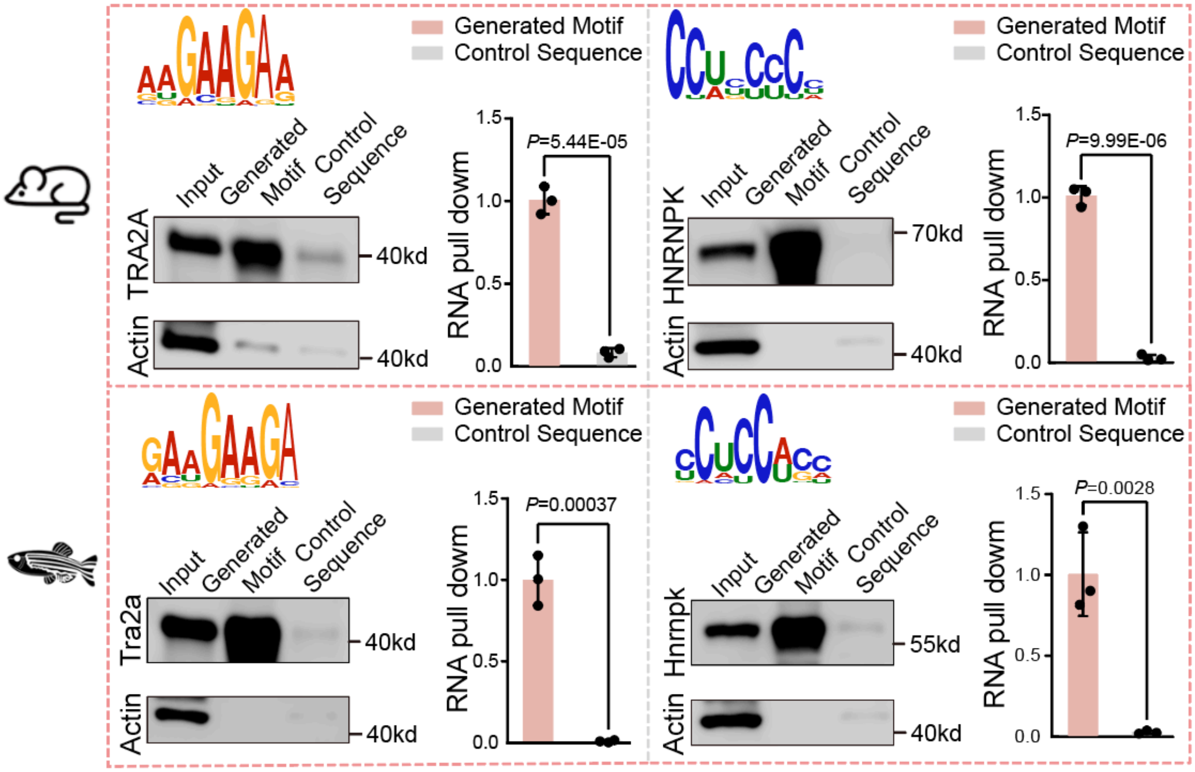

Figure R6 Experimental validation supports the generalizability of MuSIC

Examples showing *in vitro* RNA pull-down assay (n = 3) used to validate the predicted motifs for TRA2A and HNRNPK in mouse and zebrafish. For each panel, left shows the predicted motif (top) and the corresponding immunoblots (bottom). On the right, bar plot quantifying the enrichment of protein binding to generated motif and the control sequence. Red bar represents generated motif, and grey bar represents control sequence. All *P* values are calculated using two-tailed unpaired Student's t-test.

Excerpt (main text)

We replaced **Fig. R6** with **Fig. 4c**. And we added relevant descriptions to the **Results** (L392 – L398) section:

Results (L392 – L398):

> Furthermore, we found that the inferred RBP-binding motifs with high confidence showed distinct degree of conservation during evolution (**Supplementary Fig. 7d** and **Supplementary Table 5**). For example, poly-GAA motifs for TRA2A and polyC motifs for HNRNPK were predicted in diverse species ranging from human to yeast. We then

performed the RNA pull-down assays to experimentally validate these de novo motifs in mouse and zebrafish (**Fig. 4c**). These results confirmed the generalizability of MuSIC in identifying enriched binding preference for the RBPs in non-human species.

Reviewer #1 comment 6

While MuSIC demonstrates strong performance for RBPs with well-characterized binding profiles and conservation data, it remains unclear how the model handles RBPs with limited or ambiguous training data—particularly those lacking annotated RBDs or CLIP-seq datasets. This is especially relevant for de novo RBD discovery, where no prior binding motifs or structural templates exist.

RESPONSE:

Thanks for raising this important question. We agree that it is crucial to make MuSIC appliable to the RBPs with limited CLIP-seq data or ambiguous RBD annotations. To this end, we first trained the MuSIC model using a label smoothing strategy guided by the evolutionary conservation of RBPs, thereby enabling cross-species modeling in a higher-dimensional feature space. Next, we incorporated protein foundation model (ProtT5) together with RNA foundation model (RiNALMo) to extract high-dimensional representations for both RBPs and RNAs in the revised version of MuSIC. ProtT5 has demonstrated strong transfer-learning performance across the tasks related to protein binding, evolutionary conservation, and superfamily classification by learning relationships between protein sequence and structure. We assume that the ProtT5 embeddings can effectively capture the core functional domains within RBPs, i.e., RBDs. The RNA sequence embeddings were further combined with the predicted RNA secondary structures, and passed through the learning RNA module to obtain the RNA representations. In the learning interaction module, the model learned the RBP–RNA interaction representations by integrating the RNA representations (the query matrix Q) and RBP representations (the key matrix K and value matrix V). Subsequently, these representations are fed into a multilayer perceptron (MLP) classifier to generate RBP–RNA binding affinity predictions.

We systematically evaluated how the training strategy and dimensional representations could influence the prediction performance of cross-species prediction tasks (**Fig. R7**). We found that the label smoothing strategy could improve the average AUC score from 0.75 to 0.84 in the cross-species prediction tasks (**Fig. R7**). Incorporating

the predicted RNA structure embeddings could improve the average AUC from 0.84 to 0.85. Incorporating the ProtT5-derived RBP embeddings could improve the average AUC from 0.85 to 0.86. Furthermore, incorporating RiNALMo-derived RNA embeddings could lead to an additional improvement of average AUC from 0.86 to 0.91. These results revealed the advantage and power of incorporating the protein and RNA foundation models in our revised version of MuSIC, which can handle the cases when the RBPs lack high-quality RBD annotations and CLIP-seq datasets.

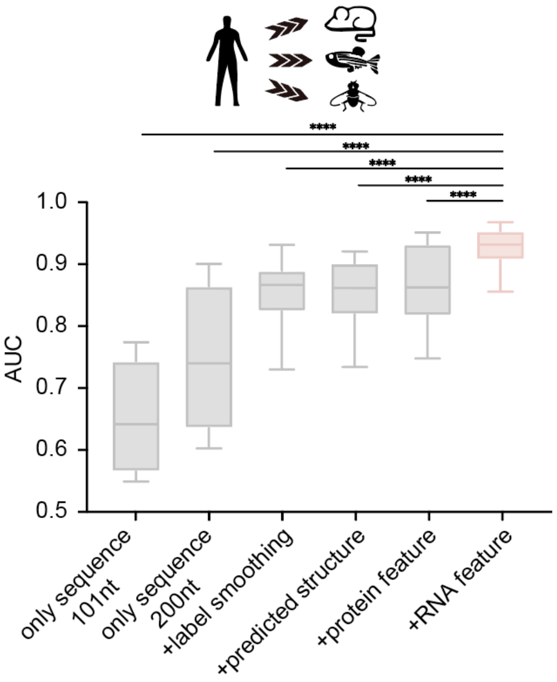

Figure R7 Contribution of training strategy and dimensional representations to cross-species prediction

Bar plot showing the performance of MuSIC using different features in cross-species tasks. **** *P* < 0.001, Wilcoxon test.

Excerpt (main text)

We replaced **Fig. R7** with **Fig. 2f**. And we added relevant descriptions to the **Results** (L251 – L254 and L262 – L271) and the **Discussion** (L538 – L552):

Results (L251 – L254):

The inclusion of label smoothing, and gradient adaptation could further improve the AUC score from 0.75 to 0.84 (**Fig. 2f**). Notably, incorporating predicted RNA structures could further enhance the AUC score to 0.85 in cross-species prediction (**Fig. 2f**).

Results (L262 – L271):

The protein and RNA foundation models trained on large-scale unlabeled datasets provide potential binding features for RBP and RNA [45–48]. We incorporated protein foundation model (ProtT5 [48]) together with RNA foundation model (RiNALMo [46]) to extract high-dimensional representations for both RBPs and RNAs. Incorporating the ProtT5-derived RBP embeddings could improve the average AUC from 0.85 to 0.86. Furthermore, incorporating RiNALMo−derived RNA embeddings could lead to an additional improvement of average AUC from 0.86 to 0.91 (**Fig. 2f**; see **Methods** for details). Taken together, these results reveal that extending the sequence length, applying label smoothing and incorporating foundation models could significantly improve the performance of cross-species generalization.

Discussion (L538 – L552):

Inspired by the concepts from computer vision, we implemented label smoothing in the model to further improve prediction performance [36–38]. We showed that label smoothing and the gradient weight adaptation mechanism could significantly improve cross-species predictions. This strategy reflects the inherently dynamic nature of biomolecular recognition, which operates on a continuum rather than a binary decision framework [76]. Recent advances in protein and RNA foundation models have enabled the transfer of rich contextual and evolutionary information, improving RBP binding predictions for diverse RBPs from different cell lines and species [31,32,46–48,77]. Consistent with this trend, we showed that integrating protein and RNA foundation models substantially improves cross-species RBP–RNA interaction prediction, highlighting the complementary contributions of foundation models. Importantly, the

integration of conservation-aware label smoothing and foundation model representations for RBP and RNA enables MuSIC to more accurately identify dynamic RBP–RNA interactions across species.

Reviewer #1 comment 7

The SNV impact analysis is a valuable application, yet the validation appears limited to a few illustrative examples. A more systematic evaluation—such as comparing MuSIC predictions with high-throughput mutagenesis or disease variant databases—would better demonstrate the model's utility in variant interpretation.

RESPONSE:

Thanks for your valuable suggestion. In response, we performed a large-scale analysis using genetic variants from the ClinVar database. Specifically, we extracted the variants annotated as single nucleotide variants (SNVs), and categorized them into pathogenic and benign ones based on their annotations in the ClinVar database. We then predicted the effects on RBP–RNA binding affinity for the SNVs from each category using MuSIC (**Fig. R8a**). Interestingly, we found that the pathogenic SNVs were associated with larger effects on RBP binding compared with benign ones (**Fig. R8b**). For example, the c.-103C>T mutation in the 5′ UTR of GJB1 has been revealed to disrupt an internal ribosomal entry site (IRES) and impair the translational efficiency of GJB1, which is associated with the development of X-linked Charcot–Marie–Tooth neuropathy (PMID: 10931843, 20301548). These results suggest that pathogenic SNVs could be implicated in the pathogenesis to diverse human diseases by altering RBP binding and thereby perturbing downstream post-transcriptional regulatory processes.

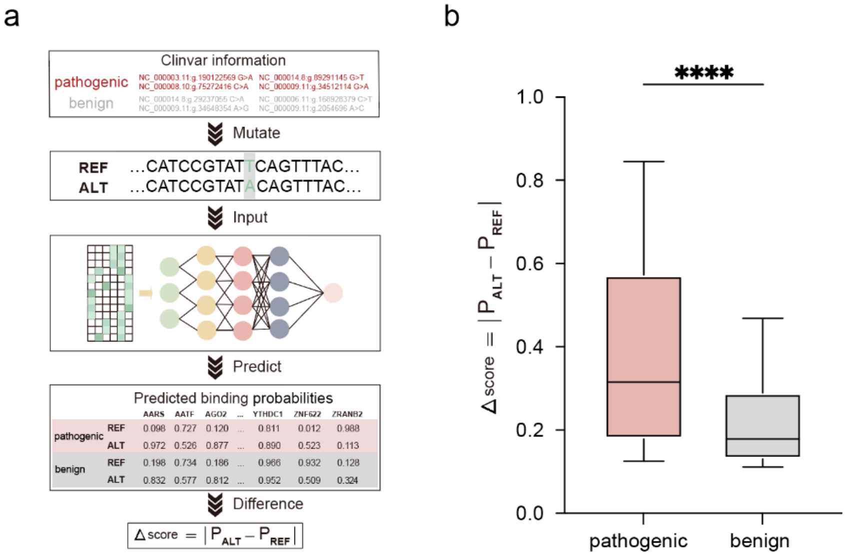

Figure R8 Differential effects of pathogenic and benign SNVs on RBP–RNA interactions

(a) Schematic showing the workflow for quantifying the effects of pathogenic and benign SNVs on RBP binding. (b) Bar plot showing the effects of pathogenic (red) and benign (grey) SNVs on RBP–RNA binding affinity. **** $P < 0.001$, Mann–Whitney U test.

Excerpt (main text)

We replaced **Fig. R8a** with **Supplementary Fig. 10c** and **Fig. R8b** with **Supplementary Fig. 10d**. And we added relevant descriptions to the **Results** (L457 – L467) and the **Methods** (L1030 – L1043) sections:

Results (L457 – L467):

> Furthermore, we performed systematic analyses on the SNVs from ClinVar database [7] using MuSIC to quantify their disruption effects on RBP–RNA binding affinity (**Supplementary Fig. 10c**; see **Methods** for details). Interestingly, we found that the pathogenic SNVs exhibited significantly larger disruption effects on RBP binding compared with the benign ones (**Supplementary Fig. 10d**). For example, the c.-103C>T mutation in the 5′ UTR of GJB1 has been revealed to disrupt an internal ribosomal entry site (IRES) and impair GJB1 translation, which is associated with the development of X-linked Charcot–Marie–Tooth neuropathy [62,63]. These results indicate that the pathogenic SNVs could be implicated in the pathogenesis of diverse disease by altering RBP binding and thereby perturbing downstream post-transcriptional regulatory processes.

Methods (L1030 – L1043):

> We downloaded SNVs curated in the ClinVar [7]. Variants were categorized into pathogenic (Pathogenic; Likely_pathogenic; Pathogenic/Likely_pathogenic) and benign (Benign; Likely_benign; Benign/Likely_benign) SNVs according to their annotated clinical significance. For each SNV, human RNA sequences were extracted using GAP (https://github.com/lipan6461188/GAP), and paired RNA sequences containing the reference (REF) and alternative (ALT) alleles were generated.

We then predicted RBP–RNA binding probabilities on both REF- and ALT-containing sequences for 184 RBPs using MuSIC. The effect of the RBP-binding affinity was quantified as the absolute difference between the predicted binding probabilities of the ALT and REF alleles:

$$\Delta score = |P_{ALT} - P_{REF}|$$

where P_{REF} and P_{ALT} denote the predicted binding probabilities for the ALT and REF alleles, respectively.

Reviewer #1 comment 8

The figure in Supp Table 2 about the definition of different conserved level is confusing.

RESPONSE:

Thanks for pointing out this issue. In this study, the conservation level of RBPs was defined based on multiple conservation metrics derived from **Supplementary Table 1**, including full-length sequence similarity, RBD sequence similarity, full-length structural similarity, and RBD structural similarity across species. Thresholds for sequence similarity (70%) and structural similarity (50%) were applied to each of these metrics, and RBPs were subsequently categorized into different conservation levels (highly conserved, considerable conserved, intermediately conserved and weakly conserved) by jointly considering these criteria rather than relying on a single metric.

We have revised the descriptions on the conservation metrics in **Supplementary Table 2**, as well as how the conservation levels of these RBPs were defined in the revised manuscript.

Excerpt (main text)

We added relevant descriptions regarding the definition of the conservation metrics in **Supplementary Table 2**:

Name	Definition
SeqS: full-length sequence similarity	full-length sequence similarity
StrS: full-length structure similarity	(full-length structure TM-score * 100 + full-length structure pLDDT) / 2

DSeqS: domain sequence similarity	RBD sequence similarity
DStrS: domain structure similarity	(RBD structure TM-score*100 + RBD structure pLDDT) / 2
Highly conservation RBP	RBPs with conserved RBD sequence and structure (DSeqS ≥ 70% and DStrS ≥ 0.5), together with high conservation at the full-length level (SeqS ≥ 70% or StrS ≥ 0.5).
Middle-Highly conservationID	RBPs showing strong conservation in either the RBD or full-length metrics, but not consistently across all dimensions.
Middle conservation	RBPs with moderate conservation, meeting the threshold in only one dimension (sequence or structure) at either the full-length or RBD level.
Lowly conservation	RBPs failing to meet conservation thresholds at both the full-length and RBD levels, indicating substantial evolutionary divergence.

Point-to-Point response to Reviewer #2

Reviewer #2: I co-reviewed this manuscript with one of the reviewers who provided the listed reports. This is part of the Nature Communications initiative to facilitate training in peer review and to provide appropriate recognition for Early Career Researchers who co-review manuscripts.

RESPONSE:

Thanks for your helpful revision.

Point-to-Point response to Reviewer #3

Reviewer #3: This paper introduces MuSIC (Multi-Species RBP–RNA Interactions using Conservation), a deep learning framework for predicting RNA-binding protein (RBP)–RNA interactions across multiple species. The key contributions are as follows:

1. Cross-species prediction: MuSIC predicts RBP–RNA interactions across 11 diverse species, from human to yeast.
2. Conservation-Guided Label Smoothing: The model integrates multi-level RBP conservation patterns (sequence and structure) into a conservation-weighted label smoothing scheme and employs gradient weight adaptation to dynamically balance loss components during training.
3. Variant effect prediction: MuSIC quantifies the impact of homologous single-nucleotide variants (SNVs) on RBP binding across species, with experimental validation in mouse models.

RESPONSE:

Thanks for your kind summary and comments on MuSIC's key contributions. As you mentioned, MuSIC was developed to address the long-standing challenge of cross-species RBP–RNA interaction prediction, and demonstrates robust applicability across 11 evolutionarily diverse species. The computational framework integrates conservation-guided label smoothing with gradient-based weight adaptation to effectively balance training objectives, and further enables systematic SNV effect prediction through the analysis of homologous SNVs with experimental validations in mouse. We hope that MuSIC will be a power tool for studying the post-transcriptional regulatory mechanisms across different species.

We also appreciate your constructive suggestions, and have substantially revised the computational framework of MuSIC by incorporating large-scale pre-trained protein and RNA foundation models, as well as integrating foundation model representations with multi-scale convolutional and transformer-based contextual modeling. This enhanced architecture could augment the AUC scores in the within-species tasks (from 0.88 to 0.95),

as well as the cross-species tasks (from 0.74 to 0.88), particularly in the evolutionarily distant species (from 0.58 to 0.81). The revised version of MuSIC outperforms existing deep learning- and transfer learning-based methods in both within-species and cross-species tasks.

Reviewer #3 comment 1

While the work is well executed and biologically grounded, it appears to be incremental rather than groundbreaking. Several models already address cross-species RBP prediction—such as PaRPI (which integrates cross-protocol and cross-batch datasets) and RBPsuite 2.0 (which achieves wide species and protein coverage using deep learning)—yet these are not included in the comparison, weakening the claim of state-of-the-art performance.

RESPONSE:

Thanks for pointing out this issue that several existing models, such as PaRPI and RBPsuite 2.0, can make the cross-species prediction of RBP–RNA interactions. We carefully checked the usage of PaRPI, RBPsuite 2.0 and other related tools, and confirmed that these tools were not developed for the application of the cross-species prediction tasks.

PaRPI was developed using training data from 6 cell lines in human. The authors trained the prediction model in each cell type separately. Thus, PaRPI was not desighed for the cross-species prediction task. The cross-species prediction accuracy (ACC) by PaRPI for U2AF2 and SRRM4 in mouse was 0.7435 and 0.6036, respectively (Figures 5c and 5d from the original PaRPI paper, PMID 41028220). Notably, the prediction accuracy (ACC) for Fmr1 in *Drosophila melanogaster* (fly) was 0.3642, significantly lower than expected. These results suggest that PaRPI is not effective for the cross-species prediction task. In our revised version of MuSIC, the prediction accuracy can be improved to 0.8499 (U2AF2 in mouse), 0.7516 (SRRM4 in mouse) and 0.526 (Fmr1 in fly), respectively.

RBPsuite 2.0 (http://www.csbio.sjtu.edu.cn/bioinf/RBPsuite/index.html) was developed for predicting RBP binding sites using RNA sequences based on iDeepS. Similar to other methods like PrismNet and HDRNet, iDeepS was trained using CLIP-seq datasets from particular species, and was not designed for the cross-species prediction tasks. In addition, we encountered webserver failure of RBPsuite 2.0 and configuration

issues of iDeepS when we tried to systematically evaluate the prediction performance of within-species and cross-species tasks using 2500 sequences for each complete validation dataset. Therefore, we down sampled the 2500 sequences to generate a random subset of 100 sequences with a 1:1 positive-to-negative ratio for the cross-species tasks. As noted in the original iDeepS paper (PMID 29970003), the authors reported prediction accuracy for 17 within-species datasets (see Additional file 3 in the original iDeepS paper). To make a fair comparison, we collected the 17 within-species datasets from iDeepS, and compared the prediction performance on the 38 cross-species tasks using MuSIC and RBPsuite 2.0. We found that our revised version of MuSIC consistently outperformed RBPsuite 2.0 in both within-species (**Fig. R9a**) and cross-species (**Fig. R9b**) tasks. Given the running time limitation, we found it challenging to assess the performance of RBPsuite 2.0 using the complete validation datasets. Therefore, the RBPsuite 2.0 was not included as a baseline in the revised manuscript.

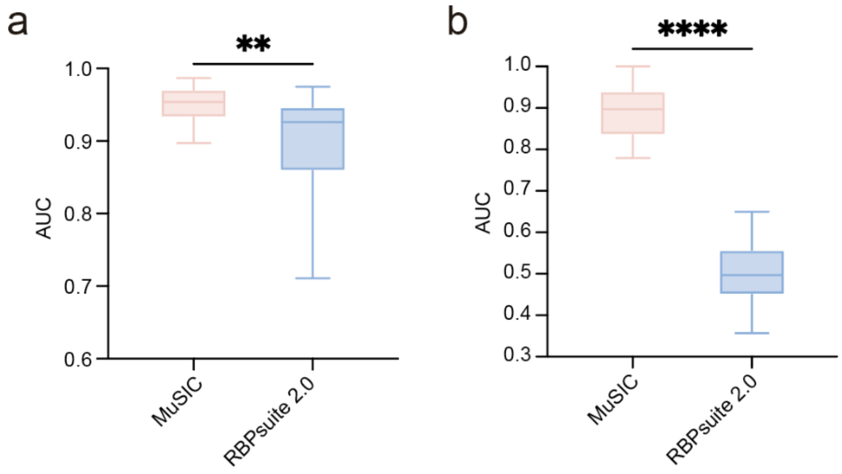

Figure R9 MuSIC outperforms RBPsuite 2.0 in within- and cross-species prediction

Boxplots showing the prediction accuracy of MuSIC (red) and RBPsuite 2.0 (blue) for 17 within-species datasets (a) and 38 cross-species datasets (b). ** $P < 0.01$, **** $P < 0.001$, Wilcoxon test.

We then systematically compared the prediction performance on 274 datasets from 186 RBPs using our revised version of MuSIC and existing computational methods, which included both within-species (230 datasets from 186 RBPs) and cross-species (44 datasets from 17 RBPs) tasks. We included five existing computational methods,

including PaRPI, HDRNet, PrismNet, DeepBind, and GraphProt. As expected, MuSIC showed better prediction accuracy than all the other methods for the within-species tasks (average AUC: MuSIC = 0.95, PaRPI = 0.91, HDRNet = 0.88, PrismNet = 0.86, DeepBind = 0.67, GraphProt = 0.72; **Fig. R10**).

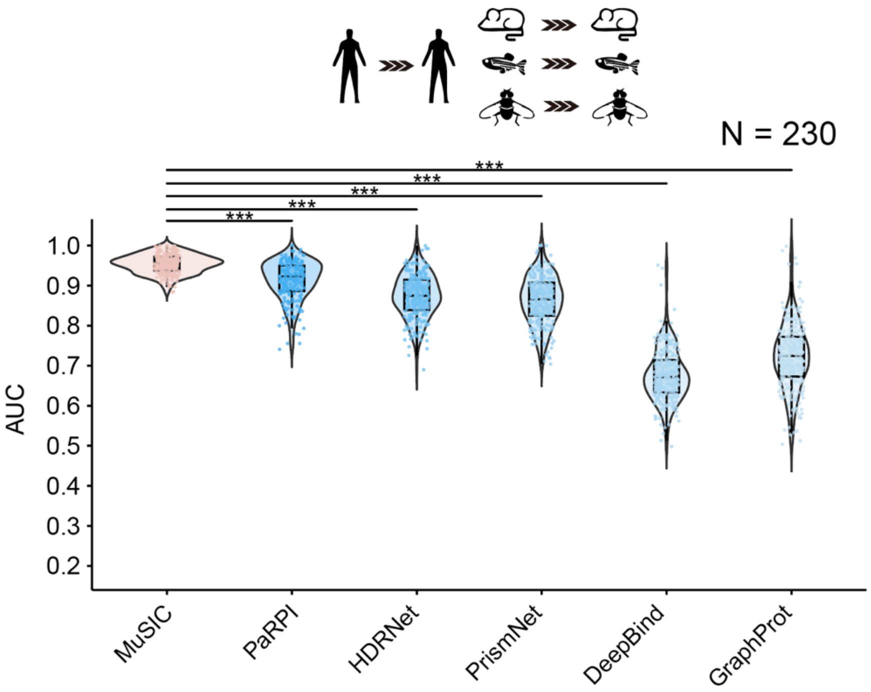

Figure R10 MuSIC outperforms other methods in within-species prediction

Violin plot showing the prediction accuracy of MuSIC (red) and other computational methods (blue) for 230 within-species datasets. *** $P < 0.005$, Wilcoxon test.

We then performed similar analyses for the cross-species tasks across 44 datasets from human, mouse, zebrafish, and fly, with the additional benchmark datasets from 3 RBPs in fly (Rps3, Fmr1, and Upf1). We found that our revised version of MuSIC showed significant improvement of AUC (average AUC: MuSIC = 0.91, PaRPI = 0.58, HDRNet = 0.72, PrismNet = 0.70, DeepBind = 0.68, GraphProt = 0.71; **Fig. R11a**), accuracy (ACC) and Area Under the Precision-Recall Curve (PRAUC) metrics (**Fig. R11b-d** and **Fig. R12**).

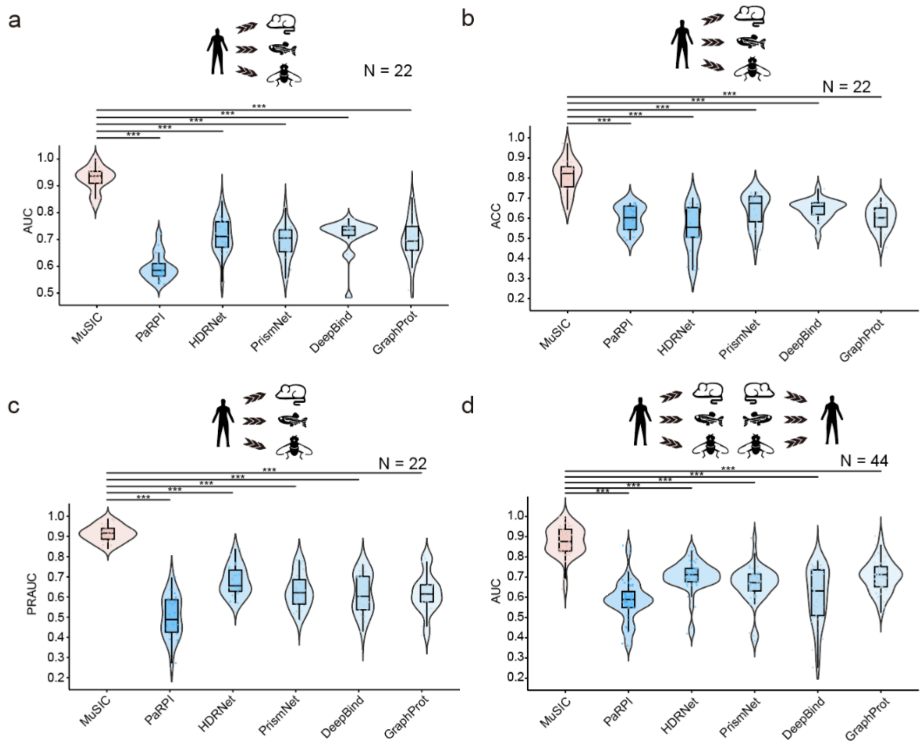

Figure R11 MuSIC outperforms other methods in cross-species prediction

(a-c) Violin plot showing the prediction accuracy (AUC, ACC and PRAUC) of MuSIC (red) and other computational methods (blue) for 22 cross-species datasets. *** $P < 0.005$, Wilcoxon test. (d) Violin plot showing the prediction accuracy of MuSIC (red) and other computational methods (blue) for 44 cross-species datasets.

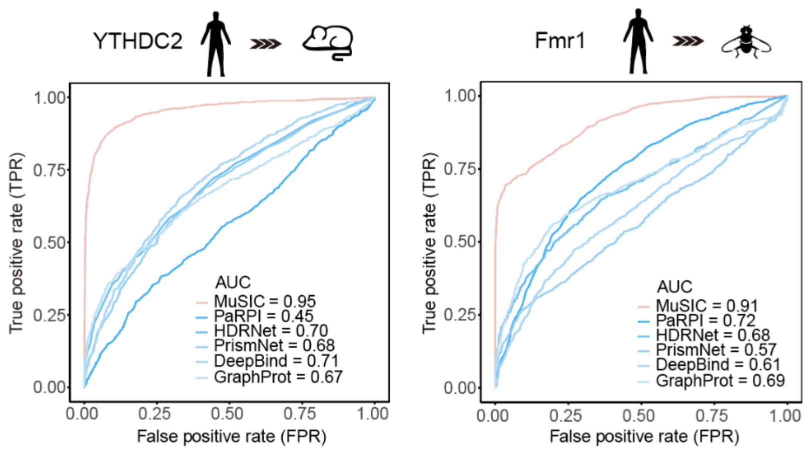

Figure R12 MuSIC outperforms other methods for YTHDC2 and Fmr1 in cross-species prediction

ROC curves showing the prediction accuracy of MuSIC (red) and other computational methods (blue) for two RBPs, YTHDC2 (left) and Fmr1 (right).

Excerpt (main text)

We replaced **Fig. R10** with **Supplementary Fig. 4a**, **Fig. R11a** with **Fig. 3a**, **Fig. R11b** with **Supplementary Fig. 4b**, **Fig. R11c** with **Supplementary Fig. 4c**, **Fig. R11d** with **Supplementary Fig. 4d**, and **Fig. R12** with **Fig. 3b**. And we added relevant descriptions to the **Results** (L308 – L330) and the **Methods** (L905 – L911) sections:

Results (L308 – L330):

> Briefly, we performed a systematic comparison of prediction accuracy with five existing state-of-the-art computational methods, including PaRPI [32], HDRNet [31], PrismNet [30], DeepBind [28], and GraphProt [26]. As expected, MuSIC showed better prediction accuracy than other methods for the within-species prediction (average AUC: MuSIC = 0.95, PaRPI = 0.91, HDRNet = 0.88, PrismNet = 0.86, DeepBind = 0.67, GraphProt = 0.72) (**Supplementary Fig. 4a**).
>
> We then evaluated the prediction accuracy of MuSIC and other computational methods in the cross-species prediction across 44 datasets from human, mouse, zebrafish, and fly. MuSIC outperformed other methods, showing an obvious improvement of in AUC (average AUC: MuSIC = 0.91, PaRPI = 0.58, HDRNet = 0.72, PrismNet = 0.70, DeepBind = 0.68, GraphProt = 0.71; **Fig. 3a**), as well as in the accuracy (ACC) and Area Under the Precision-Recall Curve (PRAUC) metrics (**Supplementary Fig. 4b-d**). In addition, we found that MuSIC achieves high accuracy for predicting RBP–RNA interactions in both the closely related predictions (e.g., human to mouse) and distantly related predictions (e.g., human to zebrafish or fly) (**Supplementary Fig. 4e,f**). For example, MuSIC showed AUCs of 0.95 for YTHDC2 (mouse) and 0.91 for Fmr1 (fly) in the cross-species prediction, a significant improvement than other computational methods (**Fig. 3b** and **Supplementary Fig. 5a**). Taken together, these results showed that MuSIC could improve the prediction accuracy of RBP–RNA interactions than other computational methods, especially under the cross-species scenario.

Methods (L905 – L911):

> We compared the prediction performance of MuSIC with other recently published computational methods, including PaRPI (https://github.com/ljquanlab/PaRPI) [32], HDRNet (https://github.com/zhuhr213/HDRNet) [31], PrismNet (https://github.com/kuixu/PrismNet) [30], DeepBind (https://github.com/jisraeli/DeepBind) [28], and GraphProt (https://github.com/dmaticzka/GraphProt) [26]. Notably, to make a fair comparison, each of these computational methods was trained and validated on the same datasets as used in MuSIC, using their default parameter settings.

Reviewer #3 comment 2

Moreover, MuSIC's cross-species performance gains are primarily limited to closely related species (e.g., human→mouse). Prediction accuracy drops markedly for distant taxa (e.g., fly, yeast), indicating restricted generalization beyond highly conserved domains.

RESPONSE:

Thanks for raising this critical concern. It is necessary to compare the prediction results by MuSIC with those by large-scale pre-trained RNA foundation models (refer to **Reviewer #3 comment 3**). The large-scale pre-trained RNA foundation models trained on extensive RNA sequence could provide feature representations capturing nucleotide context information and long-range dependencies. Here, we compared the prediction performance of our original version of MuSIC with two presentative RNA foundation models, i.e., RNA-FM and RiNALMo. RNA-FM was the first RNA foundation model, and RiNALMo was the one containing the most parameters. Surprisingly, we found that MuSIC outperformed both RNA-FM and RiNALMo in the closely related species (**Fig. R13a**). However, in the conditions of distantly related species, RNA foundation models alone can surpass the original version of MuSIC (**Fig. R13b**).

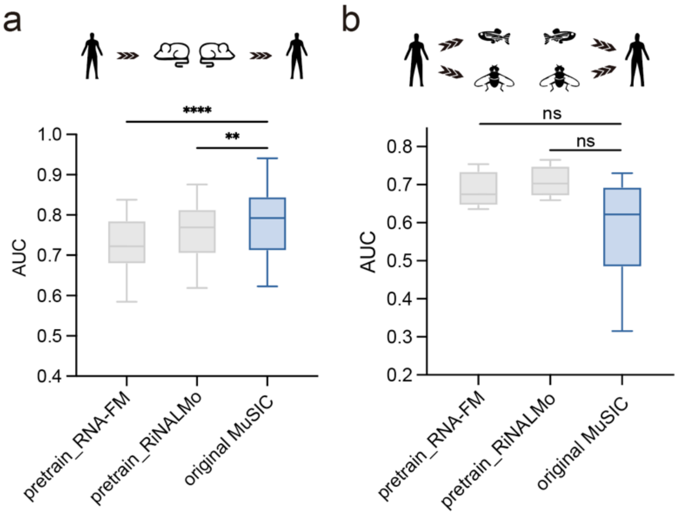

Figure R13 RNA foundation models outperform the original MuSIC in distantly related species prediction tasks

Boxplots showing the performance of the original version of MuSIC (blue) and other RNA foundation models (grey) across closely related species (a) and distantly related species (b). ns $P > 0.05$, ** $P < 0.01$, **** $P < 0.001$, Wilcoxon test.

Inspired by these results, we hypothesized that incorporating the existing pre-trained protein and RNA foundation models would improve the feature extraction for RBPs and RNAs, thus leading to better prediction performance, especially in the condition of distantly related species. Briefly, we first used the pre-trained protein foundation model ProtT5 for extracting RBP features, as well as the pre-trained RNA foundation model RiNALMo for extracting RNA features, respectively (**Fig. R14a**). Then, we combined the RNA sequence features with the predicted RNA secondary structure, and passed them through the learning RNA module to obtain the RNA representations (**Fig. R14b**). In the learning interaction module, the RNA representations (the query matrix Q) and the RBP features (the key matrix K and value matrix V) were integrated to learn the RBP–RNA representations, which were further input into a multilayer perceptron (MLP) classifier for predicting the binding affinity between specific RNA sequence and RBP (**Fig. R14c**).

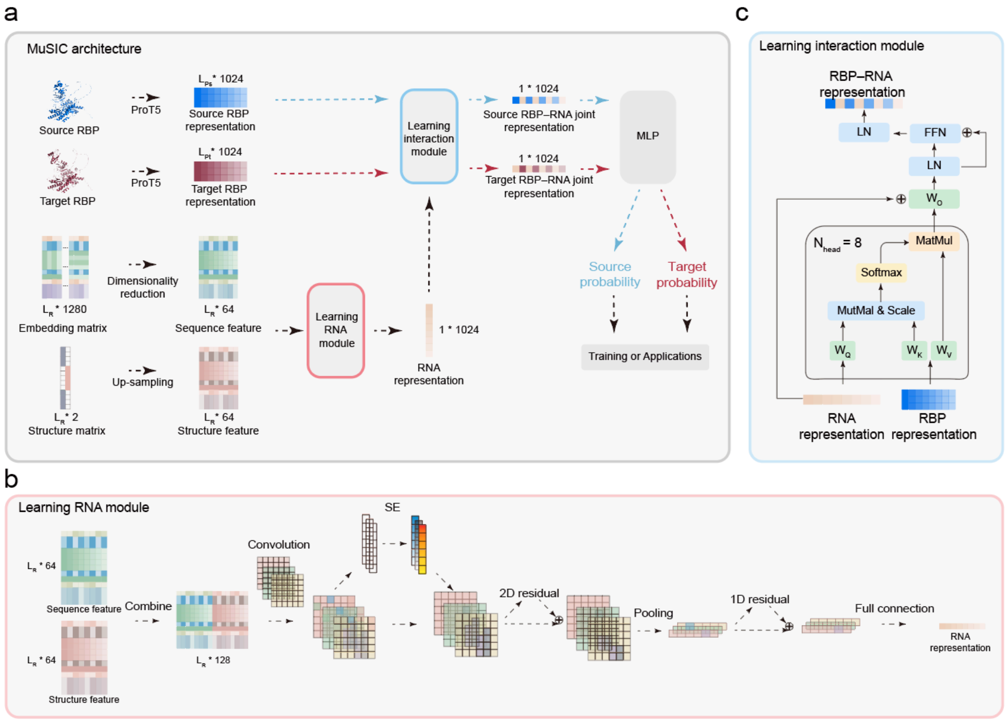

Figure R14 Overview of the revised MuSIC architecture

(a) Overview of the deep learning model architecture. RBP sequences are encoded using ProtT5, and RNA features are processed and combined to generate RNA representations. These are integrated with RBP representations to predict binding probabilities via the MLP. (b) Overview of the learning RNA module. RNA features are processed through convolutional layers, SE modules, residual blocks, pooling, and a fully connected layer to generate RNA representations. (c) Overview of the learning interaction module. The RNA and RBP representations are integrated using multi-head cross-attention and a feed-forward network to generate the joint representations.

We then evaluated the prediction performance of this revised version of MuSIC in the cross-species tasks. We found that it showed significant improvements compared to its original version. The original version of MuSIC achieves an average AUC score of 0.74 across the 44 datasets. In the revised version of MuSIC, incorporating the protein foundation model component can enhance the AUC score to 0.79, and further incorporating the RNA foundation model component can enhance the AUC score to 0.88 (**Fig. R15**).

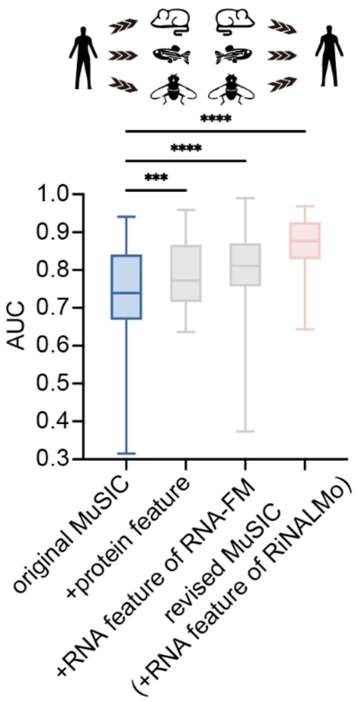

Figure R15 Foundation models enhance MuSIC's performance in cross-species tasks

Boxplot showing the performance of the original version of MuSIC (blue), the revised version of MuSIC (red), and other methods (grey) across 44 cross-species datasets. *** $P < 0.005$, **** $P < 0.001$, Wilcoxon test.

Notably, the revised version of MuSIC not only improves performance in closely related cross-species tasks from 0.79 to 0.89 (**Fig. R16a**), but also demonstrates substantial performance gains in the distantly related species (zebrafish and fly), with the average AUC rising from 0.58 to 0.81 (**Fig. R16b**).

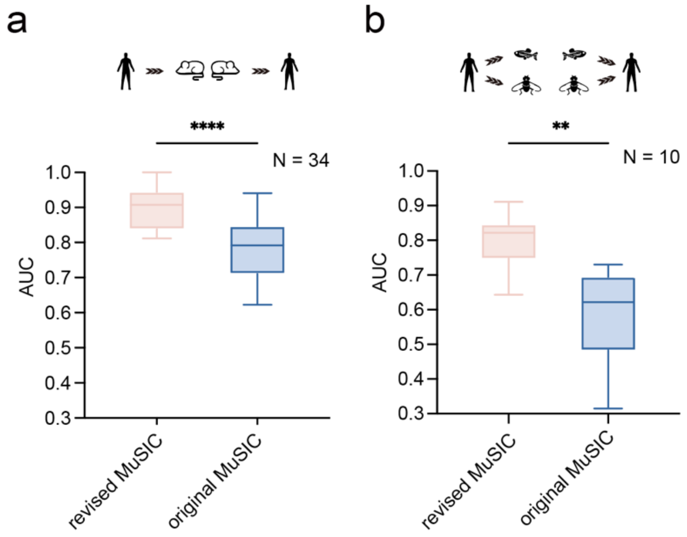

Figure R16 Revised MuSIC shows a significant performance improvement on cross-species tasks.

(a) Boxplot showing the performance for closely related species of the revised MuSIC (red) and the original MuSIC (blue). **** $P < 0.001$, Wilcoxon test. (b) Boxplot showing the performance for distantly related species of the revised MuSIC (red) and the original MuSIC (blue). ** $P < 0.01$, Wilcoxon test.

Excerpt (main text)

We replaced **Fig. R14a** with **Fig. 2c**, **Fig. R14b** with **Supplementary Fig. 3a** and **Fig. R14c** with **Supplementary Fig. 3b**. And we added relevant descriptions to the **Results** (L230 – L234, L262 – L269 and L316 – L328) section:

Results (L230 – L234):

> MuSIC model combines the learning RNA module and learning interaction module to capture and enhance RBP–RNA interaction features learning. The learned RBP–RNA representations were then integrated using the multilayer perceptron

(MLP) classifier to generate the prediction outputs (**Fig. 2b,c** and **Supplementary Fig. 3a,b**; see **Methods** for details).

Results (L262 – L269):

The protein and RNA foundation models trained on large-scale unlabeled datasets provide potential binding features for RBP and RNA [45–48]. We incorporated protein foundation model (ProtT5 [48]) together with RNA foundation model (RiNALMo [46]) to extract high-dimensional representations for both RBPs and RNAs. Incorporating the ProtT5-derived RBP embeddings could improve the average AUC from 0.85 to 0.86. Furthermore, incorporating RiNALMo-derived RNA embeddings could lead to an additional improvement of average AUC from 0.86 to 0.91 (**Fig. 2f**; see **Methods** for details).

Results (L316 – L328):

We then evaluated the prediction accuracy of MuSIC and other computational methods in the cross-species prediction across 44 datasets from human, mouse, zebrafish, and fly. MuSIC outperformed other methods, showing an obvious improvement of in AUC (average AUC: MuSIC = 0.91, PaRPI = 0.58, HDRNet = 0.72, PrismNet = 0.70, DeepBind = 0.68, GraphProt = 0.71; **Fig. 3a**), as well as in the accuracy (ACC) and Area Under the Precision-Recall Curve (PRAUC) metrics (**Supplementary Fig. 4b-d**). In addition, we found that MuSIC achieves high accuracy for predicting RBP–RNA interactions in both the closely related predictions (e.g., human to mouse) and distantly related predictions (e.g., human to zebrafish or fly) (**Supplementary Fig. 4e,f**). For example, MuSIC showed AUCs of 0.95 for YTHDC2 (mouse) and 0.91 for Fmr1 (fly) in the cross-species prediction, a significant improvement than other computational methods (**Fig. 3b** and **Supplementary Fig. 5a**).

Reviewer #3 comment 3

The label smoothing strategy, though effective, is conceptually incremental; applying conservation-based weighting is interesting but not a major methodological advance. Furthermore, the paper does not compare its approach against alternative domain adaptation or transfer learning frameworks (e.g., adversarial alignment, contrastive pretraining, or large pretrained RNA foundation models). Architecturally, the model design (CNN, residual, SE modules) is standard and similar to existing frameworks such as PrismNet, HDRNet, and DeepCLIP.

RESPONSE:

Thanks for your valuable suggestion. In response, we have carefully considered the framework design of MuSIC, and implemented the following improvements.

The domain adaptation approaches include the supervised domain adaptation (SDA) and unsupervised domain adaptation (UDA). However, the SDA approaches were not suitable for the cross-species task because the data labels were unavailable in the target species, which would prevent the fine-tuning of the model trained on the source species. Here, we explored the application of the UDA approaches, which could replace the label smoothing strategy. We compared the prediction performance based on several different UDA approaches, including Adversarial Discriminative Domain Adaptation (ADDA), ADDA_reverse, Domain-Adversarial Training of Neural Networks (DANN), and Adversarial Unsupervised Domain Adaptation with Conditional and Label Shift (CLS). We show that the framework architecture with label smoothing strategy can outperform those with the UDA approaches (**Fig. R17**). These results confirm that label smoothing strategy provides better performance than UDA, and this optimal transfer learning architecture is suitable for the cross-species prediction task.

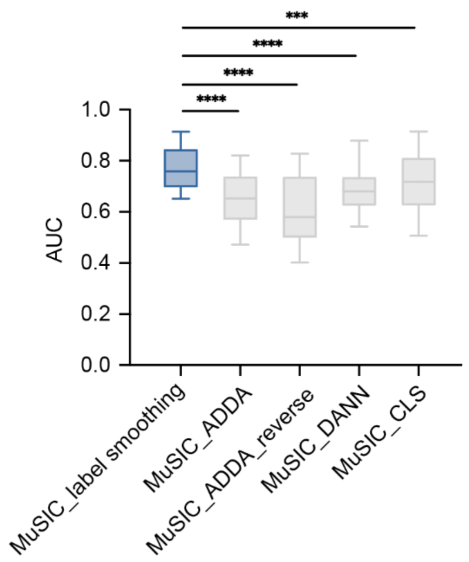

Figure R17 The label smoothing strategy outperforms DA in cross-species tasks

Boxplot showing the performance of the MuSIC with label smoothing (blue) and four UDA methods (grey). *** $P < 0.005$, **** $P < 0.001$, Wilcoxon test.

In addition, we compared prediction performance of MuSIC with large-scale pre-trained RNA foundation models, and constructed the revised version of MuSIC by integrating the RBP and RNA embeddings from protein and RNA foundation models into the computational framework (refer to **RESPONSE** to **Reviewer #3 comment 2**). By applying the revised version of MuSIC, we observed substantial performance improvements in the cross-species tasks, particularly for the distantly related species (**Fig. R15,16**). We demonstrated that the revised version of MuSIC outperformed multiple transfer learning frameworks, foundation models (**Fig. R18**) and computational methods (**Fig. R19**) in both within-species and cross-species tasks. We greatly appreciate your valuable suggestion, which led to the revised version of MuSIC with significantly enhanced prediction performance.

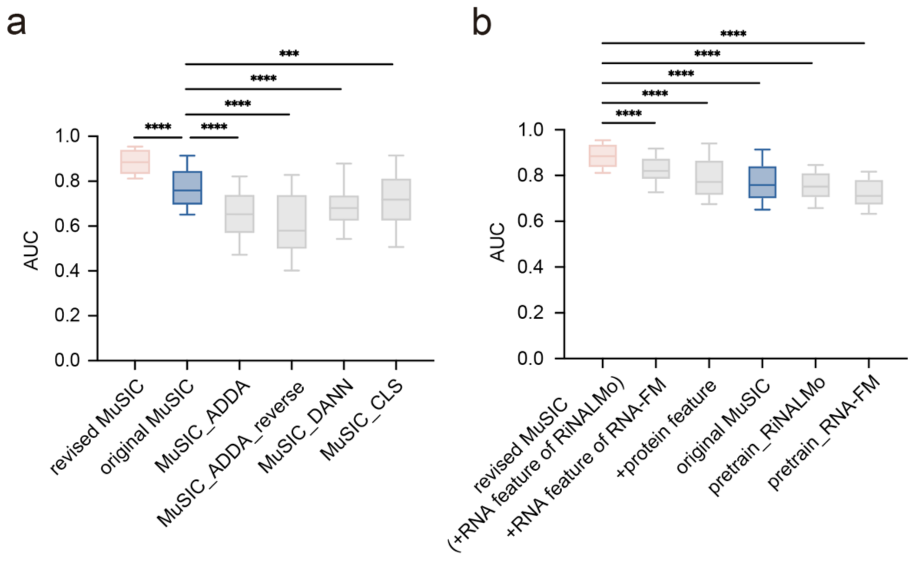

Figure R18 Revised MuSIC outperforms multiple transfer learning frameworks on cross-species tasks

(a) Boxplot showing the performance of the revised MuSIC (red), the original MuSIC (blue) and multiple transfer learning frameworks (grey). (b) Boxplot showing the performance of the revised MuSIC (red), the original MuSIC (blue) and multiple foundation models (grey). *** $P < 0.005$, **** $P < 0.001$, Wilcoxon test.

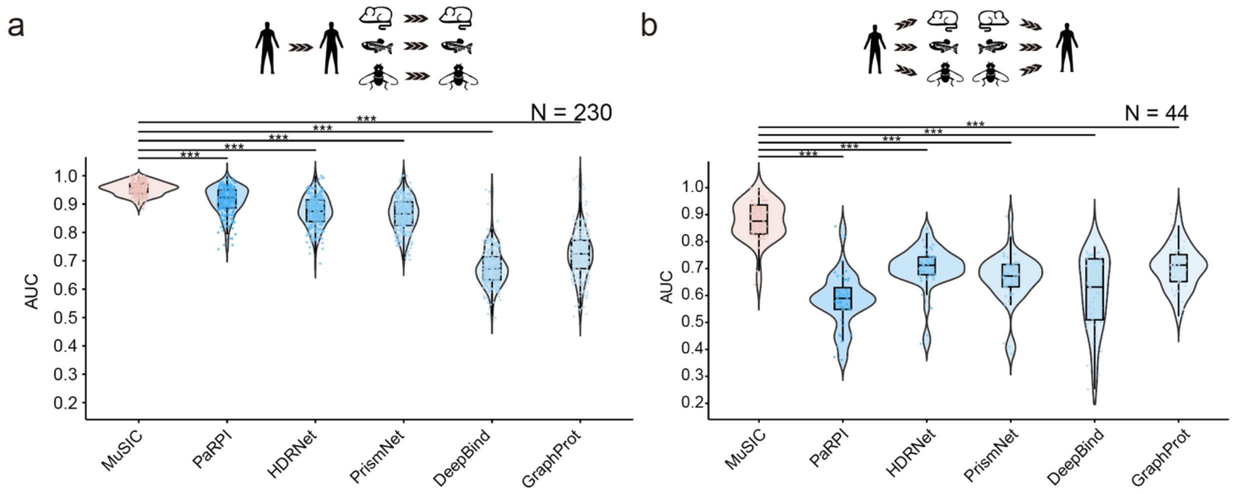

Figure R19 Revised MuSIC outperforms computational methods on both within-species and cross-species prediction tasks

(a) Violin plot showing the prediction accuracy of MuSIC (red) and other computational methods (blue) for 230 within-species datasets (b) Violin plot showing the prediction accuracy of MuSIC (red) and other computational methods (blue) for 44 cross-species datasets. *** $P < 0.005$, Wilcoxon test.

Excerpt (main text)

We replaced **Fig. R18a** with **Supplementary Fig. 3i**, **Fig. R18b** with **Supplementary Fig. 3j**, **Fig. R19a** with **Supplementary Fig. 4a** and **Fig. R19b** with **Supplementary Fig. 4d**. And we added relevant descriptions to the **Results** (L283 – L288 and L308 – L325) and the **Methods** (L897 – L900) sections:

Results (L283 – L288):

> The classification performance of MuSIC's frameworks was benchmarked against diverse transfer learning frameworks, foundation models, machine learning and deep learning classifiers using the identical input features. We found that MuSIC consistently outperformed all the benchmarks, confirming the robustness and effectiveness of MuSIC in modeling RBP binding across species (**Supplementary Fig. 3i-l**).

Results (L308 – L325):

> Briefly, we performed a systematic comparison of prediction accuracy with five existing state-of-the-art computational methods, including PaRPI [32], HDRNet [31], PrismNet [30], DeepBind [28], and GraphProt [26]. As expected, MuSIC showed better prediction accuracy than other methods for the within-species prediction (average AUC: MuSIC = 0.95, PaRPI = 0.91, HDRNet = 0.88, PrismNet = 0.86, DeepBind = 0.67, GraphProt = 0.72) (**Supplementary Fig. 4a**).
>
> We then evaluated the prediction accuracy of MuSIC and other computational methods in the cross-species prediction across 44 datasets from human, mouse, zebrafish, and fly. MuSIC outperformed other methods, showing an obvious improvement of in AUC (average AUC: MuSIC = 0.91, PaRPI = 0.58, HDRNet = 0.72, PrismNet = 0.70, DeepBind = 0.68, GraphProt = 0.71; **Fig. 3a**), as well as in the accuracy (ACC) and Area Under the Precision-Recall Curve (PRAUC) metrics (**Supplementary Fig. 4b-d**). In addition, we found that MuSIC achieves high accuracy for predicting RBP–RNA interactions in both the closely related predictions

> (e.g., human to mouse) and distantly related predictions (e.g., human to zebrafish or fly) (**Supplementary Fig. 4e,f**).

Methods (L897 – L900):

> (iii) transfer learning frameworks: we compared the proposed label smoothing strategy with domain adaptation approaches [90–93]. (iv) pre-trained models: we constructed the models using embeddings derived from different RNA foundation models [45,46].

Overall response to Reviewer #3

Thanks for your constructive and insightful comments. In response to these suggestions, we substantially revised the MuSIC framework by incorporating large-scale pre-trained protein and RNA foundation models, and by integrating foundation model representations with multi-scale convolutional and transformer-based contextual modeling. This architecture empowered with foundation models leads to significant prediction performance improvements in the cross-species tasks, particularly for the evolutionarily distant species where the original model exhibited limited generalization. Systematic benchmarking demonstrates that the revised version of MuSIC can consistently outperform other existing computational methods for both within-species and cross-species tasks. Accordingly, we have updated all the relevant results in the main figures (**Fig. 2-6**) and supplementary materials (**Supplementary Fig. 3-13 and Supplementary Table 2-8**) based on the results from the revised version of MuSIC. We also provided detailed description and discussion in the revised manuscript. Notably, the revised version of MuSIC enables the identification of evolutionarily conserved *de novo* RBP-binding motifs, which were further experimentally validated (**Fig. 4c**). We once again thank you for your constructive suggestions, which helped us significantly improve our MuSIC model.

Point-to-Point response to Reviewer #4

Reviewer #4: Accurately modeling RNA–protein interactions remains a central challenge in computational genomics. Reliable prediction of RBP binding is essential for understanding post-transcriptional regulation and the impact of non-coding variants. He et al propose a new deep learning based model to predict RBP-RNA interactions across species. Their model is based on a CNN which utilizes conservation-based label smoothing. They perform evaluations that include within-species binding prediction, cross-species binding prediction as well as SNV effect prediction. The method is well-explained and the figures are clear.

RESPONSE:

Thanks for your positive and thoughtful evaluations on our work. We appreciate your recognition of the importance of accurately modeling RNA–protein interactions and of the challenges associated with cross-species RBP binding prediction. As you summarized, MuSIC leverages a deep learning framework with conservation-aware label smoothing to enable robust RBP–RNA interaction prediction across species, and we systematically evaluated its performance in within-species, cross-species, and SNV effect prediction tasks.

In response to your suggestions, we have conducted additional analyses, including comparisons with a motif-based baseline and the incorporation of foundation models to encode high-dimensional features, rather than relying on trivial sequence homology. We also examined model training and validation balance, addressing data splitting, threshold selection, and feature considerations. We have carefully revised the manuscript and provided detailed point-by-point responses below.

Reviewer #4 comment 1

The authors first demonstrate that evolutionarily conserved RBPs exhibit similar RNA-binding preferences. While this analysis is useful, it is somewhat repetitive, as previous studies have already reported similar patterns (e.g., Ray et al., 2013).

RESPONSE:

Thanks for your helpful comment. Previous studies (e.g., Ray et al., 2013) have reported that evolutionarily conserved RBPs often exhibit similar RNA-binding preferences. In our study, we systematically examined the conservation degree of RBPs and RBP-binding preference, and then incorporated conservation information of RBPs into the computational framework of MuSIC, which could enable cross-species RBP–RNA interaction prediction in the absence of CLIP data.

Previous studies primarily focused on characterizing the conservation patterns at the protein sequence and RNA motif levels (e.g., Ray et al., 2013). With recent advancements in protein structure prediction and the increasing availability of protein annotation data, we predicted protein structures using AlphaFold2 and performed a comprehensive analysis of both full-length proteins and domains, examining their sequence and structural similarities based on domain annotations. By analyzing the protein sequences and structures of 216 RBPs from 11 species ranging from human to yeast, we assessed which protein-level conservation features could provide robust results for cross-species transferring. The results indicate that the protein sequence exhibit higher conservation for the core RBDs compared to the full-length RBPs, while the structural similarity for both core RBDs and full-length RBPs remain relatively high across species (**Fig. R20**).

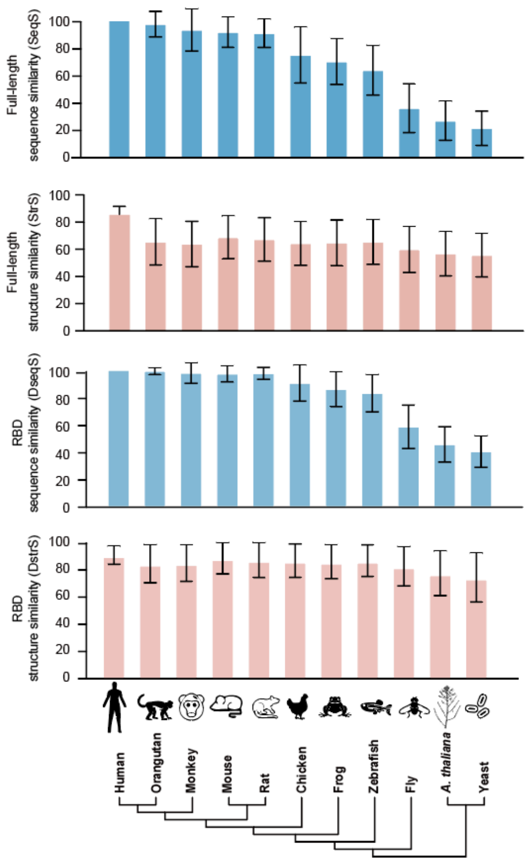

Figure R20 Evolutionary conservation of RBPs across 11 species.

Bar plots showing the sequence and structural similarity of RBPs across 11 species, in terms of full-length sequence (SeqS in blue), full-length structure (StrS in red), RBD sequence (DseqS in blue) and RBD structure (DStrS in red). The phylogenetic tree is constructed based on the 18S rRNA sequences in the 11 species, and shows the relative relationships between human and non-human species.

Additionally, we explored the conservation of RNA patterns from multiple perspectives, including the distribution of RBP-binding positions, motif similarities, and RBP–RNA interaction residues in 3D space. Consistent with previous reports of evolutionary gradients, RBPs with conserved sequence and structural features also exhibit conserved RNA patterns (Fig. R21), supporting the feasibility of transferring binding information from the source species to the target species. Together, these conservation analyses provide biological rationale underlying the computational framework of MuSIC, instead of reiterating established biological findings.

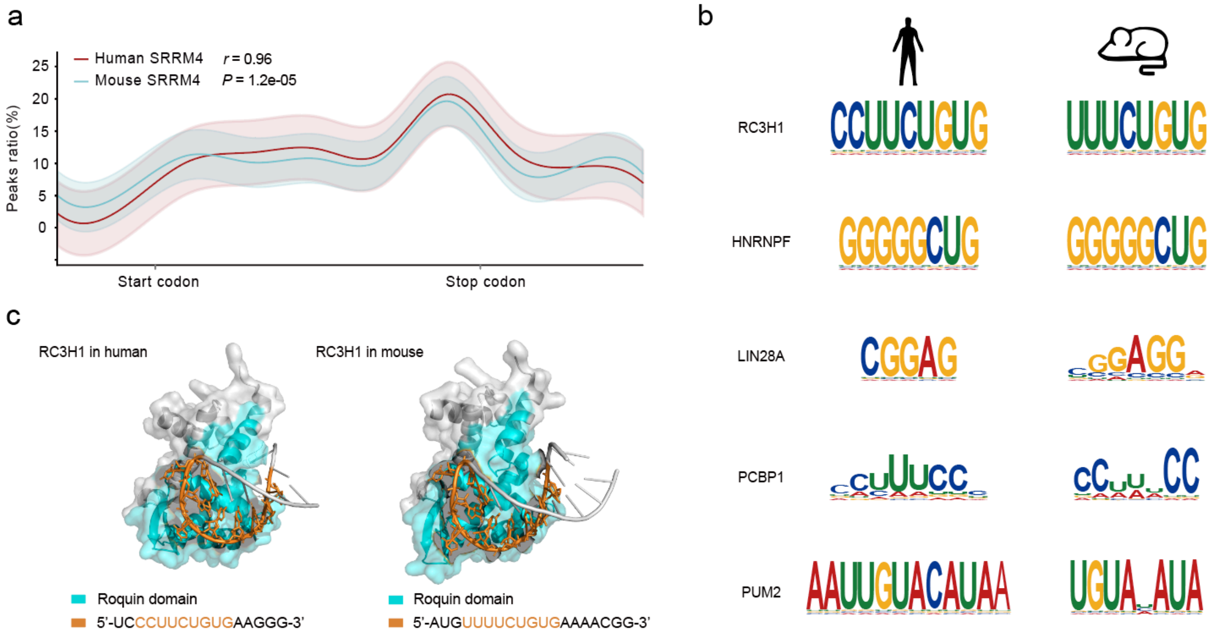

Figure R21 Evolutionary conservation of RBP-targeted RNAs.

(a) SRRM4 binding distribution along meta-transcript in human (red line) and mouse (cyan line). (b) Binding motif alignment of homologous RBPs in human (left) and mouse (right). (c) Three-dimensional structure of RC3H1 in human (PDB ID 4QIL, left) and mouse (PDB ID 4QI2, right), showing bound RNA residues (orange), RBDs residues (blue), and other remaining RNA and protein residues (grey).

Reviewer #4 comment 2

Regarding the performance of MuSIC, it's important to note that MuSIC's within-species prediction is only slightly better than HDRNet and PrismNet (0.88 vs 0.87 and 0.86). MuSIC's performance in cross-species prediction of binding is much superior than other methods. However, a key concern here is that the model's higher performance in closely related species may be largely driven by trivial sequence homology, not true cross-species generalizability. Because conservation signals are explicitly incorporated into the model's label smoothing strategy, the reported improvements in closely related species may reflect circular reasoning. The model is evaluated on its ability to recover conserved binding sites, but conservation itself is already provided as an input. The authors should eloborate on this. How would a simple baseline scanning model using the same motif (as PWM or PFM9) weighted by conservation values score?

RESPONSE:

Thanks for your thoughtful comment. MuSIC was developed to focus on RBP–RNA interaction prediction for the cross-species tasks rather than for the within-species tasks, where conservation information provides limited additional benefit. Although we did not expect obvious prediction performance improvement over existing CNN-based baselines such as HDRNet and PrismNet in the within-species tasks, MuSIC still achieves slightly higher average AUC score (0.88 vs 0.87 and 0.86) than existing methods in the within-species prediction.

As discussed above (refer to **Reviewer #3 Comment 2**), we developed the revised version of of MuSIC by integrating protein and RNA foundation model embeddings, which showed obvious prediction performance improvement over the original CNN-based version. The revised framework combines high-dimensional representations derived from foundation models with multi-scale convolutional feature extraction and transformer-based contextual modeling. Importantly, the revised version of MuSIC outperforms existing computational methods in both within-species (average AUC: MuSIC = 0.95, PaRPI = 0.91, HDRNet = 0.88, PrismNet = 0.86, DeepBind = 0.67, GraphProt = 0.72; **Fig. R22a**) and cross-species tasks (average AUC: MuSIC = 0.91, PaRPI = 0.58, HDRNet =

0.72, PrismNet = 0.70, DeepBind = 0.68, GraphProt = 0.71; **Fig. R22b**). Notably, we included PaRPI, a recently published tool for within-species tasks, in the performance evaluation.

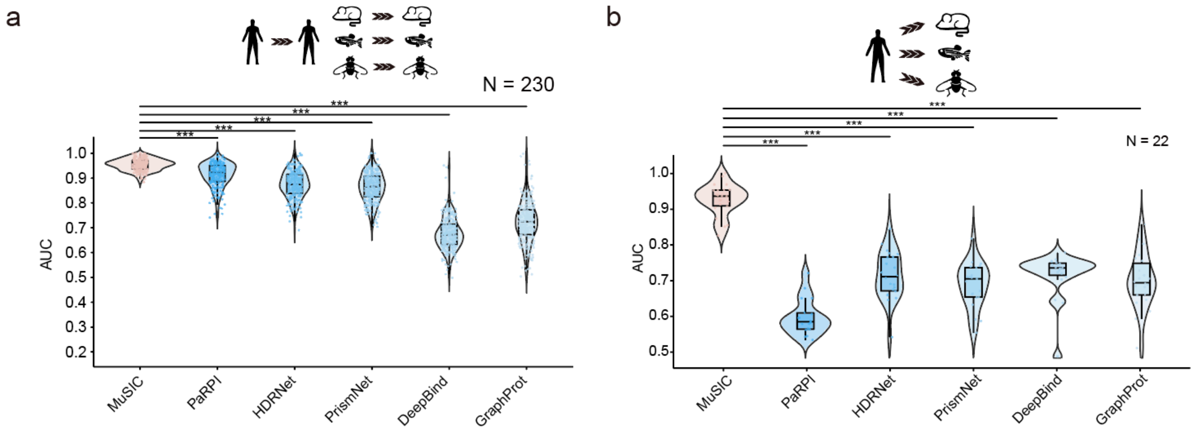

Figure R22 The revised MuSIC outperforms other methods in within- and cross-species prediction

(a) Violin plot showing the prediction accuracy of MuSIC (red) and other computational methods (blue) for 230 within-species datasets. *** $P < 0.005$, Wilcoxon test. (b) Violin plot showing the prediction accuracy of MuSIC (red) and other computational methods (blue) for 22 cross-species datasets. *** $P < 0.005$, Wilcoxon test.

We agree with the hypothesis that the original version of MuSIC's performance gains in the cross-species tasks may arise from trivial sequence homology or circular use of conservation signals. In response, we constructed a simple motif-scanning baseline to test this hypothesis. Specifically, we derived the position weight matrices (PWMs) weighted by conservation scores from the source-RBPs, and scanned the target-species sequences to predict the binding sites. Importantly, this motif-scanning baseline strategy performs worse than the original version of MuSIC in the cross-species tasks (**Fig. R23**), suggesting that MuSIC's performance cannot be attributed to a trivial recovery of conserved sites or to circular use of conservation information.

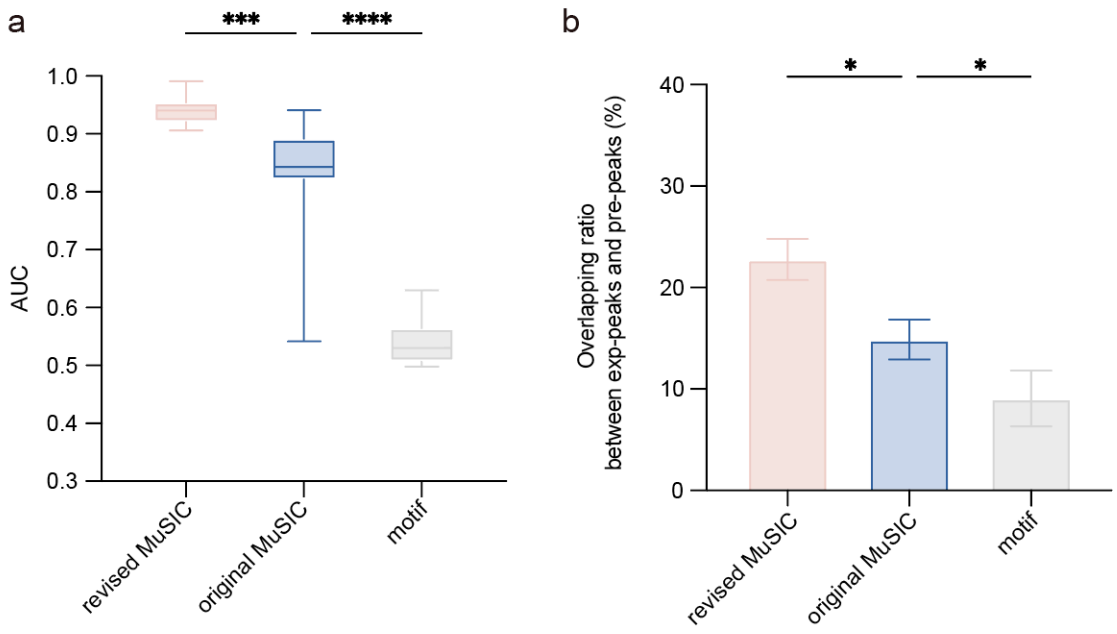

Figure R23 The MuSIC outperforms motif-based baseline in cross-species prediction

(a) Boxplot showing the performance of the revised version of MuSIC (red), the original version of MuSIC (blue), and motif-based baseline (grey) across 13 cross-species datasets. *** $P < 0.005$, **** $P < 0.001$, Wilcoxon test. (b) Bar plot showing the overlapping ratio between the experimentally-derived and predicted peaks by the revised version of MuSIC (red), the original version of MuSIC (blue), and motif-based baseline (grey) across 13 RBPs. * $P < 0.05$, Wilcoxon test.

After incorporating the protein and RNA foundation models into the computational framework, the revised version of MuSIC not only showed improved prediction performance in within-species tasks (average AUC score from 0.88 to 0.95) and in the closely related species (average AUC score from 0.79 to 0.89), but also demonstrated substantial performance gains in the evolutionarily distant species, e.g., zebrafish and fly (average AUC score from 0.58 to 0.81) (**Fig. R24**). To summarize, the revised version of MuSIC exhibit strong power for the cross-species generalization, especially in the evolutionarily distant species, which cannot simply benefit from homology sequence and trivial conservation signals.

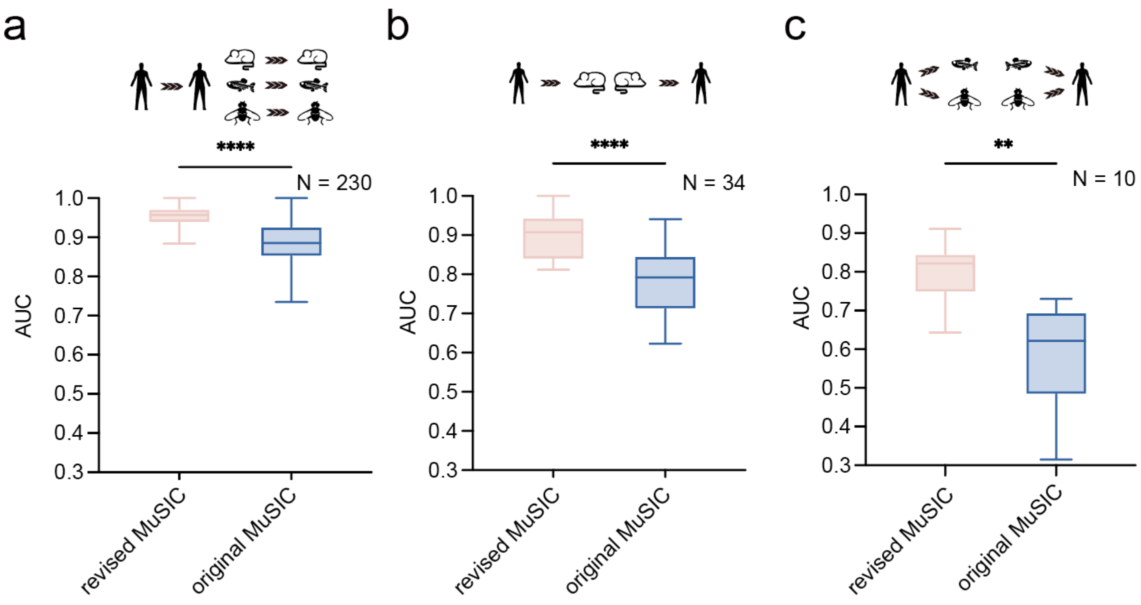

Figure R24 The revised MuSIC outperforms original MuSIC in distantly species prediction

(a) Boxplot showing the within-species performance of the revised version of MuSIC (red) and the original version of MuSIC (blue). **** $P < 0.001$, Wilcoxon test. (b) Boxplot showing the performance for closely related species of the revised MuSIC (red) and the original MuSIC (blue). **** $P < 0.001$, Wilcoxon test. (c) Boxplot showing the performance for distantly related species of the revised MuSIC (red) and the original MuSIC (blue). ** $P < 0.01$, Wilcoxon test.

Excerpt (main text)

We replaced **Fig. R22a** with **Supplementary Fig. 4a** and **Fig. R22b** with **Fig. 3a**. And we added relevant descriptions to the **Results** (L308 – L325 and L334 – L341) section:

Results (L308 – L325):

> Briefly, we performed a systematic comparison of prediction accuracy with five existing state-of-the-art computational methods, including PaRPI [32], HDRNet [31], PrismNet [30], DeepBind [28], and GraphProt [26]. As expected, MuSIC showed better prediction accuracy than other methods for the within-species prediction (average AUC: MuSIC = 0.95, PaRPI = 0.91, HDRNet = 0.88, PrismNet = 0.86, DeepBind = 0.67, GraphProt = 0.72) (**Supplementary Fig. 4a**).

> We then evaluated the prediction accuracy of MuSIC and other computational methods in the cross-species prediction across 44 datasets from human, mouse, zebrafish, and fly. MuSIC outperformed other methods, showing an obvious improvement of in AUC (average AUC: MuSIC = 0.91, PaRPI = 0.58, HDRNet = 0.72, PrismNet = 0.70, DeepBind = 0.68, GraphProt = 0.71; **Fig. 3a**), as well as in the accuracy (ACC) and Area Under the Precision-Recall Curve (PRAUC) metrics (**Supplementary Fig. 4b-d**). In addition, we found that MuSIC achieves high accuracy for predicting RBP–RNA interactions in both the closely related predictions (e.g., human to mouse) and distantly related predictions (e.g., human to zebrafish or fly) (**Supplementary Fig. 4e,f**).

Results (L334 – L341):

> Finally, we compared the prediction accuracy of MuSIC and other computational methods by evaluating to what degree the RBP-binding peaks from CLIP-seq experiments could be rediscovered (see **Methods** for details). Briefly, the model was trained based on the RBP-binding peaks in human, followed by predicting peaks across all the transcripts in mouse (for 22 RBPs), zebrafish (for 1 RBP) and fly (for 3 RBPs) (**Supplementary Table 4**). For each RBP, we observed that MuSIC showed consistently better performance than other computational methods (**Fig. 3d** and **Supplementary Fig. 5d**), particularly on the cases of FUS and CELF2 (**Fig. 3e**).

Reviewer #4 comment 3

The authors have to explain the effect of sequence length on performance. How is it possible that using ~100nt and ~400 nt as input sequence length can give random prediction results (i.e. AUCs close to 0.5)? If structure features were included one could argue that it's due to the local folding of RNA molecule, but here it is not the case.

RESPONSE:

Thanks for your constructive suggestion. We agree that it is necessary to make it clear why the prediction performance using 101nt and 400nt sequences just approaches random prediction results in the absence of structural features. Upon reviewing our previous analyses, we determined that this issue was due to insufficient optimization of the 101nt and 400nt configurations. With optimized hyperparameters, the average AUC score in the cross-species tasks showed an increase from 0.55 to 0.65 using 101nt, as well as from 0.55 to 0.64 using 400nt. Both 101nt and 400nt configurations exhibited worse prediction performance than 200nt (average AUC score = 0.75; **Fig. R25**). These results indicate that 101nt and 400nt fail to capture sufficient transferable contextual information required for robust cross-species generalization.

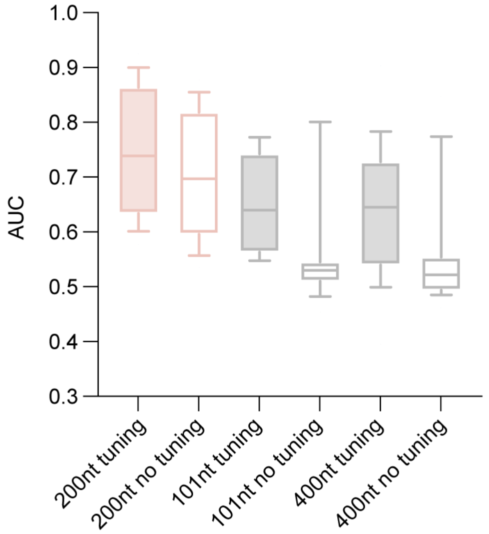

Figure R25 Effect of hyperparameter optimization on cross-species prediction performance

Boxplot showing the performance of MuSIC in cross-species prediction w/o hyperparameter optimization across input sequence lengths (101, 200 and 400nt).

We also extended the ablation analyses to cover a broader range of input sequence lengths, including 101nt, 200nt, 300nt, 400nt, 500nt, 1000nt and 2000nt, allowing a systematic evaluation. We found that, in the cross-species setting, MuSIC could achieve the best prediction performance under the input length of 200nt (**Fig. R26a**). Notably, extending the contextual window size did not improve the prediction performance, instead leading to declined prediction performance. We also performed the same analysis in the within-species setting, and found that MuSIC could achieve the best prediction performance under the input length of 101nt, while 200nt showing a comparable prediction performance (**Fig. R26b**). Given the primary objective of addressing the cross-species generalization issue, we finally selected 200nt as the default input sequence length in MuSIC.

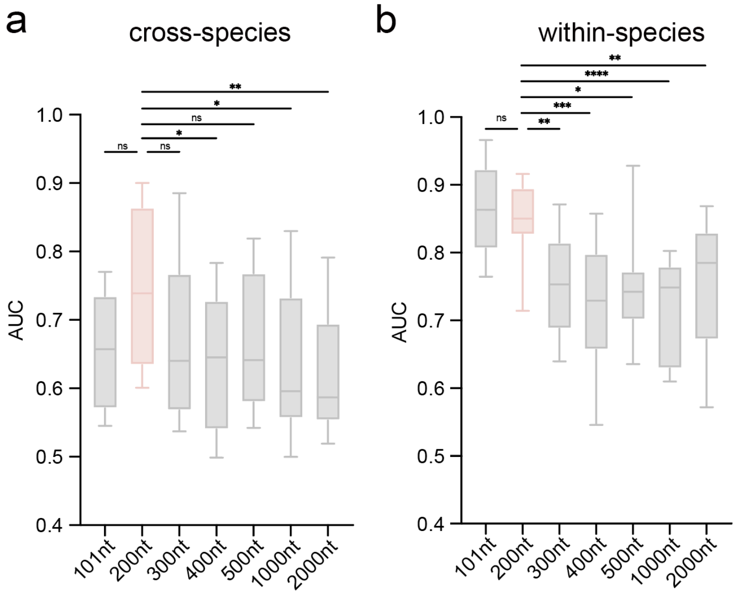

Figure R26. Performance comparison across different input sequence lengths

Boxplot showing the performance of MuSIC using different input sequence lengths in (a) cross-species and (b) within-species tasks. ns $P > 0.05$, * $P < 0.05$, ** $P < 0.01$, *** $P < 0.005$, **** $P < 0.001$, Wilcoxon test.

Excerpt (main text)

We replaced **Fig. R26a** with **Supplementary Fig. 3c** and **Fig. R26b** with **Supplementary Fig. 3d.** And we added the relevant descriptions to the **Results** (L242 – L251), the **Discussion** (L532 – L538) and the **Methods** (L891 – L893) sections:

Results (L242 – L251):

> We found that increasing the input sequence length to 200nt could significantly improve the Area Under the Curve (AUC) score, reaching 0.75 in cross-species prediction compared to sequences of lengths 101nt, 300nt, 400nt, 500nt, 1000nt, and 2000nt (**Fig. 2f**, **Supplementary Fig. 3c** and **Supplementary Table 3**). We also performed the same analysis in the within-species setting, and found that MuSIC could achieve the best prediction performance under the input length of 101nt, while 200nt showing a comparable prediction performance (**Supplementary Fig. 3d**). Given the primary objective of addressing the cross-species generalization issue, we selected 200nt as the default input sequence length in MuSIC.

Discussion (L532 – L538):

> In our study, we revealed that increasing the input sequence length from 101nt to 200nt could significantly improve the prediction performance across species (AUC from 0.65 to 0.75). In contrast, further increasing the sequence length beyond 200nt, including 300nt, 400nt, 500nt, 1000nt, and 2000nt, led to a consistent decline in model performance. Increasing the input sequence length can not only provide more useful sequence information, but also may include noisy and redundant sequence information for modeling RBP binding.

Methods (L891 – L893):

> (i) input sequence length: we tried different input sequence lengths of 101nt, 200nt, 300nt, 400nt, 500nt, 1000nt and 2000nt.

Reviewer #4 comment 4

In rediscovering the peaks from CLIP-seq experiments (Pg 11), 0.9 threshold is used to classify a prediction as a hit. This threshold is chosen in an ad-hoc manner and may not be an optimal threshold value for other methods. It would be interesting to see how the other methods perform when the top X ranked predictions are compared to experimentally derived peaks where X is a fixed number.

RESPONSE:

Thanks for your valuable suggestion. We agree that using a single probability threshold (e.g., 0.9) may appear ad hoc and cannot provide a fair comparison across different computational methods. In response, we presented the top X ranked predictions for each method, where X was set to (5,000, 10,000, 20,000, 50,000, 100,000). For each X value, we calculated the overlapping ratio between experimentally derived CLIP-seq peaks and the predicted peaks. We found that increasing the X value could lead to higher overlapping ratio across all methods (**Fig. R27**). However, we hypothesized that selecting high-confidence RBP–RNA interaction pairs based on top X ranked predictions would not be the optimal approach for predicting RBP–RNA interactions. The selection process can be influenced by the distribution of predicted scores across datasets of varying sizes, which can result in inconsistencies in the classification of the same RNA sequence as a high-confidence binder depending on the data distribution.

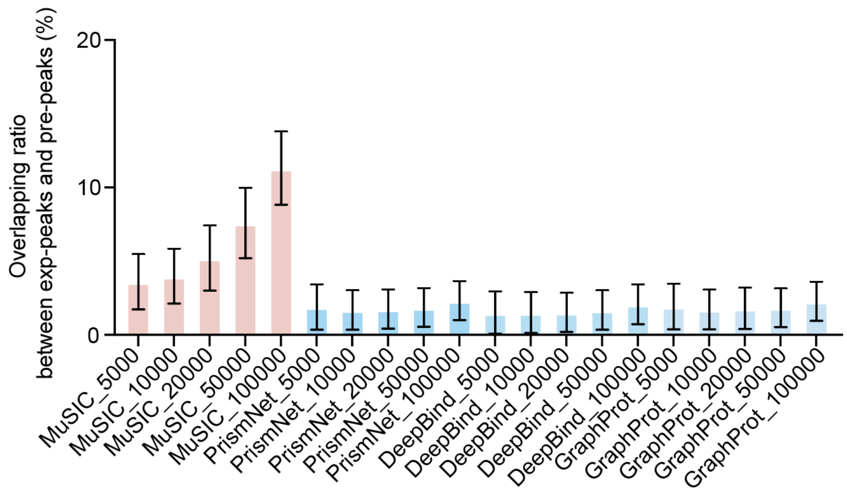

Figure R27 MuSIC outperforms other computational methods across multiple top X ranked predictions

Bar plot showing the overlapping ratio between the experimentally-derived and predicted peaks by MuSIC (red) and other computational methods (blue) across multiple top X ranked predictions.

Here, we decided to use a fixed threshold to select the high-confidence RBP–RNA interactions, ensuring consistent classification of the same sequence, regardless of input distribution. Moreover, this threshold-based method has been widely used by existing RBP–RNA interaction prediction models, such as HDRNet and PrismNet. Briefly, we reformulated the analyses using a threshold-based approach with different prediction thresholds, i.e., 0.9, 0.8, 0.7, and 0.6. We found that lowering the threshold resulted in an increased overlapping ratio for all computational methods, and MuSIC exhibited a higher overlapping ratio than all the other computational methods under all the thresholds (**Fig. R28**).

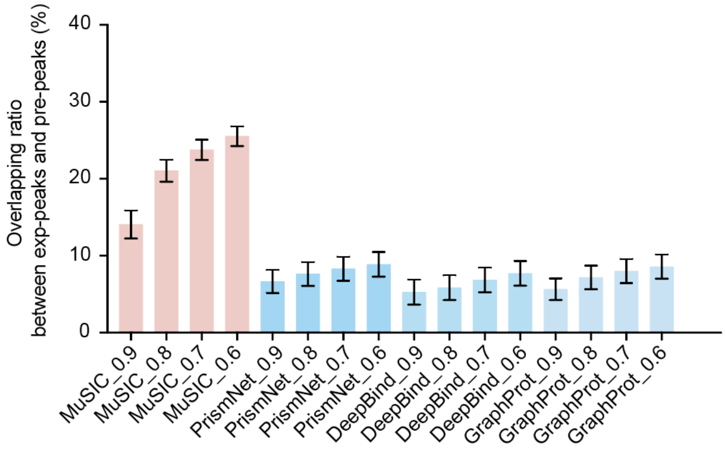

Figure R28 MuSIC outperforms other methods across multiple prediction thresholds

Bar plot showing the overlapping ratio between the experimentally-derived and predicted peaks by MuSIC (red) and other computational methods (blue) across multiple prediction thresholds.

Excerpt (main text)

We replaced **Fig. R28** with **Supplementary Fig. 5d**. And we added relevant descriptions to the **Methods** (L920 – L924) section:

Methods (L920 – L924):

> We then estimated the prediction performance of the computational methods based on the overlap between the predicted peaks with binding probabilities above 0.8 and the experimentally-derived peaks. To further assess the robustness of this evaluation, we systematically tested multiple prediction thresholds (0.9, 0.8, 0.7, and 0.6) and examined their effects on the degree of overlap.

Reviewer #4 comment 5

It could be valuable to have similar GC-content in positive and negative sequences. Can the authors comment on whether they take into account this?

RESPONSE:

Thanks for raising this important issue. We agree that comparable GC-content between positive and negative sequences is critical to ensure that model performance is not driven by trivial sequence composition differences. In response, we computed the GC-content distributions for both positive and negative sequences across multiple cross-species training datasets, as well as validation datasets. The positive and negative samples showed comparable GC-content distribution, indicating that the GC-content cannot be the key confounding factor in either model training or evaluation (**Fig. R29**).

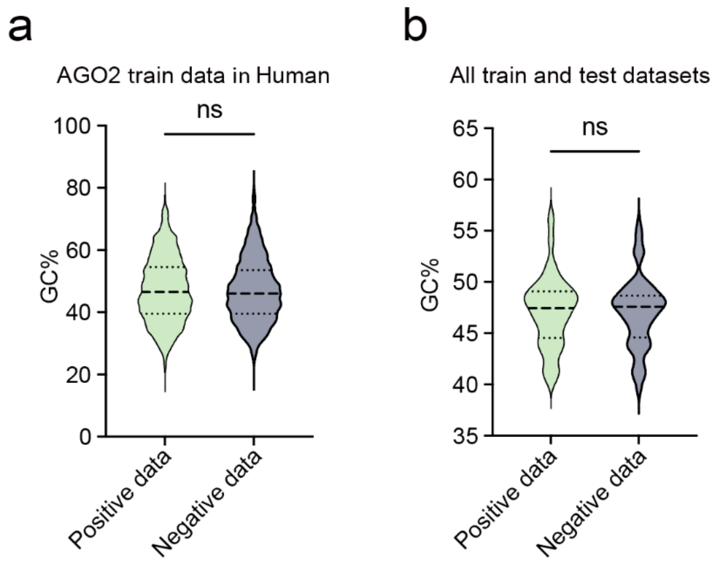

Figure R29 GC-content distributions are consistent between positive and negative samples

(a) Violin plot showing the GC-content distributions of positive (green) and negative (grey) samples in the human AGO2 training dataset. (b) Violin plot showing the mean GC-content of positive and negative samples across 44 cross-species datasets. ns, $P > 0.05$, Wilcoxon test.

Excerpt (main text)

We replaced **Fig. R29a** with **Supplementary Fig. 3m** and **Fig. R29b** with **Fig. 3n**. And we added relevant descriptions to the **Results** (L290 – L294) section:

Results (L290 – L294):

> In addition, we computed the GC-content distributions for both positive and negative sequences across multiple cross-species datasets. The positive and negative samples showed comparable GC-content distribution, indicating that the GC-content cannot be the key confounding factor in either model training or evaluation (**Supplementary Fig. 3m,n**).

Reviewer #4 comment 6

In the cross-species prediction task, it is unclear why the authors use a 4:1 split between training and validation. Since the training and test datasets come from entirely separate species (e.g., human vs. mouse), this is not a traditional cross-validation setting. It is therefore unclear why the authors subsample the mouse AGO2 iCLIP dataset to create a test set with only 34 positive and 60 negative sequences. A more straightforward approach would be to use the entire mouse dataset as the test set. At minimum, if subsampling is necessary, the authors should repeat the procedure multiple times with different random seeds and report the average and variability of the results to ensure robustness.

RESPONSE:

Thanks for this thoughtful comment regarding the data splitting strategy in the cross-species tasks. We agree that cross-species prediction does not correspond to a traditional cross-validation setting, as training and testing are performed on entirely distinct species. To assess whether the choice of split ratio between training and validation affects evaluation results, we systematically examined different split ratios in the cross-species setting, including 4:1, 2:1, 1:1, 1:2, and 1:4. For instance, when setting the split ratio as 4:1, the training set contains 10,000 sequences while the corresponding validation set contains 2,500 sequences. Thus, the different ratios correspond to validation set sizes of 2,500; 5,000; 10,000; 20,000 and 40,000 sequences, respectively.

For 11 target-species datasets that were insufficient to support certain split ratios, we followed your suggestion and evaluated robustness through repeated subsampling. Specifically, we performed subsampling using three different random seeds, i.e., 40, 50 and 60. For each random seed, 66% of the available target-species data was randomly selected as the validation dataset (average AUC: seed 40 = 0.922, seed 50 = 0.915, seed 60 = 0.915; **Fig. 29a**). The reported AUC score for these RBPs represents the average performance across the AUC obtained from the three different random seeds. We observed that the prediction performance remains highly consistent across all split ratios (average AUC: 4:1 = 0.884, 2:1 = 0.885, 1:1 = 0.886, 1:2 = 0.885, 1:4 = 0.882; **Fig.**

30b). These results suggest that the cross-species prediction performance will not be influenced by the subsampling procedure.

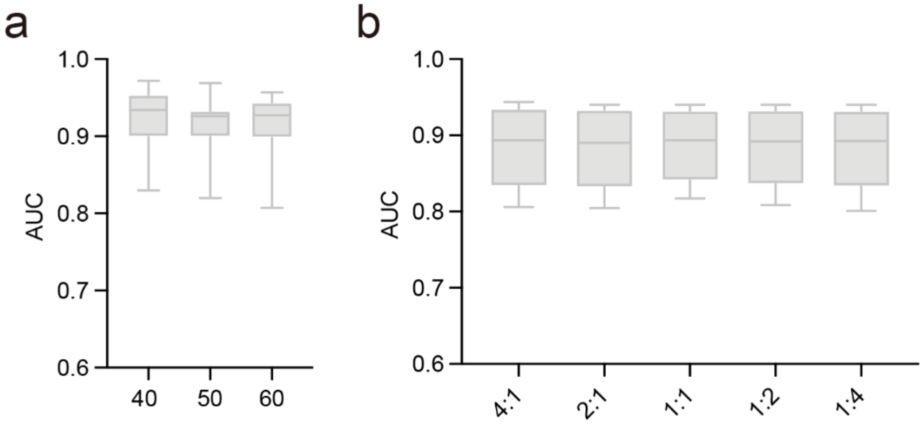

Figure R30 The performance of MuSIC is consistent across different split ratios

(a) Boxplot illustrating the performance of MuSIC using different random seeds for subsampling across 11 cross-species datasets. (b) Boxplot showing the performance of MuSIC with varying split ratios between training and validation in cross-species prediction tasks.

Excerpt (main text)

We replaced Fig. R30b with Supplementary Fig. 3o. And we added relevant descriptions to the Results (L294 – L300) and Methods (L646 – L661) sections:

Results (L294 – L300):

> Because cross-species prediction does not correspond to a traditional cross-validation setting, we further assessed the effect of different training–validation split ratios. As expected, MuSIC exhibited highly consistent performance across a wide range of split ratios (average AUC: 4:1 = 0.884, 2:1 = 0.885, 1:1 = 0.886, 1:2 = 0.885, 1:4 = 0.882), demonstrating that the cross-species prediction performance will not be influenced by the subsampling procedure (Supplementary Fig. 3o; see Methods for details).

Methods (L646 – L661):

We note that cross-species prediction does not correspond to a traditional cross-validation setting, as training and testing are performed on entirely distinct species. To assess whether the choice of split ratio between training and validation affects evaluation results, we systematically examined different split ratios in the cross-species setting, including 4:1, 2:1, 1:1, 1:2, and 1:4. For instance, when setting the split ratio as 4:1, the training set contains 10,000 sequences while the corresponding validation set contains 2,500 sequences. Thus, the different ratios correspond to validation set sizes of 2,500; 5,000; 10,000; 20,000 and 40,000 sequences, respectively.

For target-species datasets that were insufficient to support certain split ratios, we evaluated robustness through repeated subsampling using three different random seeds, i.e., 40, 50 and 60. For each random seed, 66% of the available target-species data was randomly selected as the validation dataset. And the reported AUC score for these RBPs represents the average performance across the AUC obtained from the three different random seeds.

Reviewer #4 comment 7

Line 336.

"Notably, RBP-binding motif prediction failed for certain RBPs due to their weak or undetectable binding signals in the distantly related species, such as yeast, fly, and zebrafish. "It would be valuable to list those within the sentence.

RESPONSE:

Thanks for your kind suggestion. In response, we have revised the MuSIC model, which requires RBP sequence input. During the cross-species motif identification process, we found that the motif prediction failed for six proteins due to the lack of sequence in the UniProt. Specifically, these proteins include APOBEC3C and IGF2BP2 in fly, SDOS in *A. thaliana*, and APOBEC3C, SDOS, and TBRG4 in yeast. We noticed that some motif predictions failed due to insufficient motif enrichment, including HNRNPF in orangutan and monkey, MBNL2 and HNRNPF in fly, HNRNPF and MOV10 in *A. thaliana*, and XPO5, XRN2, NONO, DDX51, and HNRNPF in yeast.

Excerpt (main text)

We added relevant descriptions to the **Results** (L402 – L407) section:

Results (L402 – L407):

> Notably, RBP-binding motif prediction failed for certain RBPs due to the limited availability of protein sequences (e.g., fly: APOBEC3C and IGF2BP2; *A. thaliana*: SDOS; yeast: APOBEC3C, SDOS, and TBRG4), as well as insufficient motif enrichment (e.g., orangutan: HNRNPF; monkey: HNRNPF; fly: MBNL2 and HNRNPF; *A. thaliana*: HNRNPF and MOV10; yeast: XPO5, XRN2, NONO, DDX51, and HNRNPF) (**Fig. 4d** and **Supplementary Fig. 8**).

Line 300. “evolutionarily closed” -> “evolutionarily close”

RESPONSE:

Thanks for pointing out this issue. We have corrected the phrasing to “evolutionarily close” in line 356.

Excerpt (main text)

We added relevant descriptions to the **Results** (L354 – L356) section:

Results (L354 – L356):

> The aforementioned results reveal that MuSIC could generate better cross-species predictions than other computational methods, particularly when the source and target species are more evolutionarily close.

Line 308. The word “quantificationally” should be replaced with some other word

RESPONSE:

Thanks for pointing out this issue. We have replaced “be used to quantificationally threshold” with “be used as quantitative reference to threshold” in line 364.

Excerpt (main text)

We added relevant descriptions to the **Results** (L360 – L365) section:

Results (L360 – L365):

> Notably, we observed a strong positive correlation (r = 0.622, Pearson’s correlation test; **Fig. 4a**) between the prediction accuracy (measured by AUC) and the RBP conservation based on 17 RBPs shared among mouse, zebrafish and fly, indicating that the degree of RBP conservation could be used as quantitative reference to threshold the prediction results of RBP–RNA interactions.

Figure S7 -> length

RESPONSE:

Thanks for pointing out this issue. We have corrected the typo in Figure S7*a* to “length.”

Reviewer #4 (Remarks on code availability)

The authors provide fully accessible code for all analyses, accompanied by rigorous and detailed documentation.

RESPONSE:

Thank you for carefully evaluating the code availability and documentation. In response, we have also updated the repository (https://github.com/GALE1228/MuSIC_pretrain) to include the revised version of MuSIC implementation.

Excerpt (main text)

We added relevant descriptions to the **Availability of data and materials** (L609 – L610) section:

Availability of data and materials (L609 – L610):

> An implementation of the MuSIC script and benchmarking data is available at https://github.com/GALE1228/MuSIC_pretrain.
